# Redox-active photocatalytic N^C cyclometalated gold(III) catecholate complexes

**DOI:** 10.3762/bjoc.22.97

**Published:** 2026-09-01

**Authors:** Miguel A Gonzálvez, Sergio Diaz-Alonso, Juan José Gamboa-Carballo, Karinne Miqueu, György Szalóki, Didier Bourissou

**Affiliations:** 1 Laboratoire Hétérochimie Fondamentale et Appliquée (LHFA, UMR 5069), CNRS/Université de Toulouse, 118 Route de Narbonne, 31062, Toulouse cedex 9, France; 2 Institut des Sciences Analytiques et de Physico-Chimie pour l’Environnement et les Matériaux (IPREM, UMR 5254), CNRS/Université de Pau et des Pays de l’Adour, Hélioparc, 2 Avenue du Président Angot, 64053 Pau Cedex 09, France

**Keywords:** catecholates, electron donor–acceptor complexes, gold, photoredox catalysis, redox-active ligands, semiquinones

## Abstract

The redox properties and photocatalytic activity of four N^C cyclometalated Au(III) catecholate complexes were investigated. They proved competent for the photocatalytic C–H arylation of furan with aryldiazonium salts under green LED irradiation. UV–vis studies supported the formation of electron donor–acceptor adducts between the Au(III) catecholate complexes and diazonium salts, enabling photoinduced electron transfer and aryl radical generation. Electrochemical, EPR, and DFT studies evidenced ligand-centered redox activity and the formation of Au(III) semiquinone species.

## Introduction

Over the last decade, two-electron chemistry of gold complexes, i.e., cycling between Au(I) and Au(III) redox states, has expanded remarkably, notably through the development of bidentate ligands such as P^C, P^P, P^N, and N^C scaffolds [1–4]. In contrast, one-electron processes remain extremely rare with gold complexes, despite the considerable potential of such open-shell pathways to unlock fundamentally distinct and unprecedented mechanistic manifolds [5–9]. Recently, we [10] discovered that P^C and P^P-ligated gold(III) catecholate complexes undergo photoinduced single-electron transfer (PET) with aryldiazoniums to generate synthetically useful aryl radicals, enabling the C–H arylation of furans and other heterocycles [11]. A pivotal feature underlying this reactivity is the redox-active character of the catecholate moiety, whose oxidation generates the key Au(III) semiquinone intermediate.

In parallel, the coordination chemistry and reactivity of Au(III) complexes have also undergone spectacular developments [12–13]. Despite their soft character, P-based ligands have proved efficient and versatile in stabilizing Au(III) complexes and supporting diverse transformations that were rarely observed before or even unprecedented in gold chemistry. However, N-based ligands, in particular N^C-cyclometalated ones, still occupy the forefront position in Au(III) chemistry [13–14]. Besides the harder character of N, which aligns well with the Au(III) oxidation state, N^C-cyclometalated ligands are readily installed at Au(III) and broadly vary structurally. Of note, the distinct electronic properties of N and P confer complementary behavior to N^C and P^C/P^P-ligated Au(III) complexes, as already documented in different systems [15–17]. To date, catalytic applications of cyclometalated Au(III) complexes mostly involve Lewis acid behavior of the metal and activation of π-substrates, with occasional contributions in two-electron redox coupling reactions. Among the previously reported (N^C)Au(III) catecholate complexes, only two studies have examined their catalytic activity, both in π-activation transformations [18–19].

**Figure 1:**
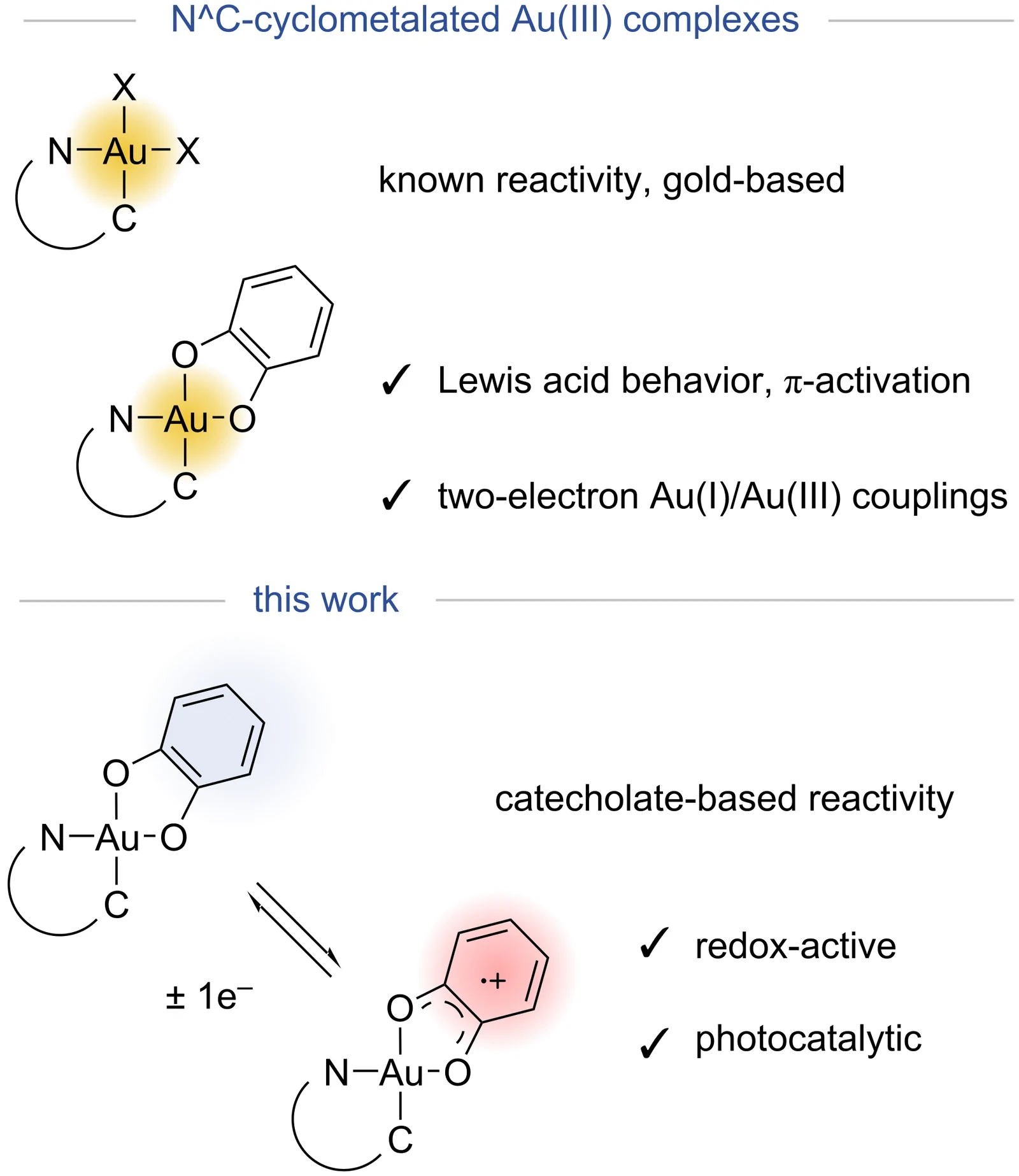
Catalytic applications of N^C-cyclometalated Au(III) complexes.

In the present work, we investigated whether N^C-cyclometalated Au(III) catecholate complexes could similarly display redox-active behavior and photocatalytic activity in the arylation of furan with diazonium salts (Figure 1) [20–21]. This would open a new reaction path for N^C Au(III) complexes, and comparison with the P^C and P^P-ligated systems would provide further insight into the influence of the ancillary ligand at Au(III).

## Results and Discussion

**Figure 2:**
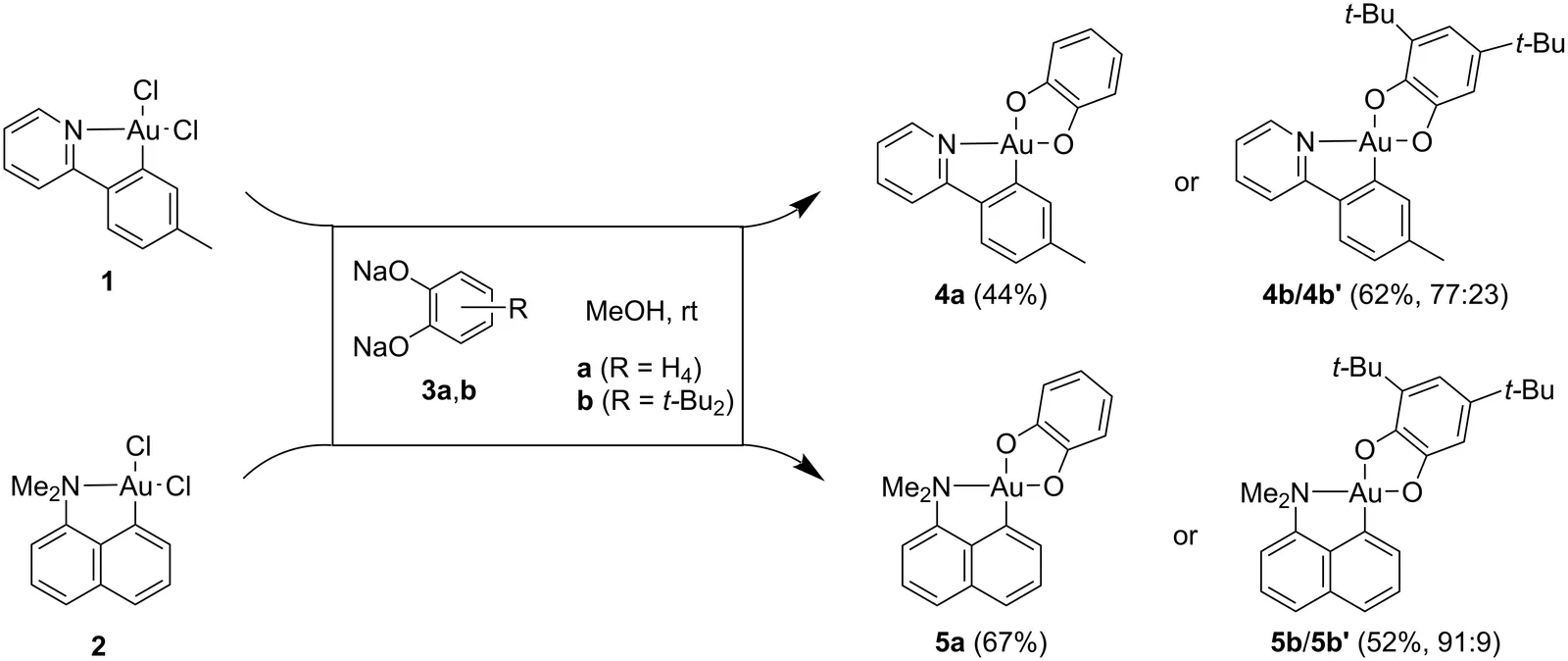
Preparation of the N^C-cyclometalated gold(III) catecholate complexes **4a**,**b** and **5a**,**b**. For the di-*tert*-butyl-substituted derivatives, only the major isomers **4b** and **5b** are depicted, and the isomer ratios are given in parentheses.

The four N^C-cyclometalated gold(III) catecholate complexes **4a**,**b** and **5a,b** were targeted in this study. Both the N^C ligand (a phenylpyridine or a naphthylamine) and the O^O ligand (parent and 3,5-di-*t*-Bu-substituted catecholates) were varied. Complexes **4a**,**b** and **5a,b** were conveniently synthesized via the same route as the P^C and P^P-ligated gold(III) complexes, namely through reaction of the corresponding Au(III) dihalogeno precursors **1** [22] and **2** [15] and sodium catecholates (Figure 2). They were obtained in moderate to good yields as air-stable solids. As expected because of the dissymmetry of the N^C ligand and di-*tert*-butyl catecholate moiety [23–24], complexes **4b** and **5b** were obtained as mixtures of *cis* and *trans*-isomers, whose stereochemistry was ascertained thanks to NOESY NMR experiments (see Supporting Information File 1, Figures S22 and S40). The robustness of this synthetic route foreshadows the possibility of synthesizing other Au(III) complexes bearing different ancillary ligands and catecholate moieties, offering a simple way to fine-tune and customize electrochemical and optical properties.

**Figure 3:**
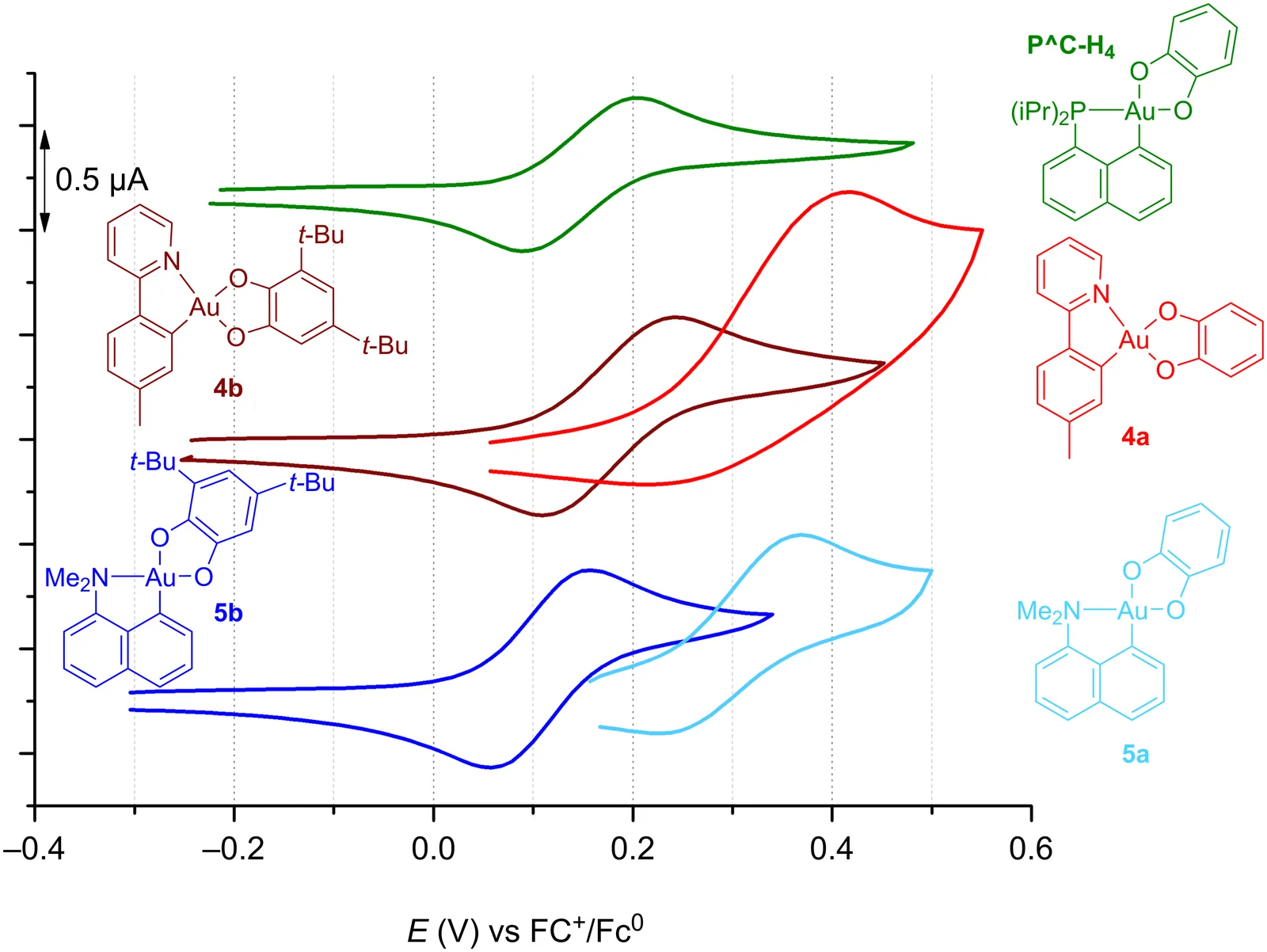
Cyclic voltammograms of **P^C-H_4_**, **4a**,**b**, and **5a**,**b** (0.25 mM, DCM, 0.25 M [Bu_4_N][PF_6_], 100 mV/s, Pt disk, Fc^+^/Fc^0^).

To assess the accessibility of the corresponding Au(III) semiquinone complexes, complexes **4a**,**b** and **5a**,**b** were then analyzed by cyclic voltammetry (Figure 3 and Table 1, see also Supporting Information File 1, Figure S8). These electrochemical studies revealed an overall behavior similar to that of the related P^C and P^P-ligated complexes [10], i.e., low-potential catecholate-centered oxidation, albeit with some noticeable differences as detailed hereafter. The oxidation of the N^C-cyclometalated parent catecholate Au(III) complexes **4a** and **5a** is shifted to higher potentials compared to the corresponding **P^C-H_4_** complex (Δ*E*_pa_ = +0.16 and +0.22 V, respectively). This probably reflects the weaker electron-donating nature of the N^C scaffold, as observed previously in Au(III) π-allyl complexes for example [17,25–26]. In addition, complexes **4a** and **5a** present an irreversible oxidation (even at 2000 mV/s scan rate) that suggests decreased stability of their oxidized forms. On the other hand, the more electron-rich di-*tert*-butylcatecholate complexes **4b** and **5b** exhibit oxidation potentials very similar to that of **P^C-H_4_** (Δ*E*_pa_ = ±0.05 V, Δ*E*_1/2_ = ±0.04 V) and increased stability of the ensuing oxidized forms. Here, the oxidation process is reversible as apparent from the *I*^pa^/*I*^pc^ current ratio (0.99 and 1.03 for **4b** and **5b**, respectively) [24]. These experimental data were further completed and corroborated by DFT calculations (see Supporting Information File 1, Table S8). Indeed, the ionization energies calculated for **4b** and **5b** are similar to that predicted for **P^C-H_4_** and substantially lower than that found for the parent catecholate complex **5a** (Table 1) [10].

**Table 1:** Corresponding half-wave potentials (*E*_1/2_), anodic potentials (*E*_pa_), current ratios (*I*^pa^/*I*^pc^), and ionization energies (*IE*) calculated at the SMD(DCM)-ZORA-PBE0-D4/SARC-ZORA-TZVP (Au), ZORA-def2-TZVP (other atoms) level of theory. n.d. for not determined.

Complex	*E*_1/2_ (V)	*E*_pa_ (V)	*I*^pa^/*I*^pc^	*IE* (kcal/mol)

**P^C-H** **_4_**	0.14	0.20	1.01	102.6
**4a**	n.d.	0.36	1.81	n.d.
**4b**	0.18	0.25	0.99	102.3
**5a**	n.d.	0.42	5.01	105.6
**5b**	0.10	0.15	1.03	101.5

The photocatalytic activity of the N^C-cyclometalated Au(III) catecholate complexes in the C–H arylation of furan with aryldiazonium salts was then investigated. The four complexes **4a**,**b** and **5a**,**b** were evaluated and compared under the same conditions (1 mol %, green light, 5 W, 20 °C) to **P^C-H_4_** as well as the benchmark catalyst eosin Y used by König et al. [10–11]. A small library of diazonium salts *p*-RPhN_2_^+^BF_4_^−^ with diverse electronic characters was employed (R = MeO, Me, H, F, Br, O_2_N). In each case, the reaction afforded the mono-2-arylated furan as the major product, with a minor amount of the 2,5-diarylated by-product (0–12%) (see Supporting Information File 1, Tables S1–S6). The four N^C-cyclometalated Au(III) complexes proved active (45% average yield over 24 reactions, Table 2). For the pristine diazonium salt PhN_2_^+^BF_4_^−^, complexes **4a**,**b**, and **5b** led to similar yields to **P^C-H_4_** and eosin Y (20–32%) but required longer reaction times (18 h instead of 6–8 h), while complex **5a** proved inferior (14% yield). With electron-deficient diazonium salts (*p*-F, *p*-Br, *p*-O_2_N), the N^C-cyclometalated Au(III) catecholate complexes all turned out to be less efficient than **P^C-H_4_** and eosin Y, giving lower yields and performing slower. Better results were obtained with the more challenging electron-rich diazonium salts (*p*-Me and *p*-MeO). Here, the di-*tert*-butylcatecholate complexes **4b** and **5b** performed best, approaching the results obtained with **P^C-H_4_** and eosin Y, i.e., 60% yield for *p*-MeOPhN_2_^+^BF_4_^–^ and 48% yield for *p*-MePhN_2_^+^BF_4_^–^ within 14 h with 1 mol % of **5b**. As for the P^C and P^P-ligated Au(III) complexes, substitution of the catecholate moiety by alkyl groups thus tends to improve the catalytic performance. We surmise this mainly results from increased stability of the corresponding Au(III) semiquinone complexes (as apparent from the CV studies). The formation of tighter electron donor–acceptor (EDA) adducts with the aryldiazonium substrate may also play a role (see below).

**Table 2:** Photocatalytic C–H arylation of furan. NMR yields (internal standard: 1,3,5-trimethoxybenzene) are given with the corresponding reaction times (at 100% conversion) in parentheses. Reaction times correspond to the point of maximum product yield (determined by ^1^H NMR).

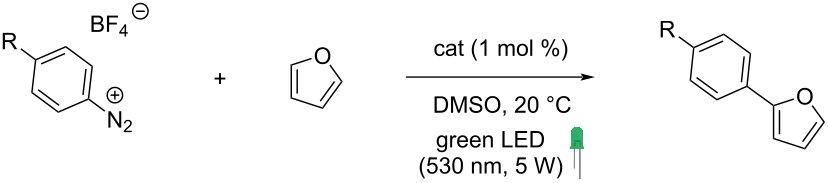						

Catalyst	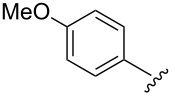	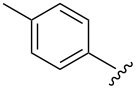	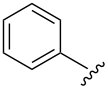	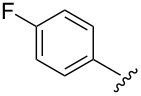	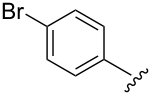	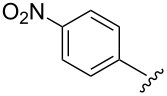

eosin Y	66 (10 h)	52 (10 h)	32 (6 h)	78 (1 h)	79 (2 h)	84 (20 min)
**P^C-H** **_4_**	65 (14 h)	30 (14 h)	25 (8 h)	79 (2 h)	81 (3 h)	82 (20 min)

**4a**	27 (24 h)	24 (18 h)	20 (18 h)	38 (12 h)	54 (6 h)	71 (1 h)
**4b**	54 (14 h)	46 (14 h)	31 (18 h)	49 (12 h)	70 (6 h)	67 (1 h)
**5a**	23 (18 h)	21 (18 h)	14 (18 h)	40 (6 h)	67 (6 h)	58 (1 h)
**5b**	60 (14 h)	48 (14 h)	23 (18 h)	52 (12 h)	73 (6 h)	54 (1 h)

**Figure 4:**
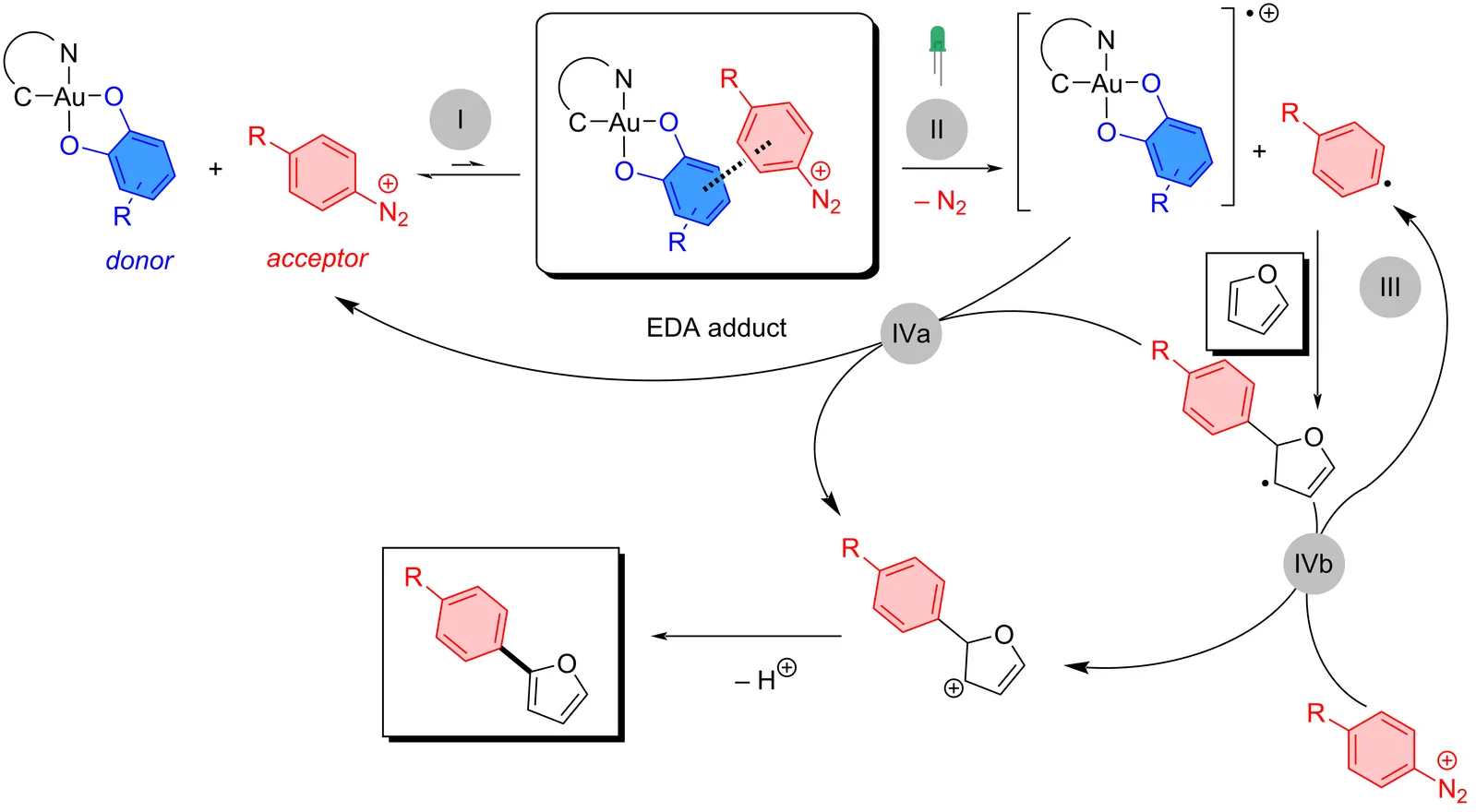
Mechanism proposed to account for the photocatalytic C–H arylation of furan with the N^C Au(III) catecholate complexes.

From a mechanistic viewpoint, we surmise that the N^C-cyclometalated complexes operate similarly to their P^C and P^P-ligated analogs [10], via a four-step sequence as depicted in Figure 4: I) formation of an EDA adduct between the Au(III) catecholate complex and the diazonium salt, II) photoinduced electron transfer (PET) from the Au(III) catecholate complex to the aryldiazonium followed by N_2_ dissociation, and release of the aryl radical, III) addition of the aryl radical to furan, IV) oxidation of the ensuing furanyl radical by a photoredox (IVa) or radical-chain (IVb) path, followed by deprotonation.

**Figure 5:**
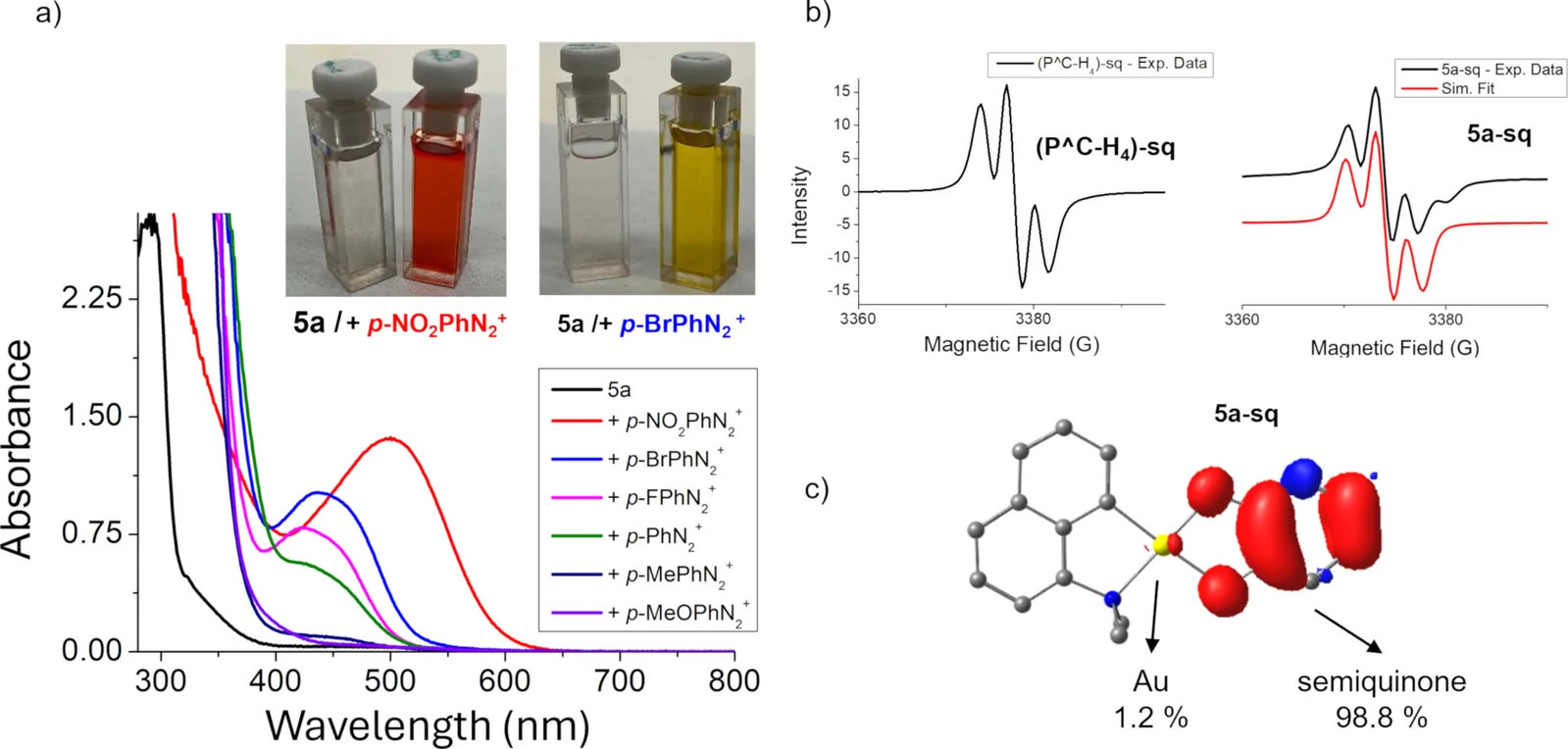
a) UV–vis spectra of the N^C-cyclometalated Au(III) catecholate complex **5a** (10^−3^ M) in the absence and in the presence of different aryldiazonium salts (10 equiv). Colors of the analytes are shown in the inset. b) X-band EPR spectra of **(P^C-H_4_)-sq** and **5a-sq** measured at 193 K (experimental data in black, fit in red). c) Calculated spin-density distribution in **5a-sq**.

To support this mechanistic scenario, we first investigated the ability of the N^C-cyclometalated Au(III) catecholate complexes to form EDA adducts with aryldiazoniums. Upon mixing **5a** with different diazonium salts (10 equiv), a distinct color change was observed in each case (to reddish with *p*-O_2_NPhN_2_^+^ and yellowish for *p*-BrPhN_2_^+^, for example) which was accompanied by a clearly identifiable charge-transfer band in the UV–vis spectra (Figure 5a). In line with the donor–acceptor nature of the formed adducts, the red-shift of the absorption band is the largest with the most electron-deficient *p*-O_2_NPhN_2_^+^BF_4_^–^ salt. In addition, the 1:1 stoichiometry of the EDA adduct was established in the case of *p*-BrPhN_2_^+^BF_4_^–^ thanks to a UV–vis Job plot experiment (see Supporting Information File 1, Figure S9).

Next, we aimed to generate and characterize N^C-cyclometalated Au(III) semiquinone complexes. The one deriving from oxidation of the parent catecholate complex **4a** with the *p*-tolylpyridine ligand proved too unstable to be simply observed by EPR spectroscopy, in line with the irreversible oxidation wave observed by CV. Gratifyingly, the Au(III) semiquinone complexes **4b-sq**, **5a-sq**, and **5b-sq** could be conveniently prepared by chemical oxidation (using AgBF_4_) and successfully characterized by EPR spectroscopy (see Supporting Information File 1, Figures S5–S7). The EPR spectrum of **5a-sq** is shown in Figure 5b, along with that of the analogous **(P^C-H_4_)-sq** complex, for comparison [10]. The obtained triplet-like signal was conveniently fitted using EasySpin. The obtained values (*g*_iso_ = 1.9998, *A*_H1_ = 2.8 MHz, *A*_H2_ = 8.1 MHz, *A*_H3_ = 6.8 MHz, *A*_H4_ = 1.7 MHz) match well those computed by DFT calculations (*g*_iso_ = 1.9992, *A*_H1_ = 2.85 MHz, *A*_H2_ = 13.9 MHz, *A*_H3_ = 8.6 MHz, *A*_H4_ = 1.1 MHz) and confirm the Au(III) semiquinone nature of the oxidized complex. This picture is further supported by the optimized geometry of **5a-sq** which corresponds to a metrical oxidation state (MOS) [27] value of −1.10(5). Consistently, the computed spin-density distribution (Figure 5c) shows spin concentration on the semiquinone (98.8%), negligible contribution of gold (1.2%), and no contribution of the N^C ligand. Interestingly, the electronic dissymmetry of the N^C ligand (stronger *trans* influence of C versus N) translates into the Au–O bond lengths (that differ by >10 pm) but not in the semiquinone moiety that remains essentially symmetric, both geometrically and electronically (see the O–C/C–C bond lengths and atomic spin populations, Table S7 and Figure S11 in Supporting Information File 1).

## Conclusion

Four N^C-cyclometalated Au(III) catecholate complexes were prepared and their redox properties and photocatalytic activity examined. Cyclic voltammetry and EPR spectroscopy revealed catechol-based redox-active character. According to DFT calculations, the geometry of the semiquinone moiety and the associated spin distribution remain largely unperturbed, despite the electronic dissymmetry of the N^C ligand. The N^C-cyclometalated Au(III) catecholate complexes proved competent catalysts for the C–H arylation of furan with aryldiazonium salts under green LED irradiation. They do not compete with P^C and P^P-ligated systems, but di-*tert-*butyl substitution of the catecholate moiety improves performance.

These results demonstrate that N^C-cyclometalated Au(III) catecholate complexes can engage in PET processes and constitute a promising platform for open-shell photocatalysis. Given the high modularity of both the redox-active catecholate ligand and the ancillary N-based ligand, further optimization should enable to improve catalytic efficiency and expand the scope of such PET-based catalytic transformations. To this end, several factors come into play and should be taken into account, in particular the stability of the Au(III) semiquinone complexes, and the properties of the EDA adduct formed between the Au(III) catecholate complex and the aryldiazonium salt.

## Supporting Information

File 1 Experimental procedures, analytical data, computational details, Cartesian coordinates of the computed structures, cyclic voltammograms, UV–vis, EPR, and NMR spectra.

## Data Availability

All data that supports the findings of this study is available in the published article and/or the supporting information of this article.
