# Supplementary material for: Redox-active photocatalytic N^C cyclometalated gold(III) catecholate complexes
Source: Beilstein J Org Chem. 2026 Sep 1;22:1214–20. doi: 10.3762/bjoc.22.97 (PMC13575342; doi:10.3762/bjoc.22.97)
Supplement: Supplementary file 1 [file Beilstein_J_Org_Chem-22-1214-s001.pdf]

BEILSTEIN JOURNAL OF ORGANIC CHEMISTRY

Supporting Information

for

Redox-active photocatalytic N^C cyclometalated gold(III) catecholate complexes

Miguel A. Gonzálvez, Sergio Diaz-Alonso, Juan José Gamboa-Carballo, Karinne Miqueu, György Szalóki and Didier Bourissou

Beilstein J. Org. Chem. **2026,** *22,* 1214–1220. doi:10.3762/bjoc.22.97

Experimental procedures, analytical data, computational details, Cartesian coordinates of the computed structures, cyclic voltammograms, UV–vis, EPR, and NMR spectra

License and Terms: This is a supporting information file under the terms of the Creative Commons Attribution License (https://creativecommons.org/licenses/by/4.0). Please note that the reuse, redistribution and reproduction in particular requires that the author(s) and source are credited and that individual graphics may be subject to special legal provisions.
The license is subject to the *Beilstein Journal of Organic Chemistry* terms and conditions: (https://www.beilstein-journals.org/bjoc/terms)

Table of Contents

1 Materials & Methods **S3**

1.1 Experimental Methods

1.2 Computational Methods

2 Synthesis of N^C Catechol and Semiquinone Au Complexes **S6**

2.1 Synthesis of Au(III) N^C catecholate complexes

2.2 Synthesis of Au(III) N^C semiquinone complexes

2.3 UV-Vis spectra

2.4 EPR Spectra

2.5 Cyclic Voltammetry Data

3 EDA Complexation with aryldiazoniums as acceptors **S13**

3.1 Job plot experiments (EDA stoichiometry)

4 C–H arylation reactions of furan **S14**

5 Computational Studies **S18**

6 NMR Spectra **S21**

7 xyz coordinates and energies in a.u. **S39**

8 References **S43**

1. Materials & Methods

1.1 Experimental Methods

All reactions and sample handling were performed under dry argon using standard Schlenk techniques or an inert-atmosphere glovebox unless noted. Commercial reagents were used as received. Solvents were rigorously dried and degassed by the freeze-pump-thaw technique before use.

Solution NMR spectra (^{1}H, ^{13}C) were collected at 298 K on Bruker Avance instruments operating at 300, 500 or 600 MHz. Chemical shifts (δ) are reported in ppm. Residual solvent signals served as internal references for ^{1}H and ^{13}C spectra. For catalytic tests, 1,3,5-trimethoxybenzene (internal standard) was added at full conversion, as determined by ^{1}H NMR monitoring. Signal descriptions follow the usual abbreviations: br, broad; s, singlet; d, doublet; t, triplet; m, multiplet. Resonance assignments were supported by HSQC, HMBC, and NOESY experiments.

High-resolution mass spectra were obtained on Waters UPLC Xevo G2 QTOF.

Cyclic voltammetry was carried out in a 10 mL glass electrochemical cell using a PGSTAT204 potentiostat. Measurements were performed in dry CH_2Cl_2 (HPLC-grade) at ambient temperature. A platinum disk (d = 1.6 mm, Biologic) served as the working electrode, a platinum sheet (~1 cm^2, Metrohm) as the counter electrode, and a platinum wire confined in a glass frit with the supporting electrolyte as pseudo-reference electrode. The supporting electrolyte was dry tetrabutylammonium hexafluorophosphate ($[Bu_4N][PF_6]$, electrochemistry grade, Sigma-Aldrich) at a concentration of 0.25 M. Ferrocene (0.25 mM in 0.25 M $[Bu_4N][PF_6]$) was used as an internal standard both before and after measuring the analyte.

UV-Vis spectra were recorded on a Cary 60 spectrophotometer. All optical measurements were performed using a quartz cuvette (path length, 1 cm) at ambient temperature in the specified solvent.

X-band EPR data were recorded using an ELEXSYS E500 EPR spectrometer from Bruker operating at a microwave frequency of approximately 9.5 GHz. Samples were prepared in quartz capillary tubes (inner diameter: 1.0 mm, outer diameter: 1.25 mm). Measurements were conducted at 193 K, unless specified, using a nitrogen gas flow cryostat. Typical experimental parameters were as follows: microwave power, 3.21 mW; modulation frequency, 100 kHz; modulation amplitude, 0.1 or 1.0 G (specified for each sample); conversion time, 10 ms; sweep width, 30.0 G; center field, 3378 G; number of scans, 4–16. EPR spectral simulations were performed using the EasySpin toolbox (version 6.06)[1] running under MATLAB (version R2024b, MathWorks).[2] Isotropic g-values and A-tensors simulations were fitted using the ‘garlic’ function and the ‘Nelder–Mead’ method.

1, **2** and **3a,b** were synthesized according to reported protocols.[3-5]

1.2 Computational Methods

Density functional theory (DFT) calculations, including optimizations and frequency calculations, were performed using Gaussian 16 package, revision B.01.[6] Wavefunctions were further refined for electronic energy calculations and the determination of electronic properties using ORCA version 6.0.1.[7] Solvent effects were included implicitly in all calculations using the universal Solvation Model based on Density (SMD) method[8] with dichloromethane as solvent. Geometry optimizations were performed using the PBE0 hybrid functional[9] in combination with Grimme's DFT-D3 dispersion corrections[10] containing Becke-Johnson damping (BJ)[11] and the 6-31G** Pople's double-ζ basis set.[12] The gold atom was described with the relativistic electron core potential (ecp-60-mwb, named SDD)[13] and the associated basis set, augmented by a set of f-orbital polarization function.[14] This method is henceforth referred as SMD(DCM)-PBE0-D3(BJ)/SDD+f(Au), 6-31G**(other atoms) level of theory. The nature of each stationary point was confirmed by frequency calculations with no imaginary frequencies for the obtained *minima*.

Single-point calculations on the previously optimized structures were performed to refine the electronic energies and wavefunctions (molecular orbitals and spin densities), and to compute electron paramagnetic resonance (EPR) parameters using the GIAO formalism[15], as well as the ionization potentials (IP). We used the PBE0 hybrid functional[9] with Grimme's DFT-D4[16] dispersion corrections. Relativistic effects were estimated using the Zeroth-Order Regular Approximation (ZORA).[17] The triple-ζ Karlsruhe basis set def2-TZVP[18] was used for light elements, while the segmented all-electron relativistically contracted triple-ζ (SARC-TZVP)[19] basis set was employed for Au. Both basis sets were used recontracted for ZORA as implemented in ORCA 6.0.1 (explicitly ZORA-def2-TZVP and SARC-ZORA-TZVP, respectively). Resolution of identity (RI)[20] was used through the RIJCOSX algorithm[21] to speed up the calculations at a negligible accuracy cost by employing the corresponding Coulomb fitting SARC/J[19] for the gold atom and def2/J[22] for the rest. This methodology is referred herein as SMD(DCM)-ZORA-PBE0-D4/SARC-TZVP(Au), def2-TZVP(other atoms)//SMD(DCM)-PBE0-D3(BJ)/SDD+f(Au), 6-31G**(other atoms). We reported the isotropic g-factors (g_{iso}) and the hyperfine coupling constants (A_H, in MHz). The rendering and visualization of orbitals, as well as the plotting of surfaces were performed using Chemcraft version 1.8.[23]

Numbering of the compounds used in this study

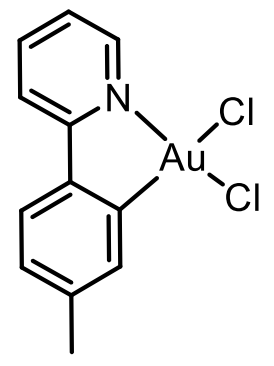

1

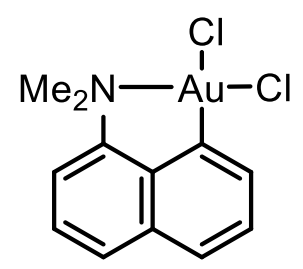

2

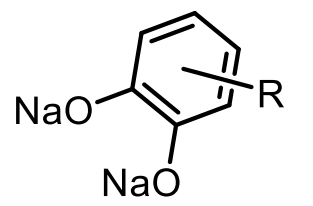

3a,b R = H_4 (**a**)
o,*m*-*t*Bu_2 (**b**)

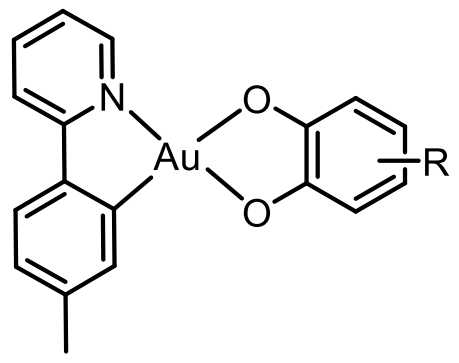

4a,b R = H_4 (**a**)
o,*m'*-*t*Bu_2 (**b**)

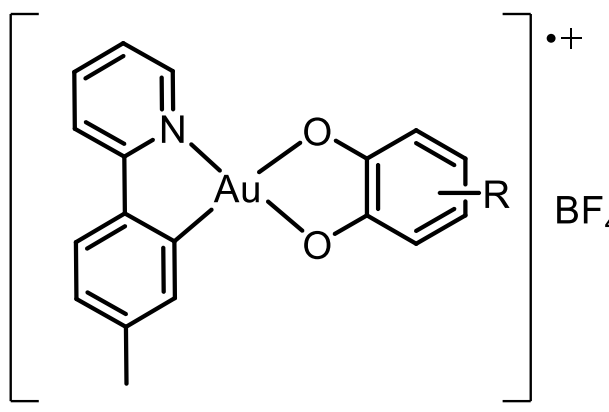

4a,b-sq

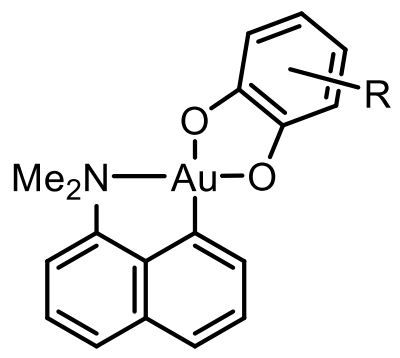

5a,b R = H_4 (**a**)
o,*m*-*t*Bu_2 (**b**)

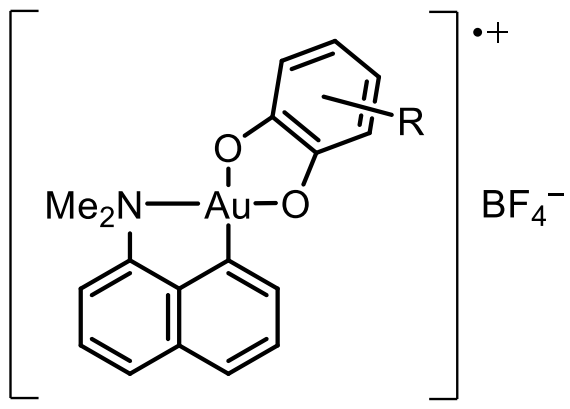

5a,b-sq

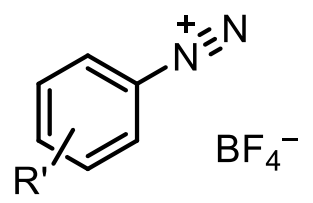

6.1-6.6

R' = NO_2, Br, F, H, MeO, Me

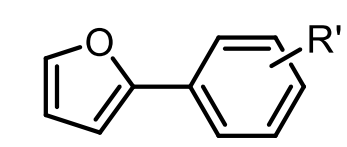

7.1-7.6

C–H arylation products

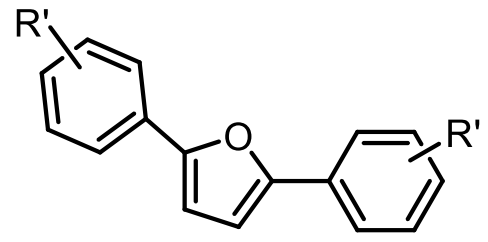

8.1-8.6

2. Synthesis of N^C Catechol and Semiquinone Au Complexes

2.1 Synthesis of Au(III) N^C catecholate complexes

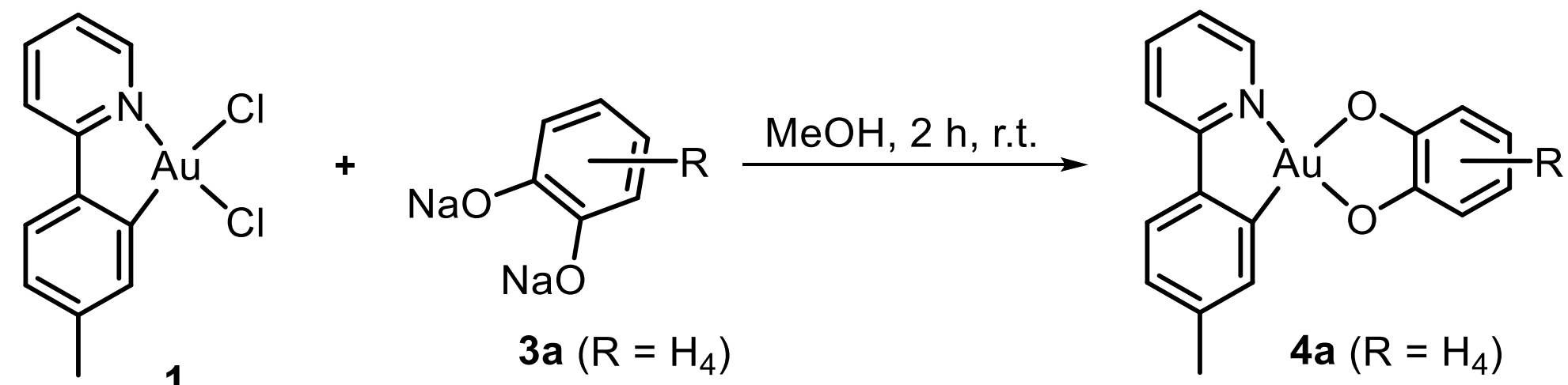

1

3a (R = H_4)
3b (R = $^t Bu_2$)

4a (R = H_4)
4b (R = $^t Bu_2$)

In a dry Schlenk under an argon atmosphere, complex **1** (65.4 mg, 0.15 mmol, 1 equiv.) was combined with the required sodium catecholate **3** (0.30 mmol, 2 equiv.) in deoxygenated MeOH (1 mL). The mixture was stirred vigorously for 2 hours at room temperature under argon. A precipitate appeared in the reaction; the reaction was filtered through a Büchner funnel and the solid was washed with MeOH (3 × 2 mL). The isolated material was then dried under vacuum for 1 hour to afford the desired catecholate gold complex.

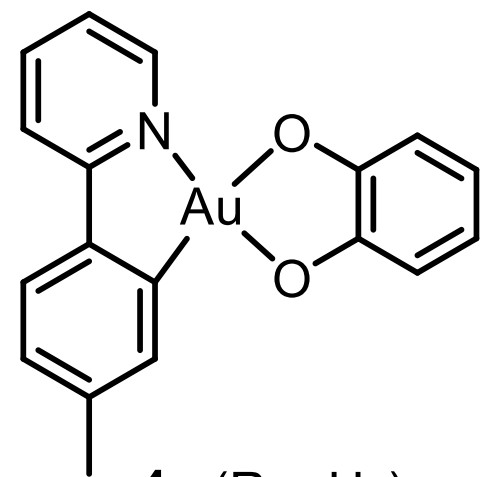

4a (R = H_4)

4a. Obtained as a red solid (44 %). **^{1}H NMR** (500 MHz, CD_2Cl_2): δ 8.89 (dd, J = 6.0, 1.5 Hz, $C\text{H}_{\text{PyridineRing}}$, 1H), 7.94 (td, J = 7.9, 1.7 Hz, $C\text{H}_{\text{PyridineRing}}$, 1H), 7.69 (d, J = 8.1 Hz, $C\text{H}_{\text{PyridineRing}}$, 1H), 7.42 – 7.35 (m, 2 $C\text{H}_{\text{PhenylRing}}$, 2H), 7.29 (ddd, J = 7.3, 5.8, 1.4 Hz, $C\text{H}_{\text{PyridineRing}}$, 1H), 7.13 (dd, J = 7,8, 1 Hz, $C\text{H}_{\text{PhenylRing}}$, 1H), 6.63 (dd, J = 7.7, 1.6 Hz, $C\text{H}_{\text{Cat}}$, 1H), 6.57 (dd, J = 7.7, 1.6 Hz, $C\text{H}_{\text{Cat}}$, 1H), 6.51 (td, J = 7.4, 1.6 Hz, $C\text{H}_{\text{Cat}}$, 1H), 6.45 (td, J = 7.4, 1.6 Hz, $C\text{H}_{\text{Cat}}$, 1H), 2.40 (s, CH_3 Phenyl ring, 3H). **^{13}C{^{1}H} NMR** (126 MHz, CD_2Cl_2): δ ^{13}C NMR (126 MHz, CD_2Cl_2) δ 164.83 $C_{\text{qPyridineRing}}$, 162.26 C_{qCat}, 158.90 C_{qCat}, 148.26 $C\text{H}_{\text{PyridineRing}}$, 145.54 $C_{\text{qPhenylRing}}$, 143.34 $C_{\text{qPhenylRing}}$, 142.55 $C\text{H}_{\text{PyridineRing}}$, 139.19 $C_{\text{qPhenylRing}}$, 130.39 $C\text{H}_{\text{PhenylRing}}$, 129.33 $C\text{H}_{\text{PhenylRing}}$, 124.93 $C\text{H}_{\text{PhenylRing}}$, 123.84 $C\text{H}_{\text{PyridineRing}}$, 120.74 $C\text{H}_{\text{PyridineRing}}$, 119.29 $C\text{H}_{\text{Cat}}$, 117.29 $C\text{H}_{\text{Cat}}$, 116.95 $C\text{H}_{\text{Cat}}$, 115.48 $C\text{H}_{\text{Cat}}$, 22.08 (CH_3 Phenyl ring). **HRMS** (ESI+) calcd. for $[MH]^+$ = $C_{18}H_{15}O_2NAu$: 474.0763, found: 474.0769.

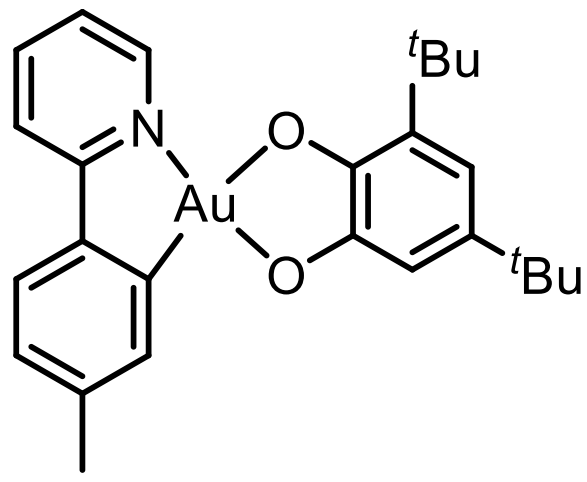

4b

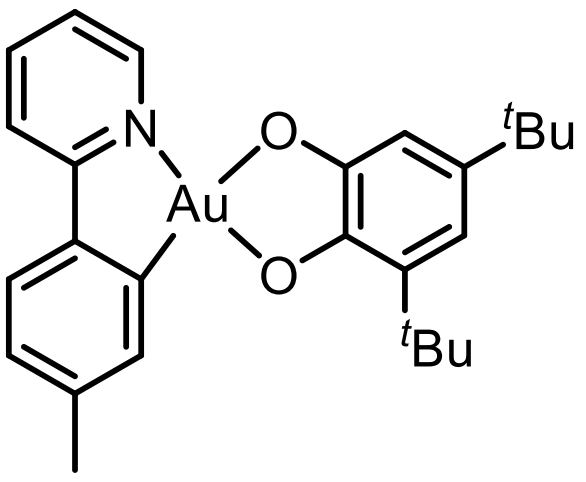

4b'

4b/4b'. Obtained as a red solid (62 %). Mixture of diastereoisomers 77% of **4b** and 23% of **4b'** (the isomers were assigned by ^{1}H-^{1}H NOESY experiments). **^{1}H NMR** (500 MHz, CD_2Cl_2): δ 9.02 (d, J = 4.4, $C\text{H}_{\text{PhenylRing}}$, 1H), 8.02 (t, J = 7.9 Hz, $C\text{H}_{\text{PhenylRing}}$, 1H), 7.80 (d, J = 8.2 Hz, $C\text{H}_{\text{PhenylRing}}$, 1H), 7.49 (s, $C\text{H}_{\text{PhenylRing}}$, 1H), 7.45 (d, J = 7.9 Hz, $C\text{H}_{\text{PhenylRing}}$, 1H), 7.39 (t, J = 6.0 Hz, $C\text{H}_{\text{PhenylRing}}$, 1H), 7.17 (d, J = 7.0 Hz, $C\text{H}_{\text{PhenylRing}}$, 1H), 6.65 (t, 1.6 Hz, $C\text{H}_{\text{Cat}}$, 1H), 6.61 (d, J = 2.3 Hz, $C\text{H}_{\text{Cat}}$, 1H), 2.44 (s, CH_3 Phenyl ring, 3H), 1.51 (s, 3 CH_3 of ^{t}Bu, 9H), 1.30 (s, 3 CH_3 of ^{t}Bu, 9H). **^{13}C{^{1}H} NMR** (126 MHz, CD_2Cl_2): δ 165.25 $C_{\text{qPyridineRing}}$, 161.92 C_{qCat}, 154.72 C_{qCat}, 148.19 $C\text{H}_{\text{PyridineRing}}$, 146.30 $C_{\text{qPhenylRing}}$, 143.47 $C_{\text{qPhenylRing}}$, 142.53 $C\text{H}_{\text{PyridineRing}}$, 139.36 2 C_q (C_{qCat} + $\text{C}_{\text{qPhenylRing}}$), 136.56 C_{qCat}, 130.76 $C\text{H}_{\text{PhenylRing}}$, 129.15 $C\text{H}_{\text{PhenylRing}}$, 124.95 $C\text{H}_{\text{PhenylRing}}$, 123.92 $C\text{H}_{\text{PyridineRing}}$, 120.82 $C\text{H}_{\text{PyridineRing}}$, 113.15 $C\text{H}_{\text{Cat}}$, 111.24 $C\text{H}_{\text{Cat}}$, 35.02 (C_q ^{t}Bu), 34.35 (C_q ^{t}Bu), 32.19 (3 CH_3 of ^{t}Bu), 29.98 (3 CH_3 of ^{t}Bu), 22.09 (CH_3 Phenyl ring). **HRMS** (ESI+) calcd. for $[M+H]^+$ = $C_{26}H_{31}O_2NAu$: 586.2015, found 586.2028.

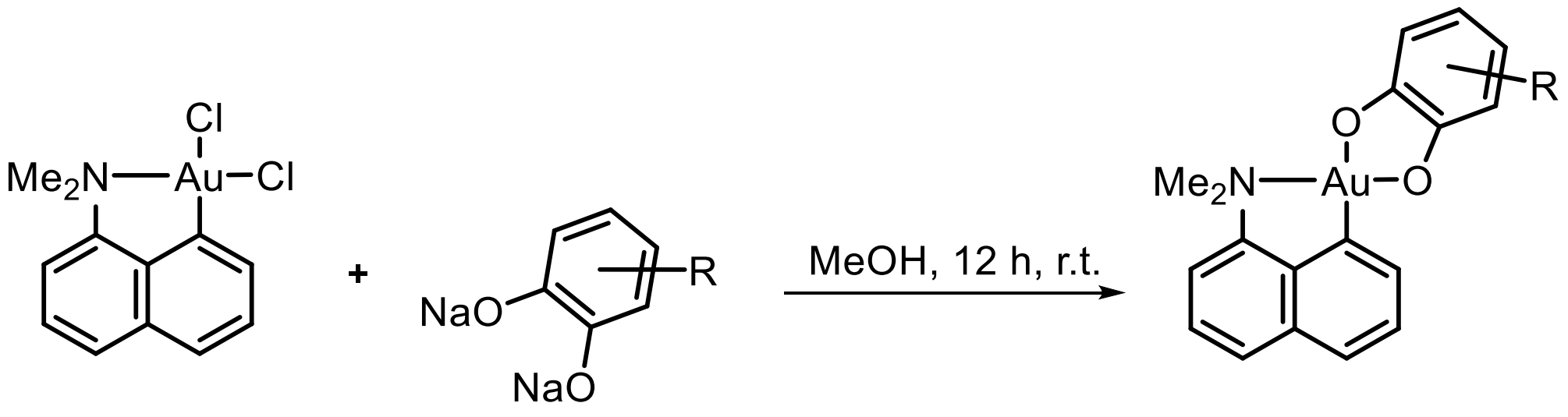

2 **3a** (R = H_4) **3b** (R = ^{t}Bu$_2$) **5a** (R = H_4) **5b** (R = ^{t}Bu$_2$)

In a dry Schlenk under an argon atmosphere, complex **2** (65.7 mg, 0.15 mmol, 1 equiv.) was combined with the required sodium catecholate **3** (0.30 mmol, 2 equiv.) in deoxygenated MeOH (1 mL). The mixture was stirred vigorously for 12 hours at room temperature under argon. A precipitate appeared in the reaction; the reaction was filtered through a Büchner funnel and the solid was washed with MeOH (3 × 2 mL). The isolated material was then dried under vacuum for 1 hour to afford the desired catecholate gold complex.

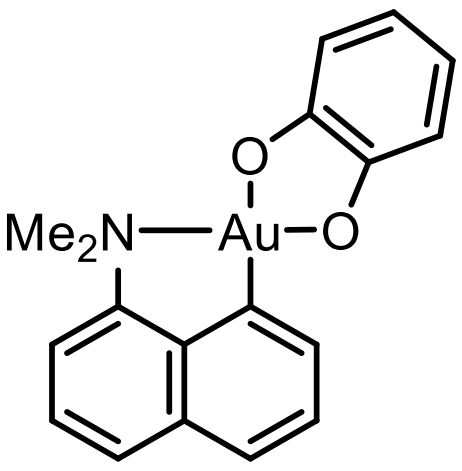

5a (R = H_4)

5a. Obtained as a pink solid (67 %). **^{1}H NMR** (600 MHz, CD_2Cl_2): δ 7.81 (d, *J* = 8.0 Hz, CH_{Naphth}, 1H), 7,80 (d, J = 8.1 Hz, CH_{Naphth}, 1H) 7.68 (dd, *J* = 7.2, 1.0 Hz, CH_{Naphth}, 1H), 7.56 (t, *J* = 7.9 Hz, CH_{Naphth}, 1H), 7.53 – 7.47 (m, 2 CH_{Naphth}, 2H), 6.73 (dd, *J* = 7.7, 1.5 Hz, CH_{Cat}, 1H), 6.61 (dd, *J* = 7.7, 1.5 Hz, CH_{Cat}, 1H), 6.54 (td, *J* = 7.4, 1.5 Hz, CH_{Cat}, 1H), 6.48 (td, *J* = 7.4, 1.5 Hz, CH_{Cat}, 1H), 3.79 (s, 2 *C*H_3 of NMe_2, 6H). **^{13}C{^{1}H} NMR** (151 MHz, CD_2Cl_2): δ 161.51 C_{qCat}, 158.64 C_{qCat}, 153.26 $C_{qNaphth}$, 138.29 $C_{qNaphth}$, 137.36 $C_{qNaphth}$, 135.23 $C_{qNaphth}$, 129.15 CH_{Naphth}, 127.96 CH_{Naphth}, 127.00 CH_{Naphth}, 126.50 CH_{Naphth}, 126.47 CH_{Naphth}, 119.38 CH_{Cat}, 117.52 CH_{Cat}, 117.09 CH_{Naphth}, 116.79 CH_{Cat}, 115.12 CH_{cat}, 56.50 (2 *C*H_3 of NMe_2). **HRMS** (ESI+) calcd. for $[M+H]^+$ = $C_{18}H_{17}O_2NAu$: 476.0920, found 476.0935.

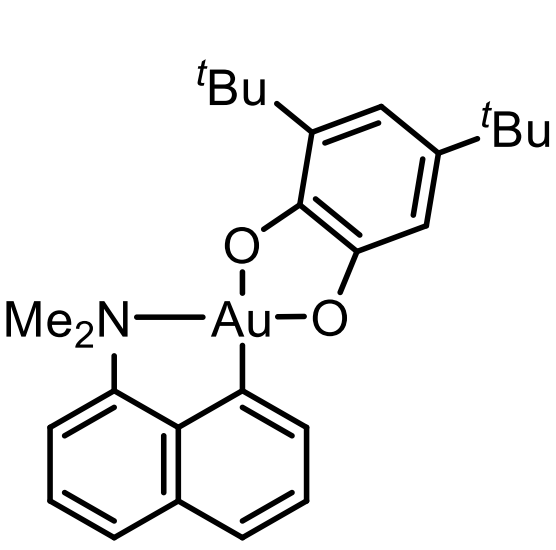

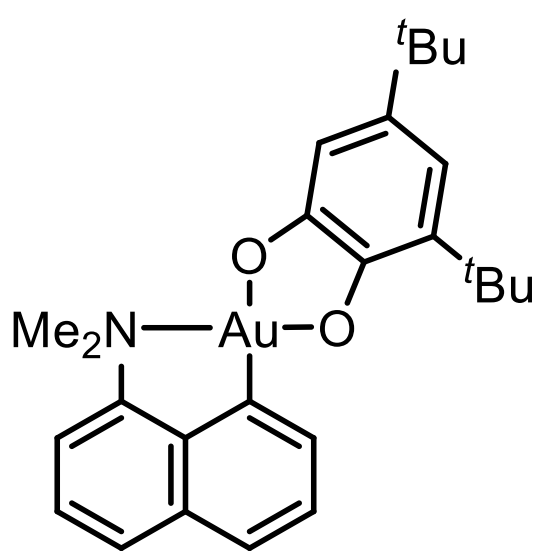

5b **5b'**

5b/5b'. Obtained as a pink solid (52 %). Mixture of isomers (91% **5b** / 9% **5b'**) (the isomers were assigned by ^{1}H-^{1}H NOESY experiments). **^{1}H NMR** (500 MHz, CD_2Cl_2): δ 7.80 (ddd, *J* = 8.2, 5.6, 0.9 Hz, 2 CH_{Naphth}, 2H), 7.67 (dd, *J* = 7.1, 1.0 Hz, CH_{Naphth}, 1H), 7.55 (t, J = 7,6 Hz, CH_{Naphth}, 1H), 7.54 – 7.45 (m, 2 CH_{Naphth}, 2H), 6.69 (d, *J* = 2.3 Hz, CH_{Cat}, 1H), 6.61 (d, *J* = 2.3 Hz, CH_{Cat}, 1H), 3.79 (s, 6H, 2 *C*H_3 of NMe_2), 1.49 (s, 3 *C*H_3 of ^{t}Bu, 9H), 1.29 (s, 3 *C*H_3 of ^{t}Bu, 9H). **^{13}C{^{1}H} NMR** (126 MHz, CD_2Cl_2): 161.11 C_{qCat}, 154.23 C_{qCat}, 153.24 $C_{qNaphth}$, 139.63 C_{qCat}, 138.68 $C_{qNaphth}$, 138.11 $C_{qNaphth}$, 136.28 C_{qCat}, 135.22 $C_{qNaphth}$, 129.12 CH_{Naphth}, 127.98 CH_{Naphth}, 126.86 CH_{Naphth}, 126.71 CH_{Naphth}, 126.21 CH_{Naphth}, 117.07 CH_{Naphth}, 113.13 CH_{Cat}, 110.88 CH_{Cat}, 56.46 (2 CH_3 of NMe_2), 34,99 (C_q ^{t}Bu), 34,38 (C_q ^{t}Bu), 32.18 (3 CH_3 of ^{t}Bu), 29.89 (3 CH_3 of ^{t}Bu). **HRMS** (ESI+) calcd. for $[M+H]^+$ = $C_{26}H_{33}O_2NAu$: 588.2172, found: 588.2190.

2.2 Synthesis of Au(III) N^C semiquinone complexes

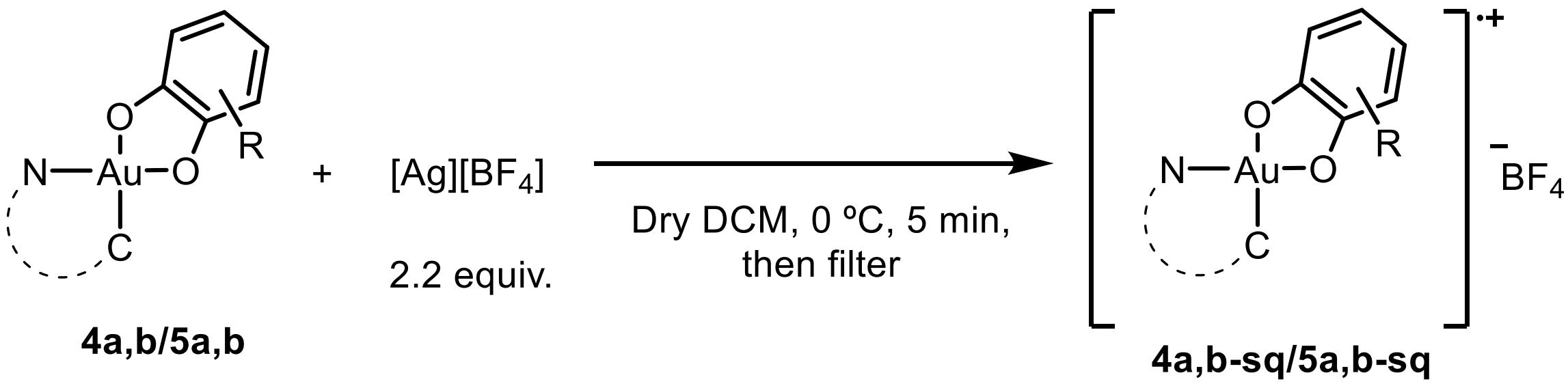

0.3 mmol of complex was dissolved in a round-bottom flask containing 1 mL of dry DCM, and the solution was cooled to 0 °C. Then, 0.65 mmol of $AgBF_4$ were added in one portion, and the mixture was stirred for 5 minutes. Following the colour change (yellow for **4b**, green for **5a** and red for **5b**), the mixture was filtered using either a PTFE 13 mm 0.2 μm syringe HPLC filter (Agilent) or through a pipette filled with rigorously dry Celite®. The yields were quantitative.

The complex **4a** from the pyridine family decomposed immediately upon oxidation, even when carrying out the reaction at – 80 °C; a dark precipitate was instantly formed.

2.3 UV-Vis Data

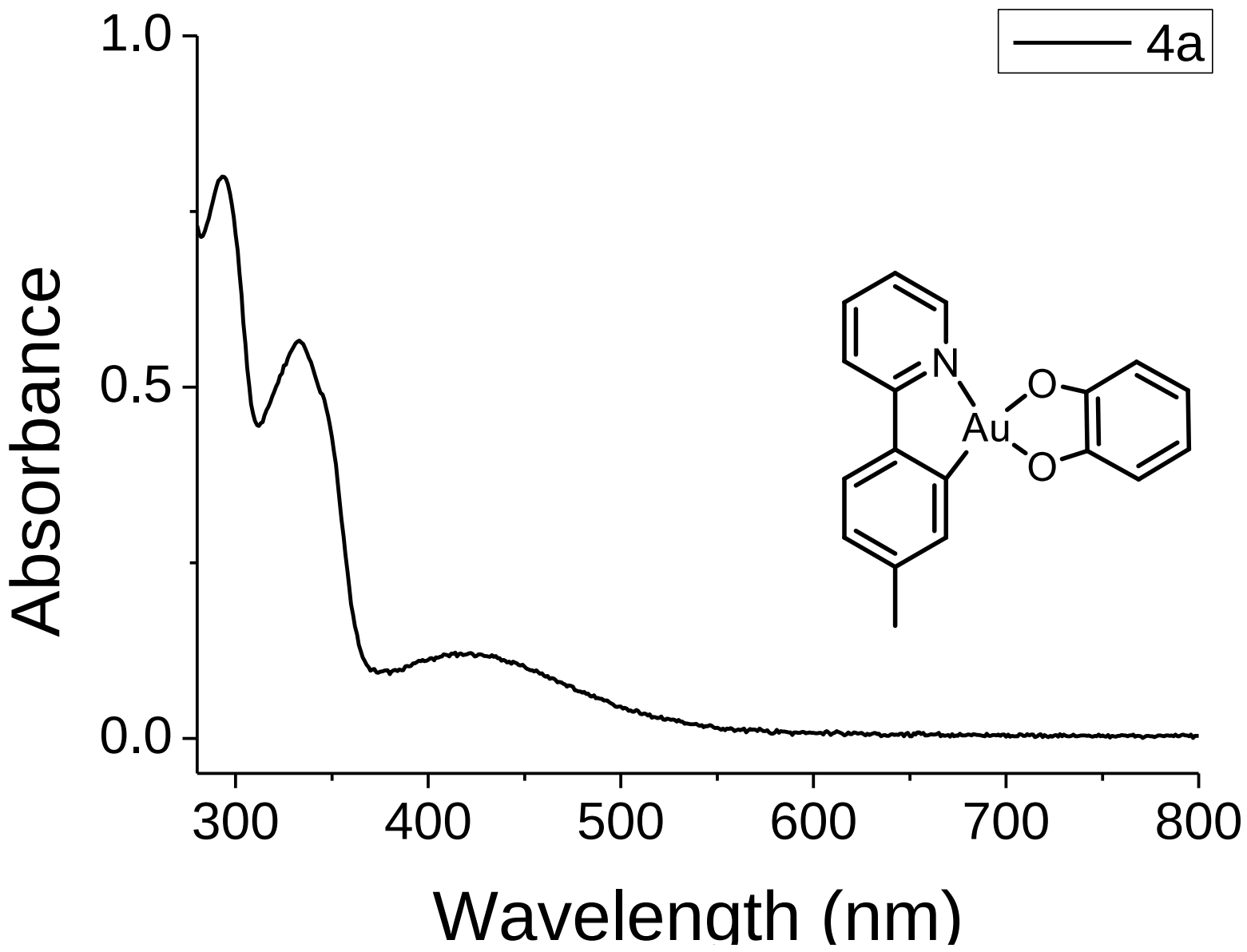

Figure S1. UV-Vis spectra of **4a** (black) DCM solution (10^{-4} M) at 298 K.

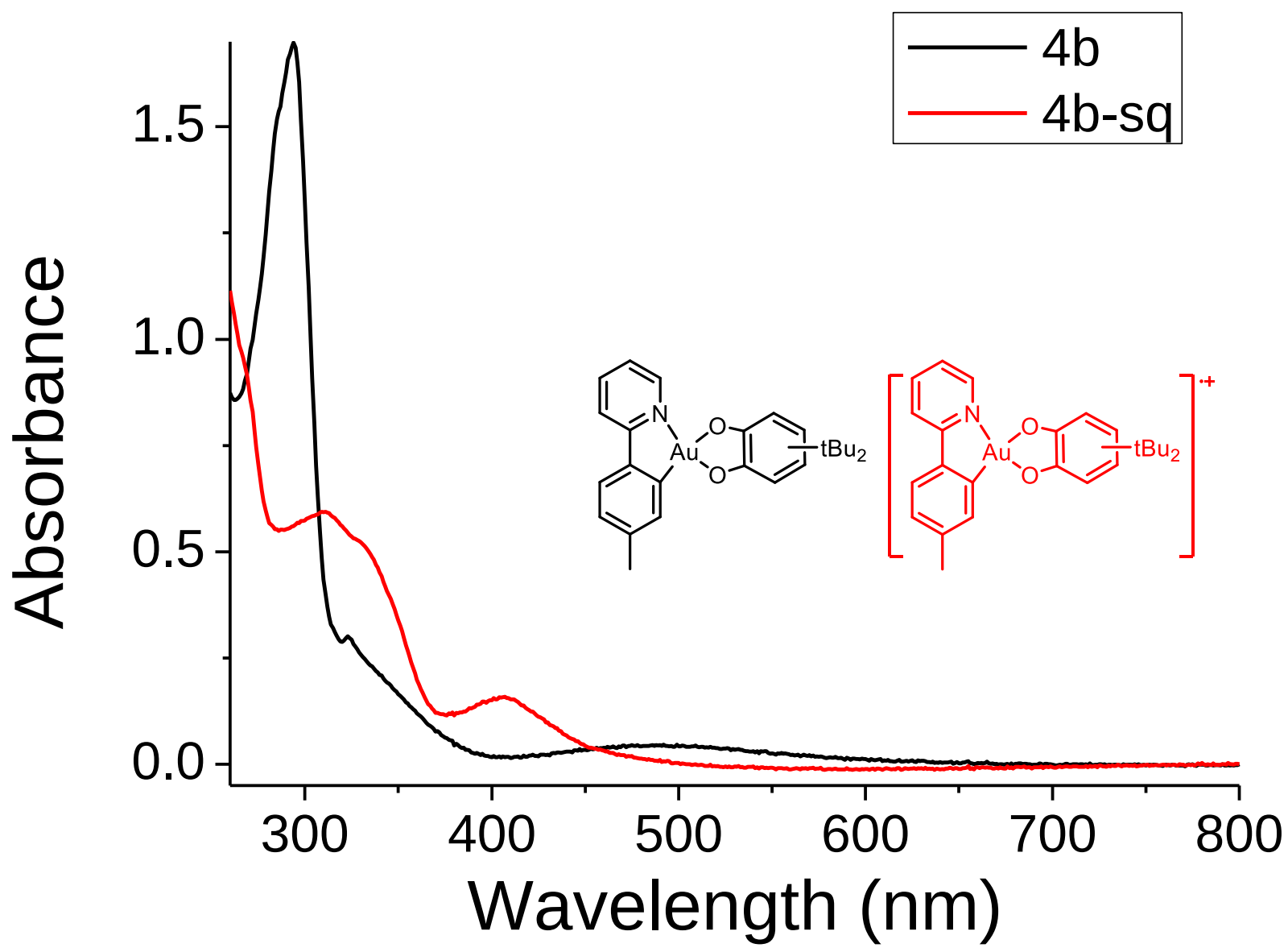

Figure S2. UV-Vis spectra of **4b** (black) and **4b-sq** (red) in DCM solution (10^{-3} M) at 298 K.

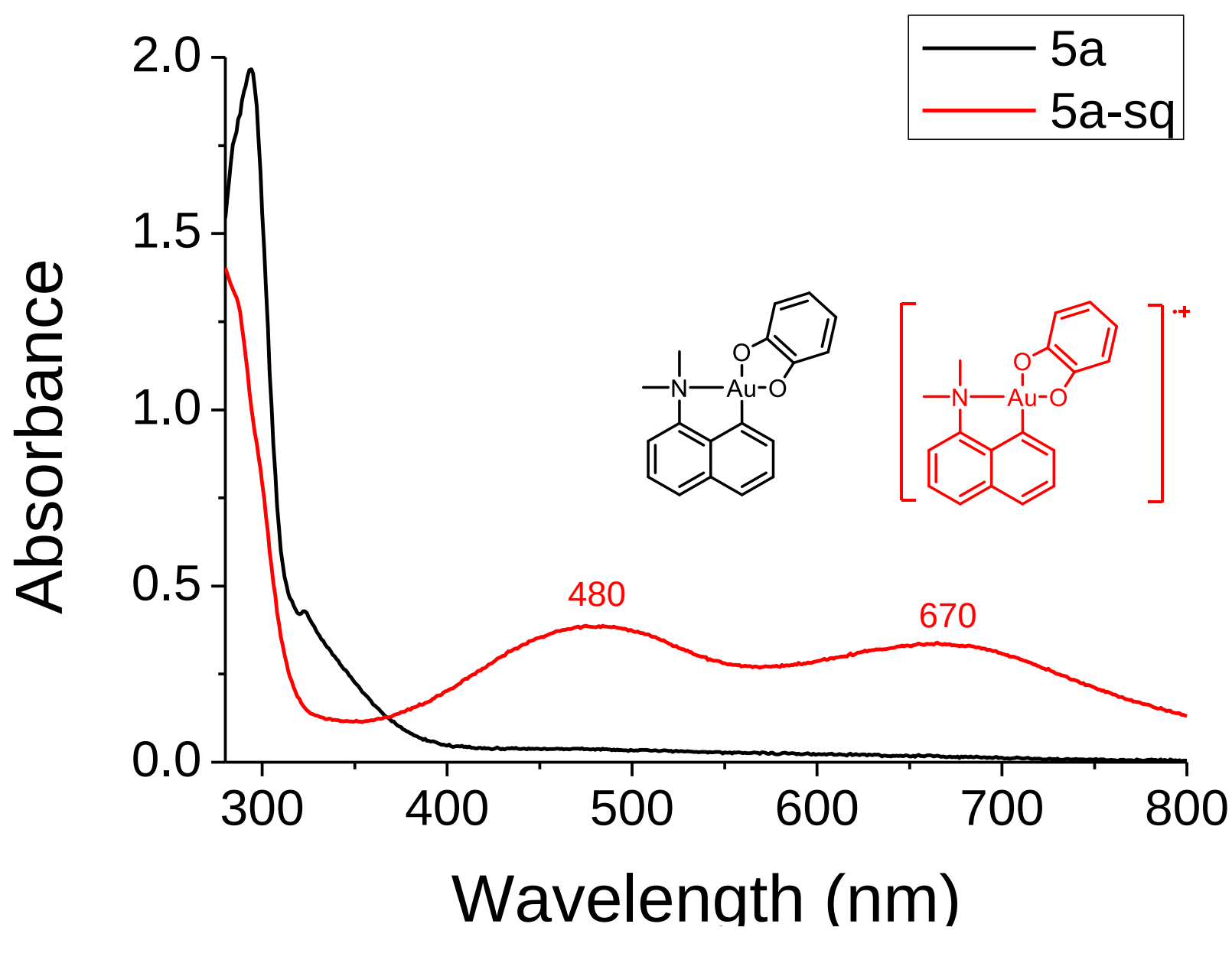

Figure S3. UV-Vis spectra of **5a** (black) and **5a-sq** (red) DCM solution (10^{-3} M) at 298 K. Over a period of 10 minutes, at room temperature, a disappearance of the band at 670 nm is observed and a precipitate starts to form.

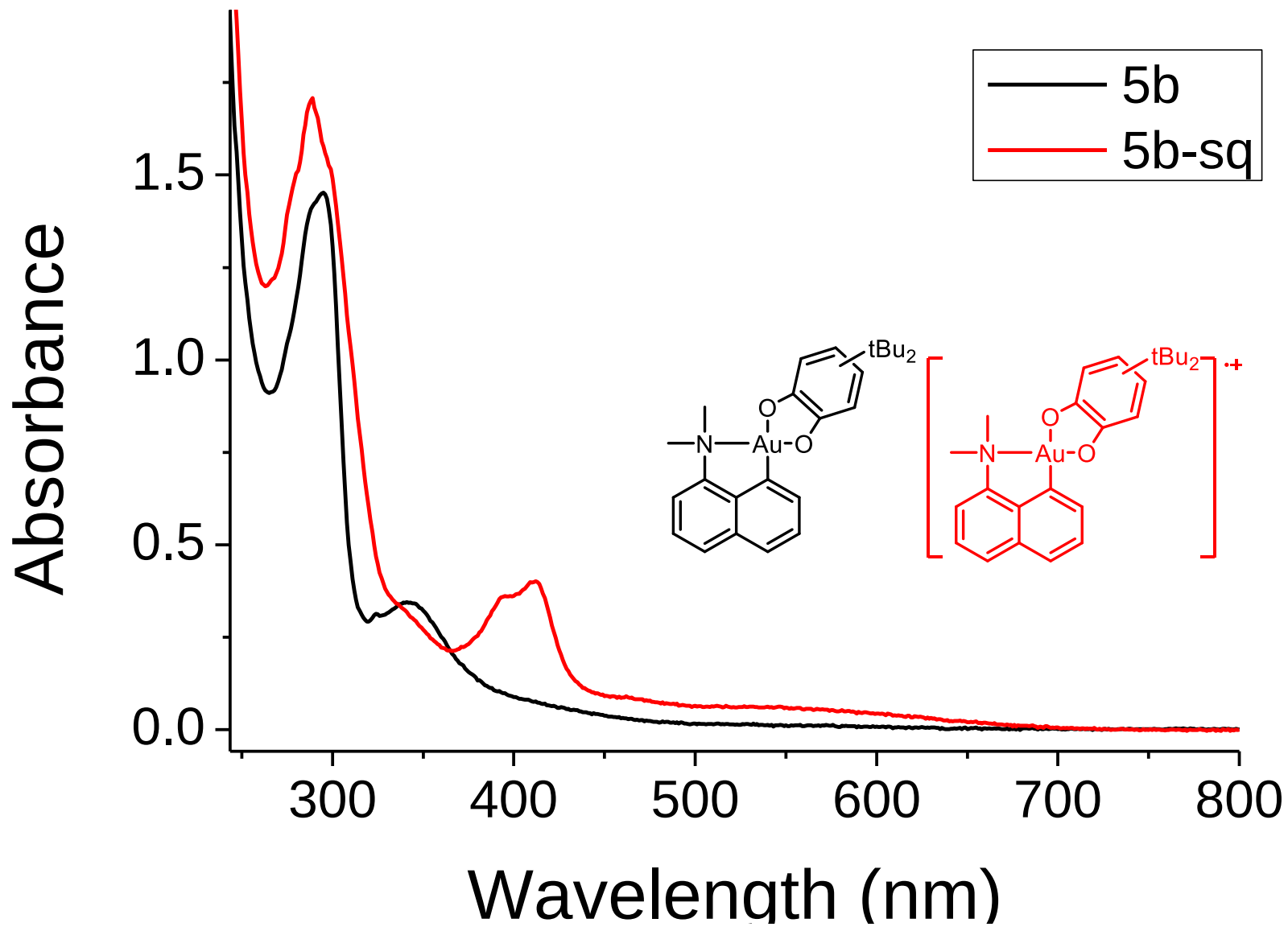

Figure S4. UV-Vis spectra of **5b** (black) and **5b-sq** (red) in DCM solution (10^{-3} M) at 298 K.

2.4 EPR Data

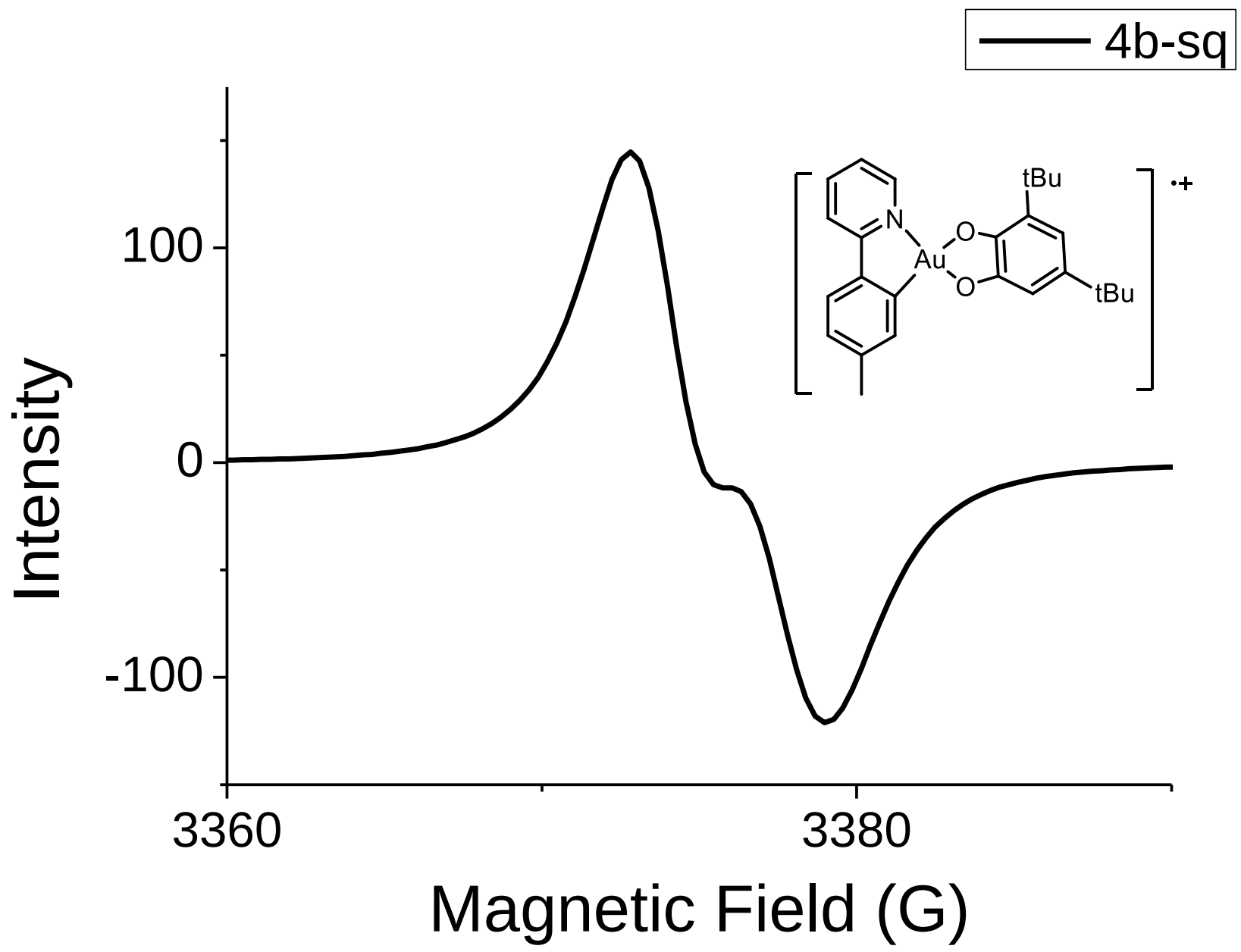

Figure S5. X-band (ν ≈ 9.45 GHz, Amplitude = 1.0 G) CW EPR spectra of the complex **4b-sq** prepared by oxidation with $AgBF_4$ in dry DCM, measured at 193 K. The isotropic g-value, g_{iso} = 1.9990.

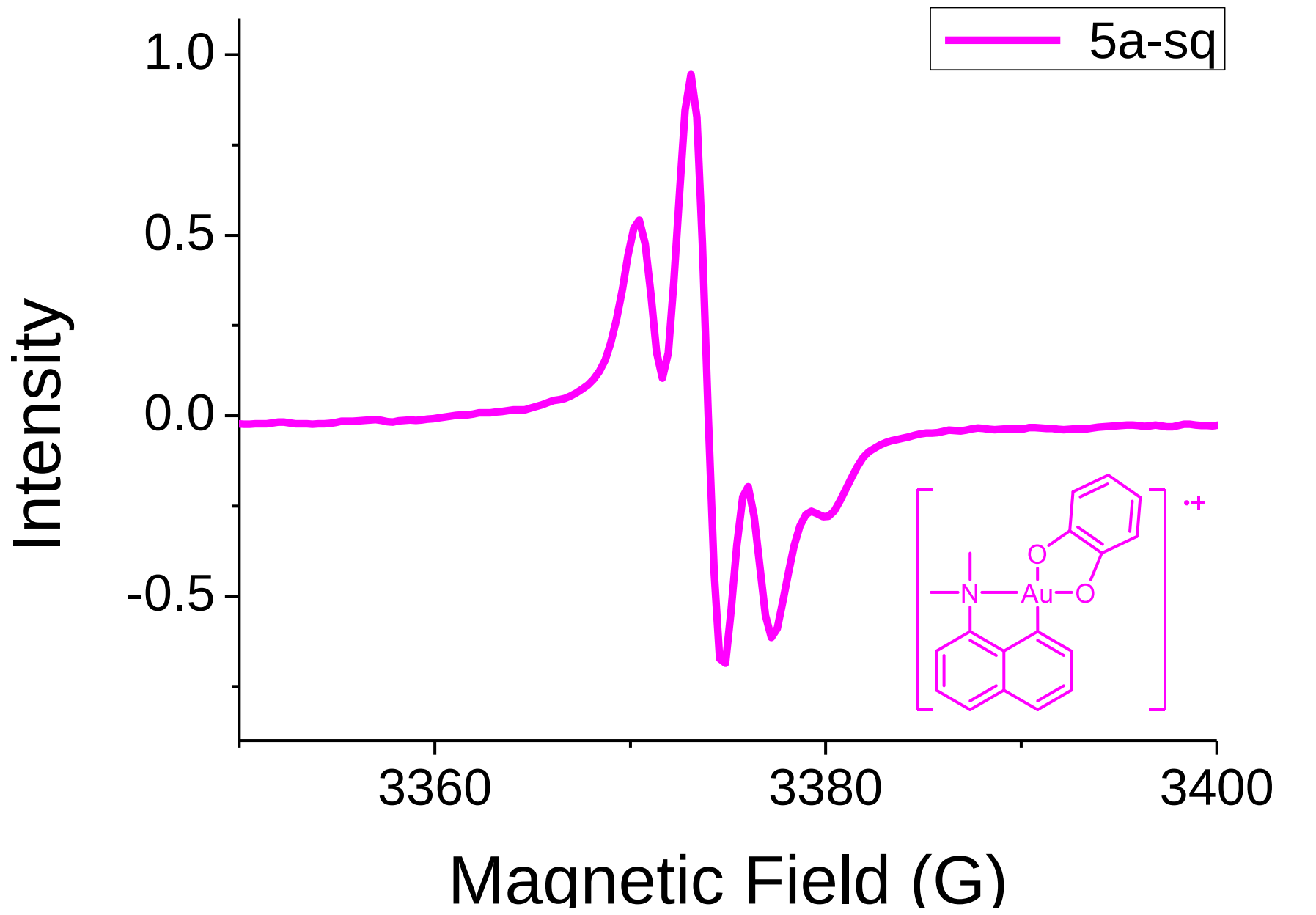

Figure S6. X-band (ν ≈ 9.45 GHz, Amplitude = 1.0 G) CW EPR spectra of the complex **5a-sq** prepared by oxidation with $AgBF_4$ in dry DCM, measured at 193 K. The isotropic g-value, g_{iso} = 1.9998.

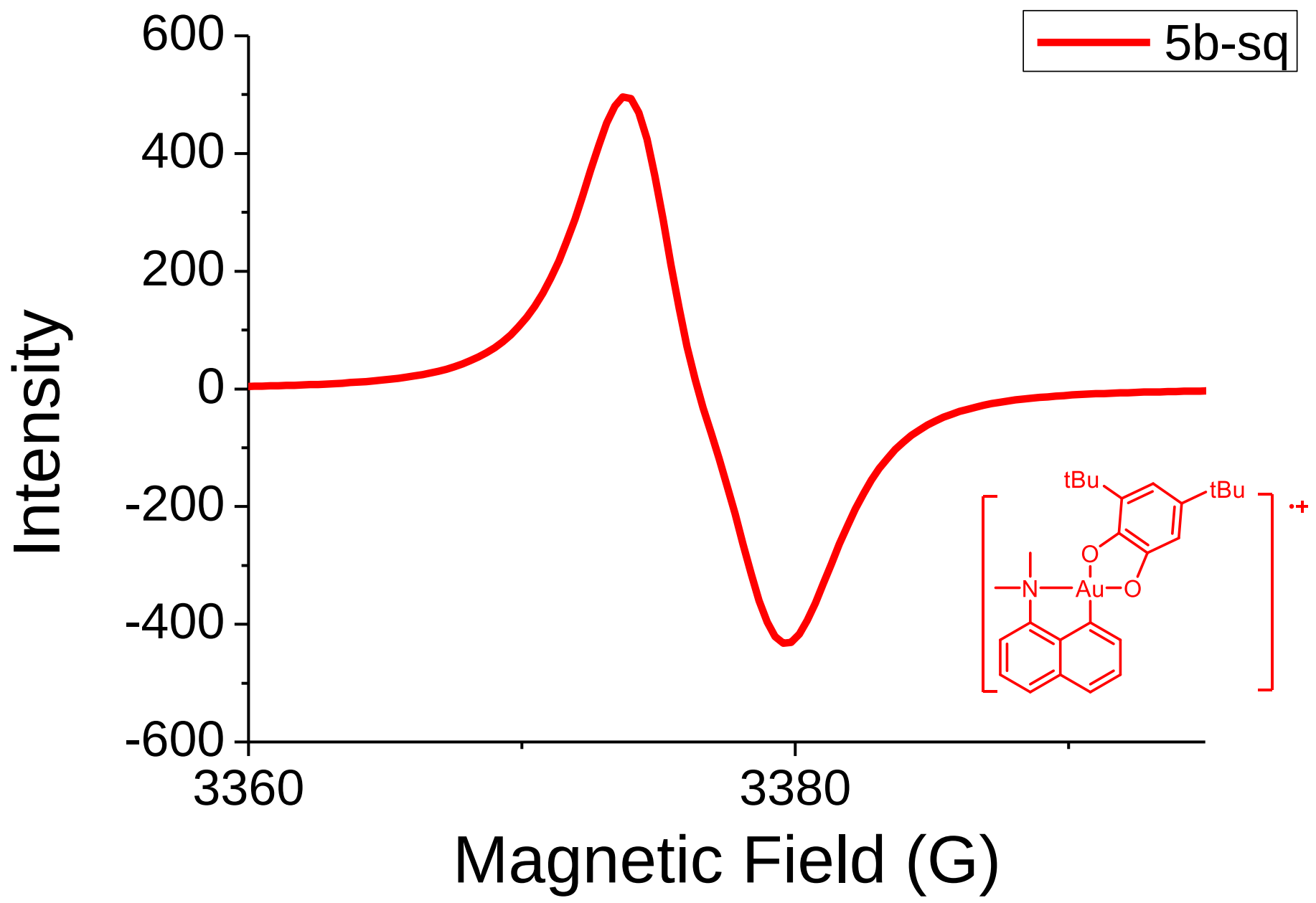

Figure S7. X-band (ν ≈ 9.45 GHz, Amplitude = 1.0 G) CW EPR spectra of the complex **5b-sq** prepared by oxidation with $AgBF_4$ in dry DCM, measured at 193 K. The isotropic g-value, g_{iso} = 1.9983.

2.5 Cyclic Voltammetry Data

As noted in the main text, the unsubstituted N^C gold(III) catecholate complexes **4a** and **5a** display electrochemically irreversible oxidation, in contrast to their *tert*-butyl-substituted analogues **4b** and **5b**. To understand the origin of this irreversibility, scan-rate dependent cyclic voltammograms were recorded for complex **4a** at a platinum working electrode over the range 10–500 mV s^{-1} (Figure S8a, top/bottom).

Complex **4a** and **5a** exhibit irreversible behaviour. Neither strictly anhydrous conditions nor substitution of the platinum working electrode for glassy carbon improved the reversibility of the process for both complexes. The scan-rate dependence for complex **5a** was examined: as shown in Figure S8A (top), the cathodic waves at high scan rates (> 200 mV s^{-1}) display larger peak currents compared to the non-existent waves at low scan rates (< 20 mV s^{-1}). As seen in Figure S8B (top), a clear asymmetry of i^{pa}/i^{pc} versus $v^{½}$ (Randles–Ševčík) can be observed across scan rates: this pattern is consistent with an EC mechanism, in which a rate-determining chemical step follows the initial electron transfer.[24] In contrast, for complex **5b** (Figure S8A, bottom), quasi-reversibility can be observed across multiple scan-rates, with a behaviour that follows symmetry of i^{pa}/i^{pc} versus $v^{½}$ (Figure S8B, bottom). Taken together, the contrasting electrochemical behaviours of **4a/5a** and **4b/5b** indicate that the *tert*-butyl substituents on the catecholate ring provide essential steric protection of the semiquinone intermediate, suppressing the follow-up chemistry that renders the unsubstituted complexes irreversible.

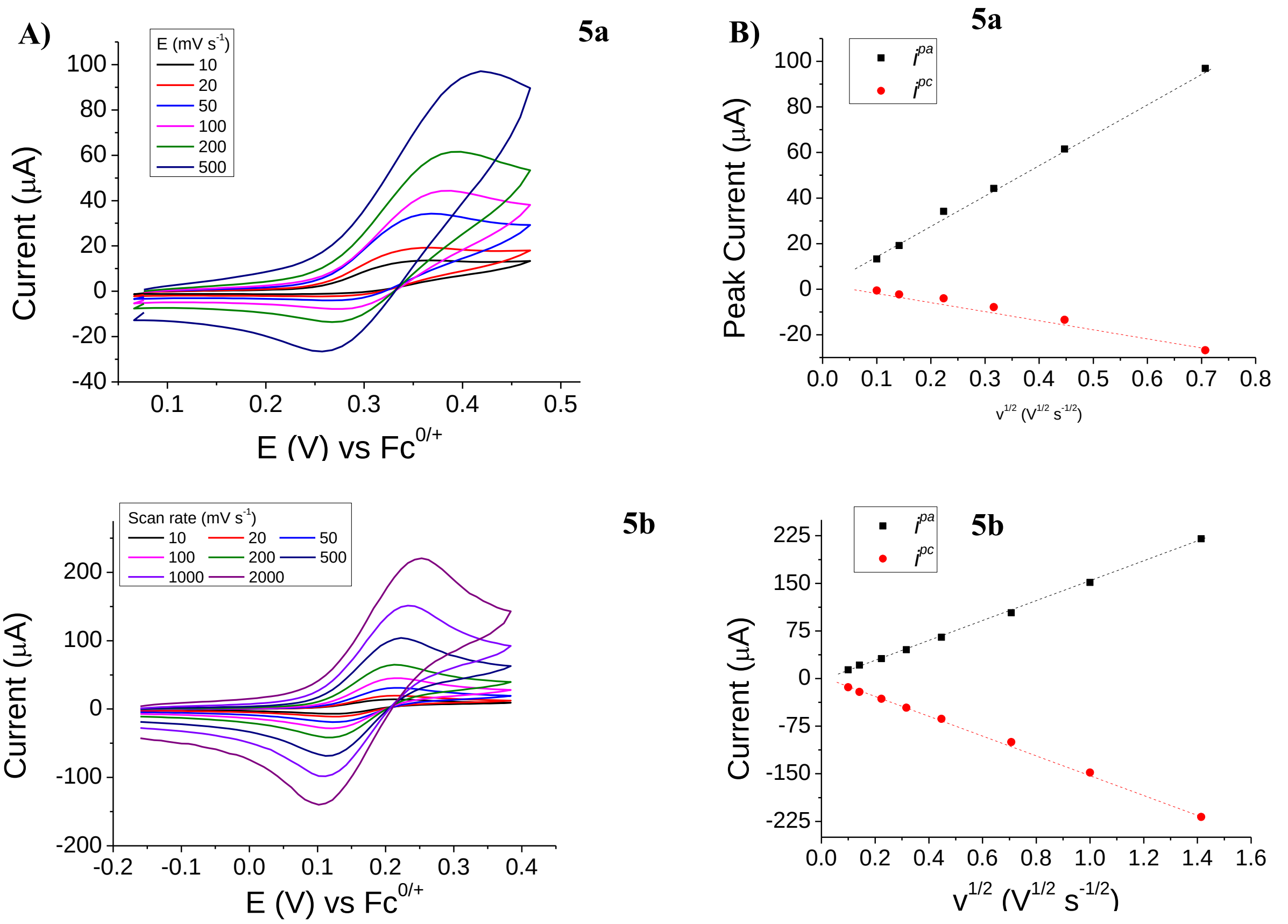

Figure S8. a) Scan-rate dependent cyclic voltammograms recorded at a platinum disk electrode from 500–10 mV s^{-1} for complex **5a** and from 2000–10 mV s^{-1} (0.25 mM in 0.25 M $[Bu_4N][PF_6]$ for complex **5b** in dry DCM). b) Anodic and cathodic current peaks (i^{pa} and i^{pc}) for versus square root of the scan rate ($v^{1/2}$): the lack of symmetry for complex **5a** is diagnostic of irreversible behaviour.

3. EDA Complexation with aryldiazoniums as acceptors

3.1 Job plot experiments (EDA stoichiometry)

The binding stoichiometry of the EDA complex in DMSO was determined by the method of continuous variations (Job's method)[25,26] through UV-Vis spectroscopic titration. Separate stock solutions of donor **5a** and the *p*-$BrPhN_2^+$ acceptor were prepared, and mixed in different molar ratios while keeping the total concentration constant (10^{-4} M). UV–Vis spectra were acquired following a brief equilibration period of 1–2 minutes under dark conditions to minimize photodecomposition. The change in absorbance at 290 nm, a wavelength characteristic of donor–acceptor complex formation, was then plotted as a function of donor mole fraction. In each case, a distinct maximum was observed at a mole fraction of approximately 0.5, which is diagnostic of a 1:1 donor-to-acceptor binding stoichiometry.

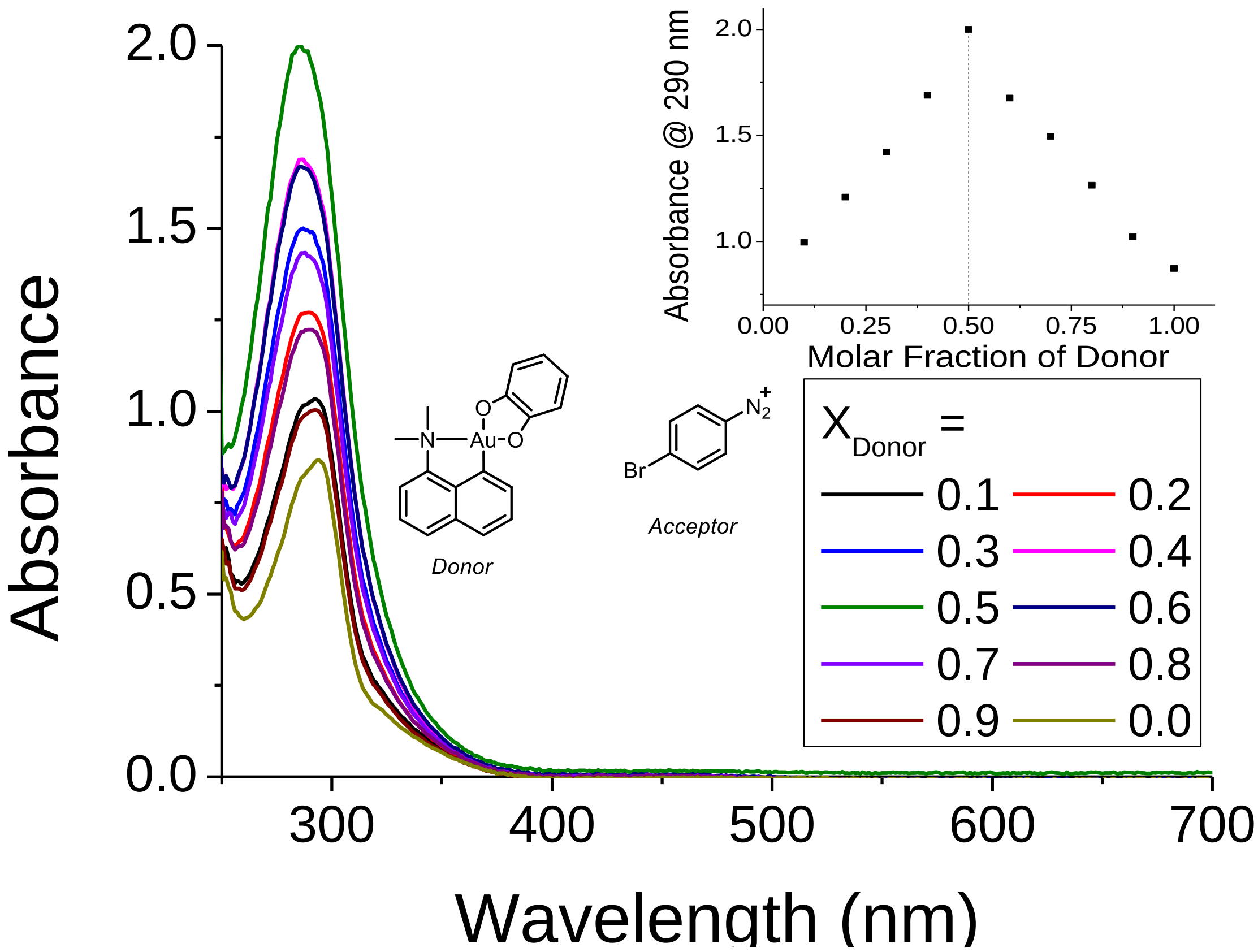

Figure S9. Job's plot for the **5a**/*p*-$BrPhN_2^+$ EDA complex in DMSO ([total] = 10^{-4} M). The inset shows a maximum at $\chi_{donor} \approx 0.5$ using $\lambda = 290$ nm, indicative of a 1:1 stoichiometry.

4. C–H arylation reactions of furan

General procedure for the catalysis with diazonium salts:

In an NMR tube, the corresponding diazonium salt **6** (0.1 mmol) and the respective gold complex **4a,b**/**5a,b** (0.001 mmol, 0.01 equiv or 1 mol %) were weighed. Then, 0.5 mL of DMSO d_6 was added and the NMR tubes were degassed by three freeze-thaw sequences and filled with Ar; following this furan (0.5 mmol, 5 equiv) was injected into the mixture. Then, the tubes with all samples were place facing a green LED (5 W, 530 nm) and parallel to each other inside a refrigerated bath at 20 °C. Periodically, the reaction conversion was monitored by ^{1}H NMR, and once full conversion was reached, the internal standard (1,3,5-trimethoxybenzene) was added to the mixture. The yield was then determined by ^{1}H NMR spectroscopy. A comparison with a benchmark organic dye Eosin Y, which has previously been reported for this reaction,[27] was made under the same conditions.

Different diazonium salts were tested:

- *p*-(methyl)phenyldiazonium salt **6.1**

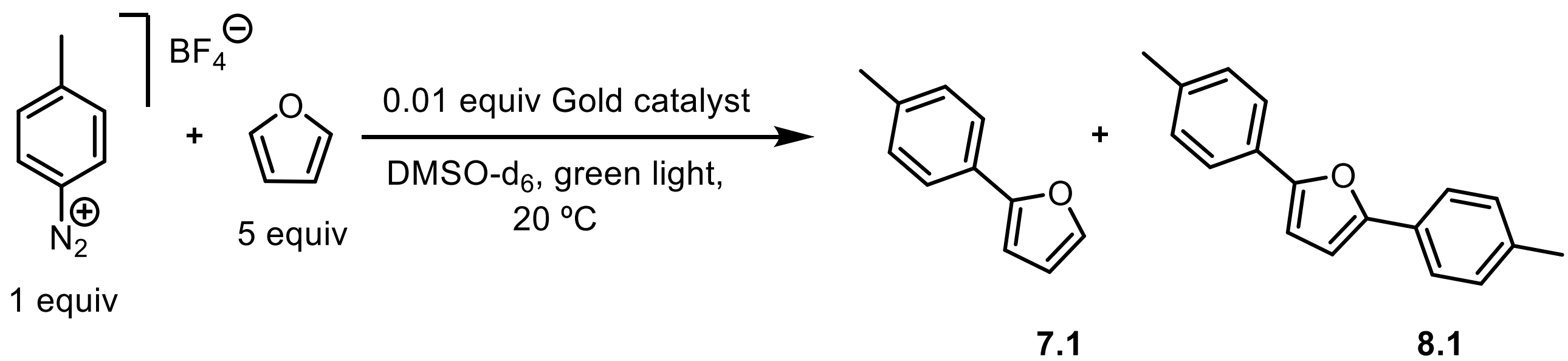

Table S1. *Yields of the reaction of p-(methyl)phenyldiazonium salt.*

Catalyst	Conversion	Yield 7.1 (%)	Yield 8.1 (%)
**Eosin Y**	100	52 (10 h)	19
**P^C-H₄**	100	30 (14 h)	Not determined[a]
**4a**	100	24 (18 h)	Not determined[a]
**4b**	100	46 (14 h)	Not determined[a]
**5a**	100	21 (18 h)	Not determined[a]
**5b**	100	48 (14 h)	Not determined[a]

[a] The signals of **8.1** are masked and/or overlapped with those of other side-products/impurities, preventing reliable quantification.

- *p*-(methoxy)phenyldiazonium salt **6.2**

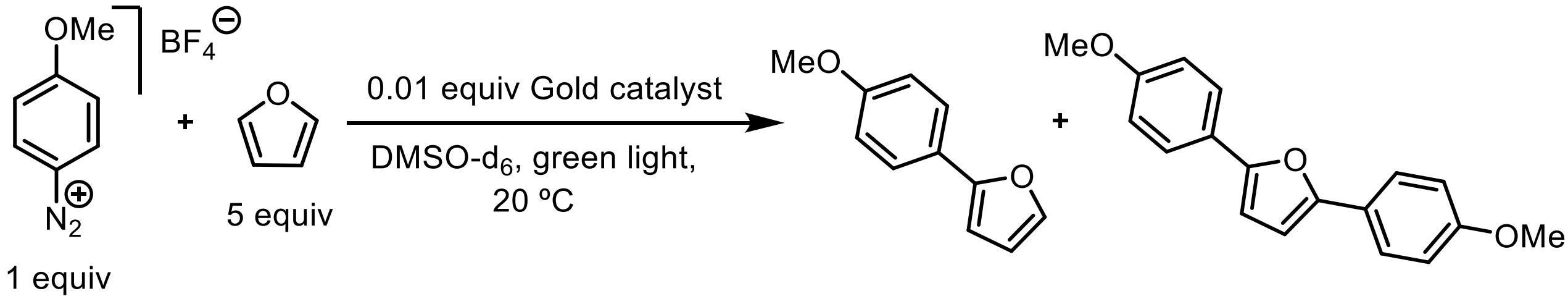

Table S2. *Yields of the reaction of p-(methoxy)phenyldiazonium salt.*

Catalyst	Conversion	Yield 7.2 (%)	Yield 8.2 (%)
**Eosin Y**	100	66 (10 h)	10
**P^C-H₄**	100	65 (14 h)	9
**4a**	100	27 (24 h)	Not determined[a]
**4b**	100	54 (14 h)	11
**5a**	100	23 (18 h)	Not determined[a]
**5b**	100	60 (14 h)	9

[a] The signals of **8.2** are masked and/or overlapped with those of other side-products/impurities, preventing reliable quantification.

Example of better performance with EDGs

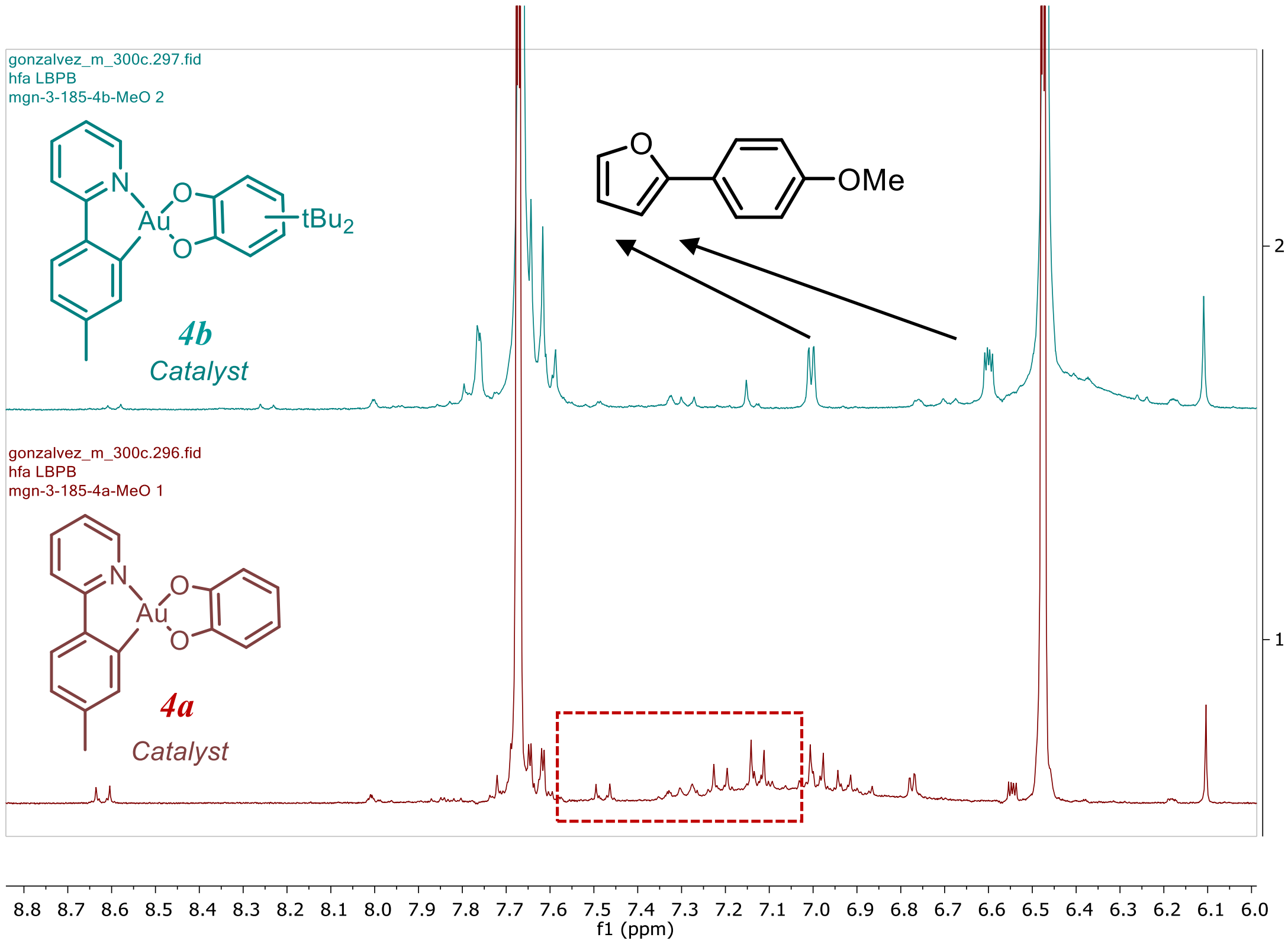

Figure S10. Reaction crudes for the reaction of p-MeOPhN$_2^+$ with furan at t = 14 h: top is **4b** and bottom is **4a**. Notice the impurities present in the **4a** sample between 7.3-6.9 ppm.

- phenyldiazonium salt **6.3**

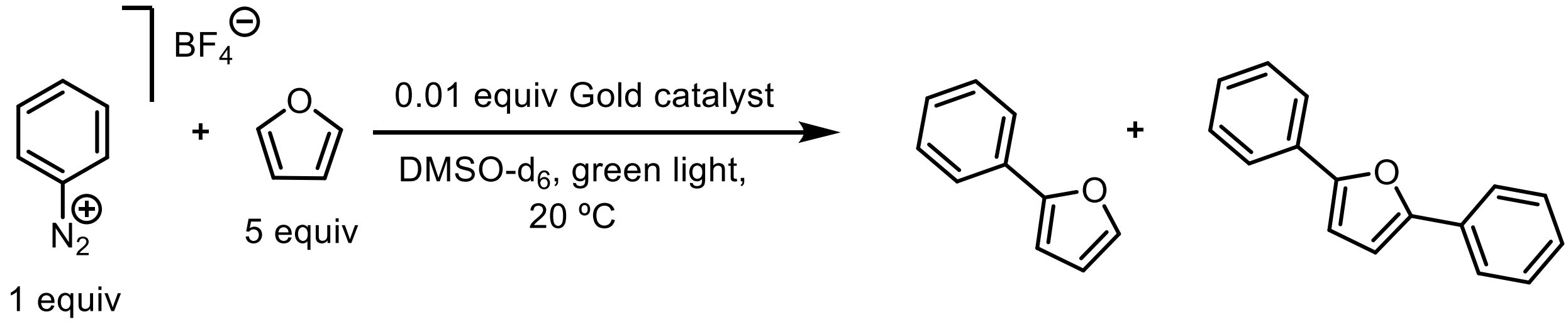

7.3 **8.3**

Table S3. *Yields of the reaction of phenyldiazonium salt.*

Catalyst	Conversion	Yield 7.3 (%)	Yield 8.3 (%)
**Eosin Y**	100	32 (6 h)	--
**P^C-H$_4$**	100	25 (8 h)	--
**4a**	100	20 (18 h)	2
**4b**	100	31 (18 h)	5
**5a**	100	14 (18 h)	6
**5b**	100	23 (18 h)	2

- *p*-(fluoro)phenyldiazonium salt **6.4**

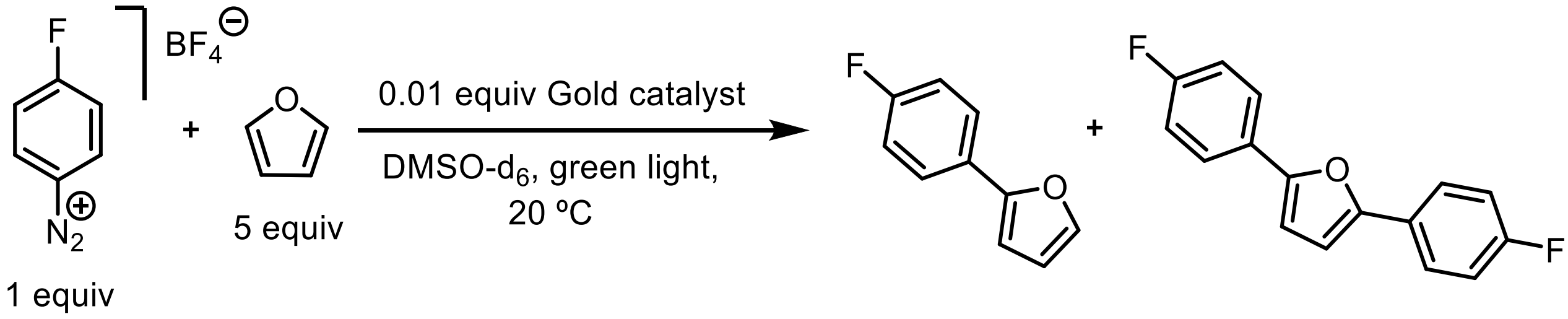

7.4 **8.4**

Table S4. *Yields of the reaction of p-(fluoro)phenyldiazonium salt.*

Catalyst	Conversion	Yield 7.4 (%)	Yield 8.4 (%)
**Eosin Y**	100	78 (1 h)	11
**P^C-H$_4$**	100	79 (2 h)	11
**4a**	100	38 (12 h)	8
**4b**	100	49 (12 h)	10
**5a**	100	40 (6 h)	8
**5b**	100	52 (12 h)	9

- *p*-(bromo)phenyldiazonium salt **6.5**

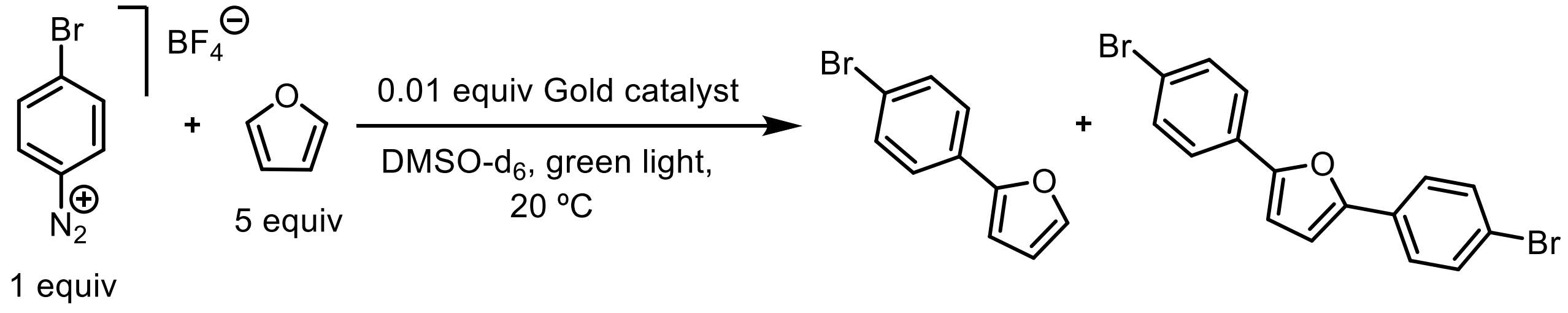

Table S5. *Yields of the reaction of p-(bromo)phenyldiazonium salt.*

Catalyst	Conversion	Yield 7.5 (%)	Yield 8.5 (%)
**Eosin Y**	100	79 (2 h)	9
**P^C-H$_4$**	100	81 (3 h)	12
**4a**	100	54 (6 h)	8
**4b**	100	70 (6 h)	10
**5a**	100	67 (6 h)	8
**5b**	100	73 (6 h)	11

- *p*-(nitro)phenyldiazonium salt **6.6**

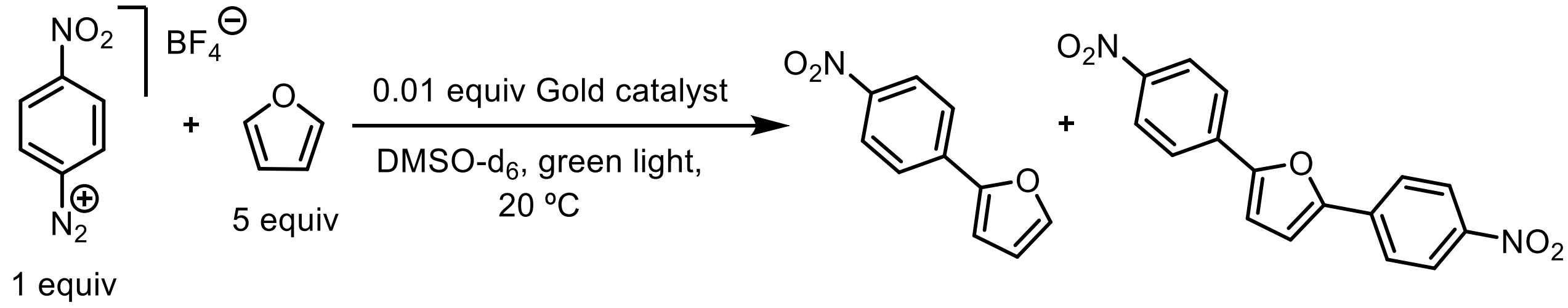

Table S6. *Yields of the reaction of p-(nitro)phenyldiazonium salt.*

Catalyst	Conversion	Yield 7.6 (%)	Yield 8.6 (%)
**Eosin Y**	100	84 (20 min)	10
**P^C-H$_4$**	100	82 (20 min)	10
**4a**	100	71 (1 h)	6
**4b**	100	67 (1 h)	7
**5a**	100	58 (1 h)	8
**5b**	100	54 (1 h)	7

5. Computational Studies

Table S7. Selected geometrical parameters, including bond distances in angstroms (Å) and bond angles in degrees (°), for complexes **P^C-H$_4$**, **4b**, **5a**, **5b** and their oxidized radical cationic species (**P^C-H$_4$-sq**, **4b-sq**, **5a-sq**, **5b-sq**) optimized at SMD(DCM)-PBE0-D3(BJ)/SDD+f(Au), 6-31G** (other atoms) level of theory. Löwdin spin population analysis (ρ_S) of the Au center and the semiquinone ligand (O^O) for the radical cationic species computed at SMD(DCM)-ZORA-PBE0-D4/SARC-ZORA-TZVP (Au), ZORA-def2-TZVP (other atoms).

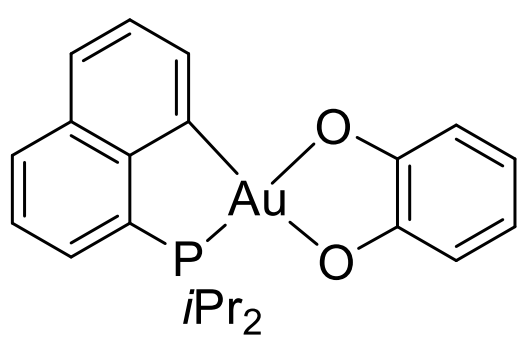

P^C-H$_4$

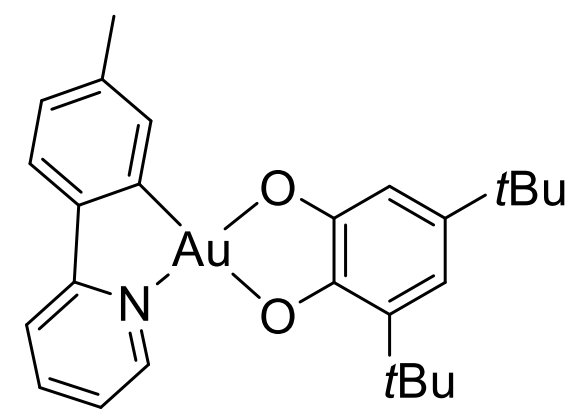

4b

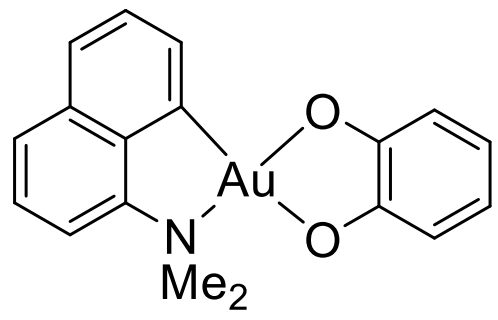

5a

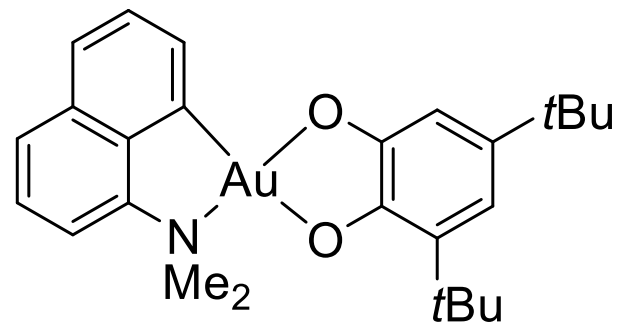

5b

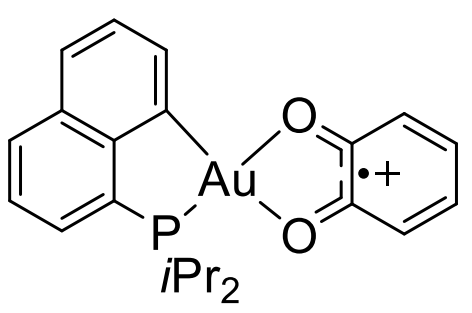

P^C-H$_4$-sq

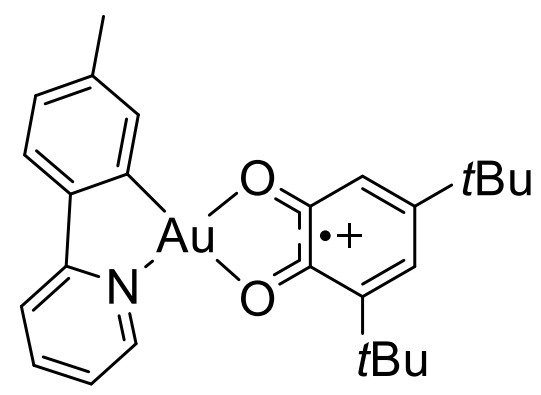

4b-sq

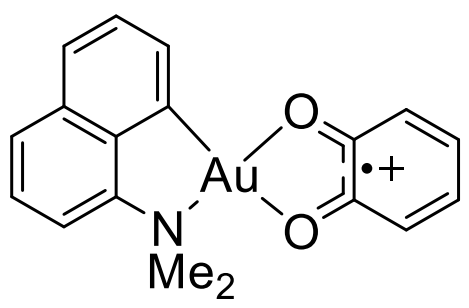

5a-sq

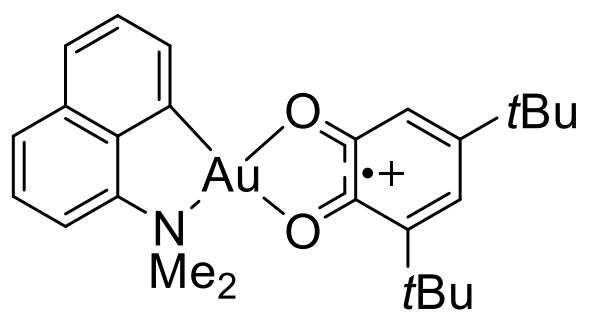

5b-sq

Numbering

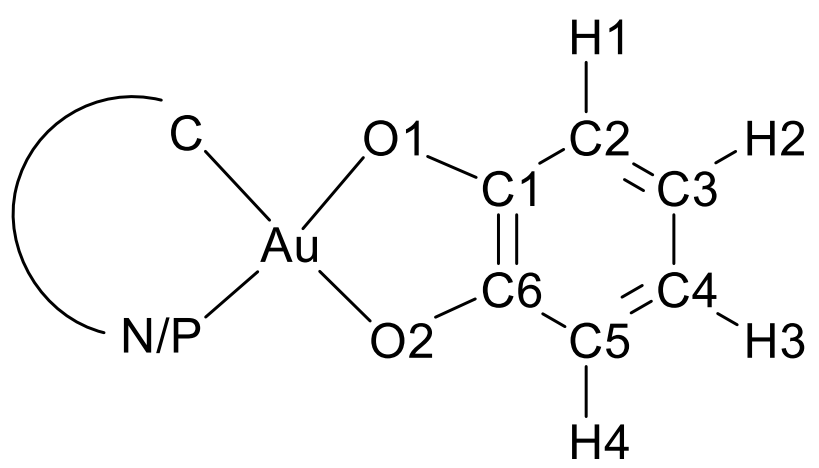

Parameters	P^C-H$_4$	P^C-H$_4$-sq	4b	4b-sq	5a	5a-sq	5b	5b-sq
**Au–P/N**	2.284	2.285	2.014	2.004	2.082	2.068	2.083	2.069
**Au–C**	2.007	2.001	1.992	1.986	1.985	1.978	1.985	1.977
**Au–O1**	2.039	2.098	1.965	1.999	1.974	2.013	1.969	2.002
**Au–O2**	2.057	2.111	2.038	2.106	2.049	2.123	2.041	2.111
**O1–Au–O2**	82.78	79.40	83.98	80.72	84.48	81.04	84.16	80.90
**C1–C2**	1.394	1.414	1.387	1.399	1.391	1.408	1.387	1.393
**C2–C3**	1.398	1.372	1.401	1.380	1.398	1.374	1.401	1.386
**C3–C4**	1.392	1.427	1.396	1.436	1.392	1.427	1.396	1.430
**C4–C5**	1.398	1.371	1.404	1.373	1.397	1.370	1.405	1.375
**C5–C6**	1.395	1.415	1.406	1.434	1.395	1.418	1.406	1.434
**C6–C1**	1.416	1.459	1.410	1.452	1.413	1.456	1.410	1.455
**O1–C1**	1.349	1.295	1.362	1.313	1.361	1.307	1.363	1.315
**O2–C6**	1.346	1.293	1.351	1.294	1.344	1.291	1.351	1.293
**ρ_S(Au)**	-	0.012	-	0.012	-	0.012	-	0.011
**ρ_S(O^O)**	-	0.988	-	0.985	-	0.989	-	0.986
**MOS**	-1.87(5)	-1.07(4)	-1.94(9)	-1.14(7)	-1.92(5)	-1.10(5)	-1.94(9)	-1.17(7)

Geometry variations are minor and mainly associated with the semiquinone **(O^O)** ligand. A decrease in the Au–O bond lengths is observed when going from P^C to N^C ligands. The Au–O1 distances are more significantly affected than the Au–O2 distances because of the *trans* effect of the nitrogen, resulting in more dissymmetrical Au(O^O) metallacycle in complexes featuring N^C ligands. These observations do not extend to the C–O bonds of the metallacycle, which remain relatively equivalent. Extending the analysis to the aromatic ring of the **(O^O)** ligand shows no effect of the (N^C) ligands on the geometrical features compared to the P^C ligand.

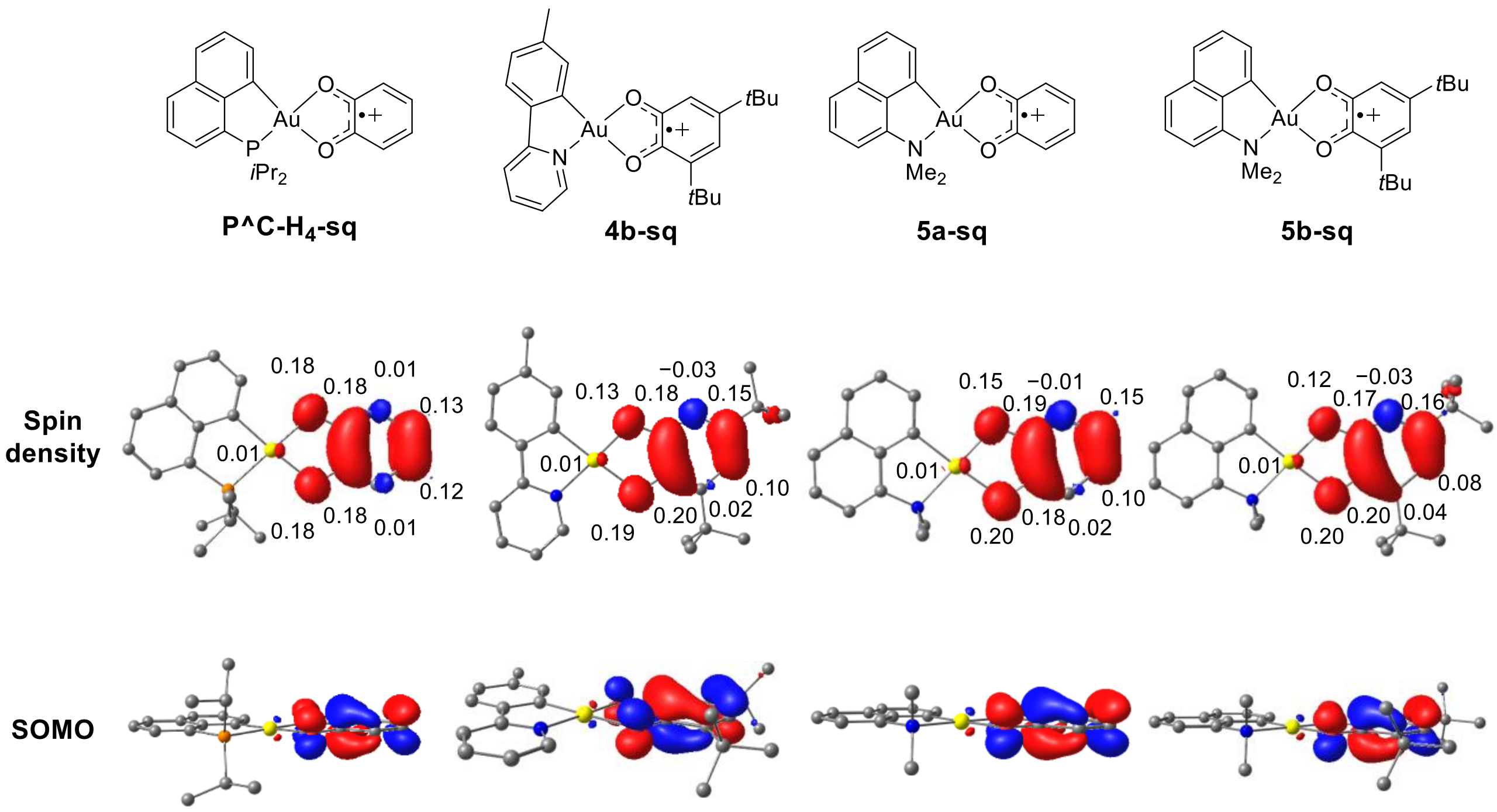

Figure S11. Plot of the spin density distribution (cutoff: 0.002 a.u.); in red: α spin distribution, in blue: β spin polarization for the radical cationic complexes **P^C-H$_4$-sq**, **4b-sq**, **5a-sq** and **5b-sq**. Löwdin spin population values shown for Au and the non-hydrogen atoms of the semiquinone ligand's π system. Plot of the Semi Occupied Molecular Orbital (**SOMO**, cutoff: 0.05 a.u.). Calculations carried out at SMD(DCM)-ZORA-PBE0-D4/SARC-ZORA-TZVP(Au), ZORA-def2-TZVP (other atoms)//SMD(DCM)-PBE0-D3(BJ)/ SDD+f(Au), 6-31G** (other atoms) level of theory.

The spin density plots and SOMO representations for all complexes show that the unpaired electron is predominantly localized on the π-system of the catecholate moiety, with only a negligible contribution from the Au center.

The spin density and SOMO plots, despite the *trans* effect, reveal a π-system that remains highly symmetric overall. However, a more detailed analysis of the spin density values shows a slight asymmetric localization of the unpaired electron on the π-system of the semiquinone ligand in complexes featuring N^C ligands.

Table S8. Ionization Potential (**IP** in kcal·mol^{-1}) and main EPR parameters, including $\mathbf{g_{iso}}$ and hyperfine coupling constant $\mathbf{A_H}$ (in MHz,) for the radical cations of interest: **P^C-H$_4$-sq**, **4b-sq**, **5a-sq**, and **5b-sq**. Calculations carried out at SMD(DCM)-ZORA-PBE0-D4/SARC-ZORA-TZVP (Au), ZORA-def2-TZVP (other atoms) on the geometries optimized at SMD(DCM)-PBE0-D3(BJ)/SDD+f(Au), 6-31G** (other atoms).

Complexes	IP[a] [kcal·mol^{-1}]	$\mathbf{g_{iso}}$	$\mathbf{A_{H\text{-}ortho}}$ [MHz]	$\mathbf{A_{H\text{-}meta}}$ [MHz]
**P^C-H$_4$-sq**	102.6	1.9941	1.0 (H_1), 0.3 (H_4)	11.4 (H_2), 10.5 (H_3)
**4b-sq**	102.3	2.0005	4.5 (H_1)	7.9 (H_3)
**5a-sq**	105.6	1.9992	2.9 (H_1), 1.1 (H_4)	13.9 (H_2), 8.6 (H_3)
**5b-sq**	101.5	1.9990	5.1 (H_1)	6.3 (H_3)

[a] Ionization potentials of the parent complexes leading to the oxidized radical cations.

6. NMR Spectra

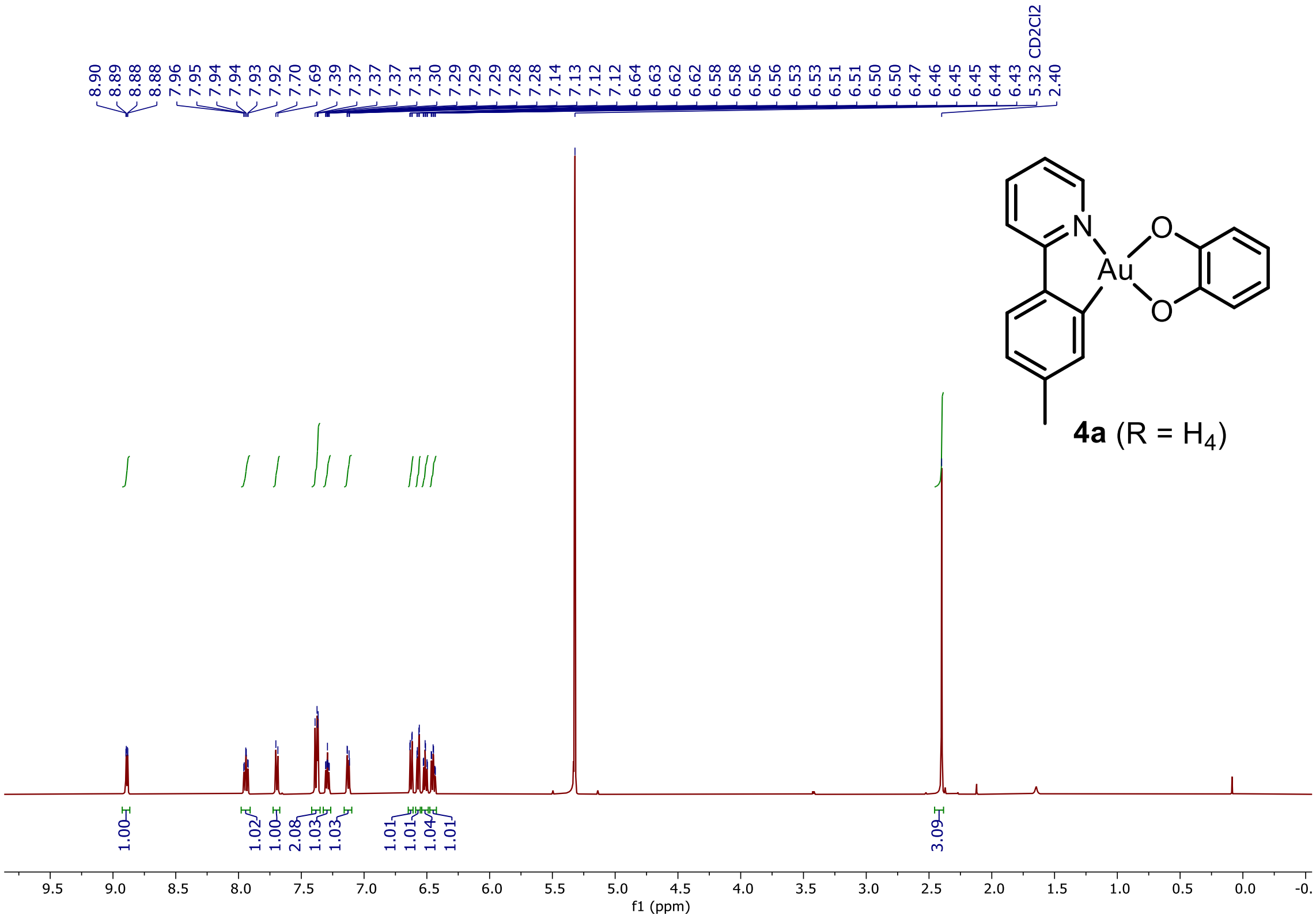

Figure S12. ^{1}H NMR Spectrum (500 MHz, 298 K, CD_2Cl_2) of gold complex **4a**.

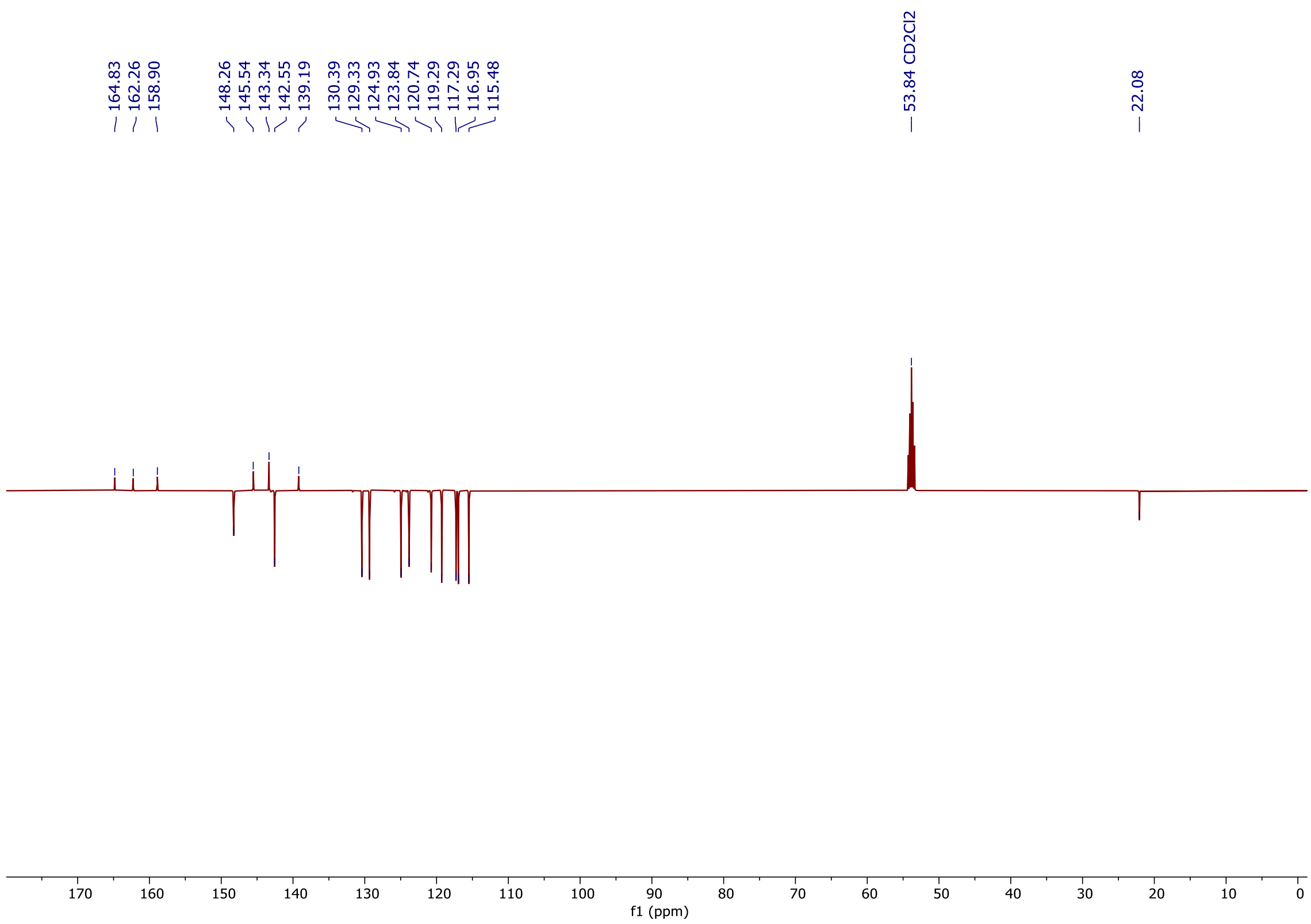

Figure S13. $^{13}C\{1H\}$ JMOD NMR Spectrum (126 MHz, 298 K, CD_2Cl_2) of gold complex **4a**.

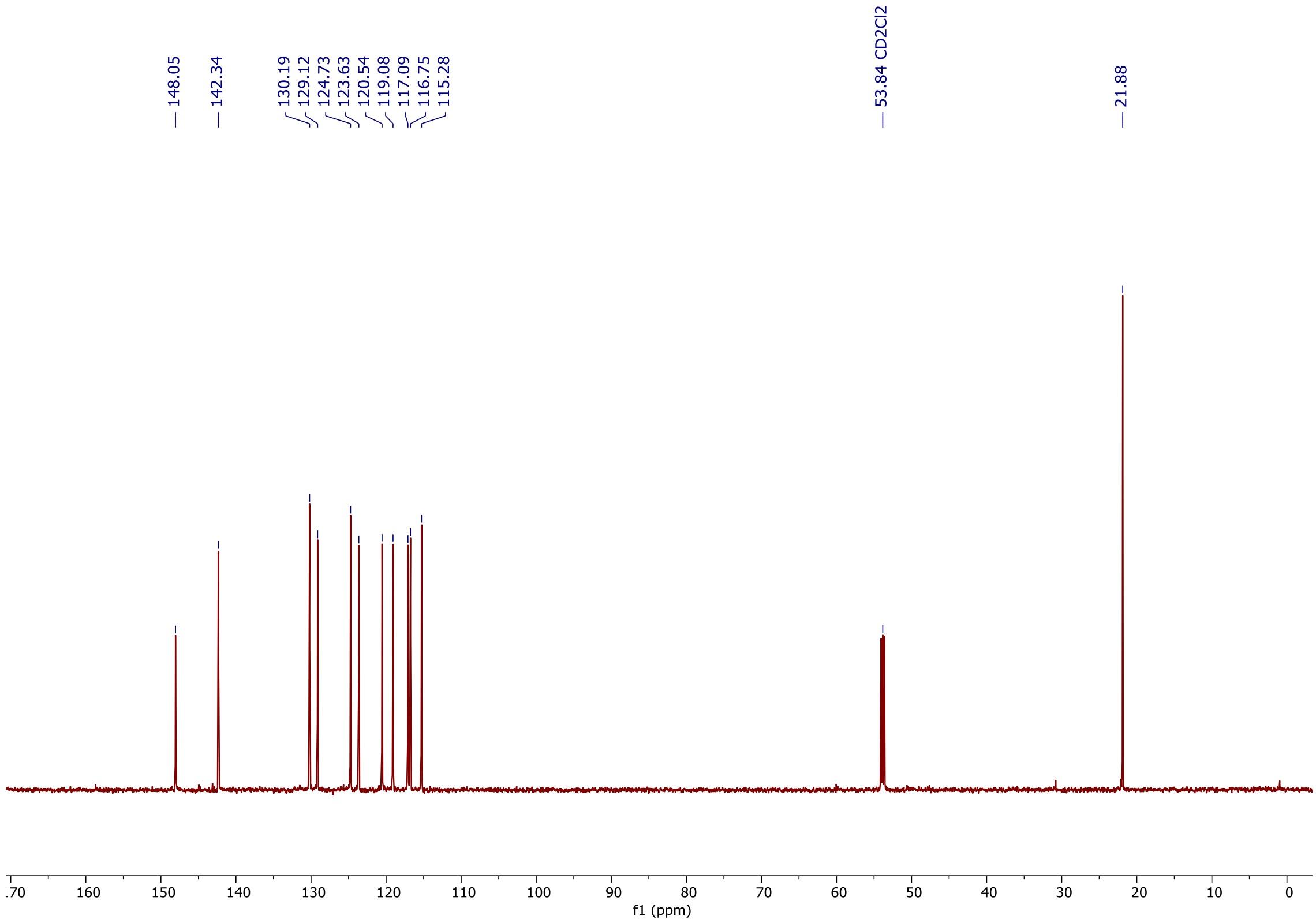

Figure S14. $^{13}C\{1H\}$ NMR Spectrum (126 MHz, 298 K, CD_2Cl_2) of gold complex **4a**.

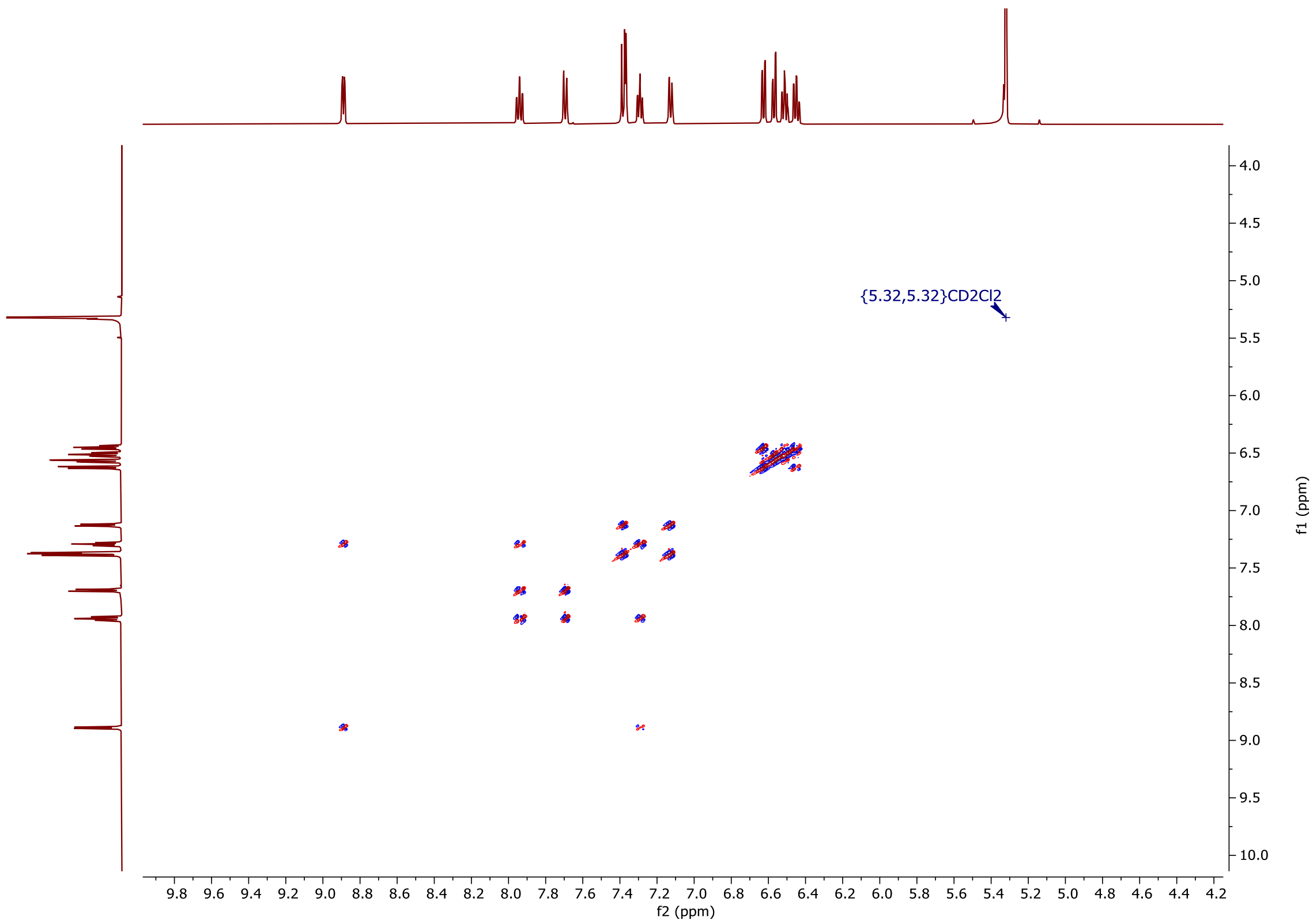

Figure S15. COSY NMR Spectrum (500 MHz, 298 K, CD_2Cl_2) of gold complex **4a**.

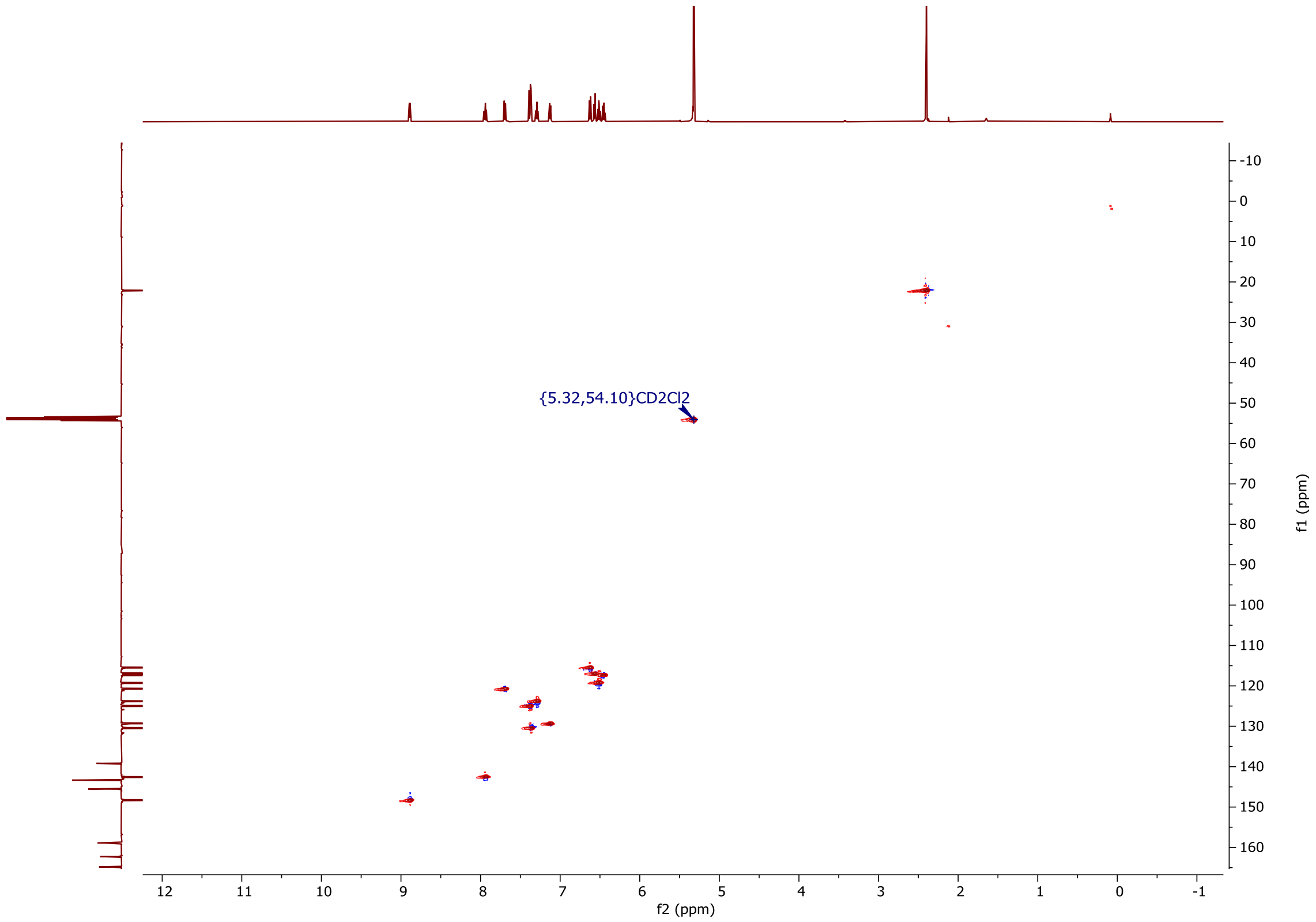

Figure S16. HSQC NMR Spectrum (500, 126 MHz, 298 K, CD_2Cl_2) of gold complex **4a**.

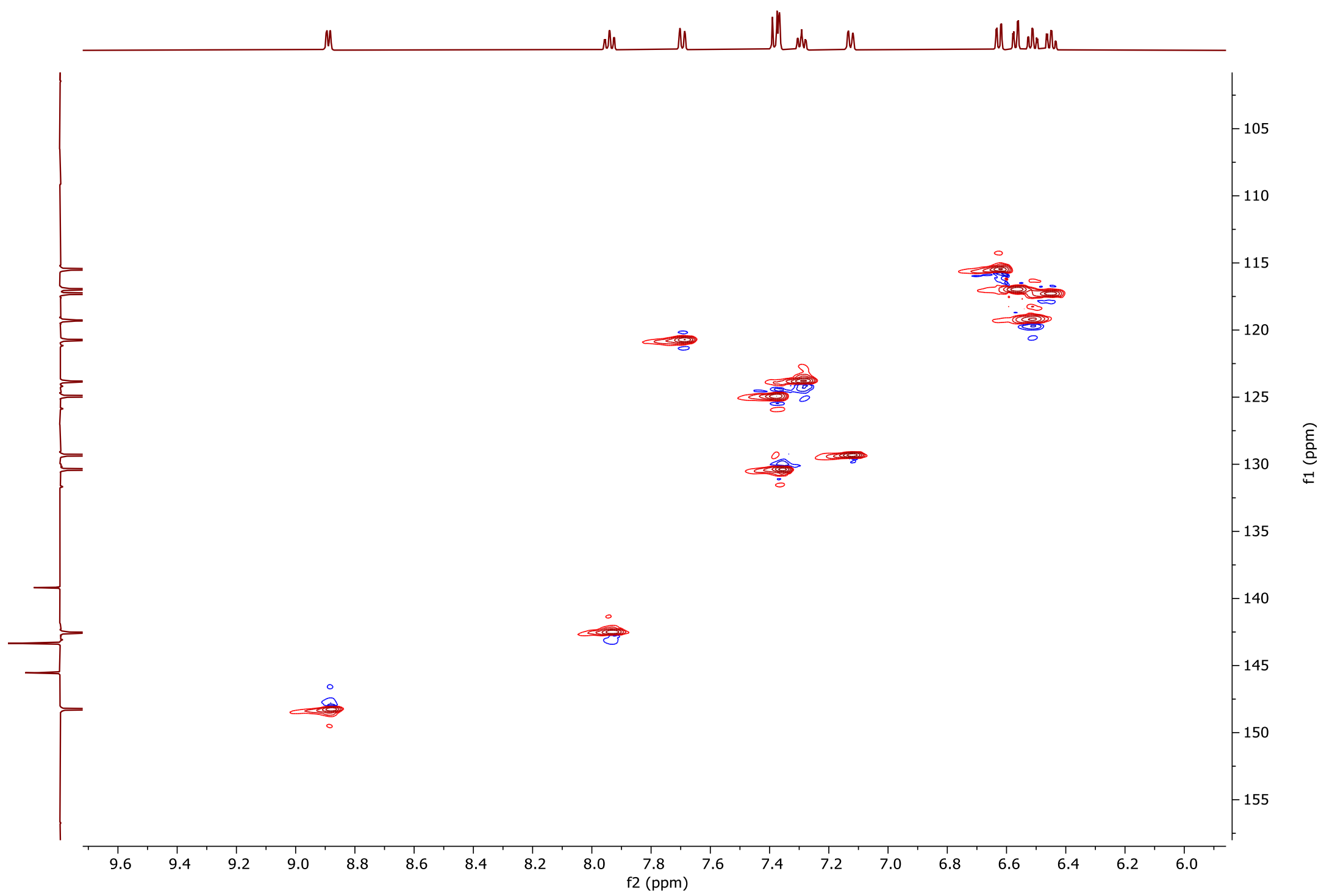

Figure S17. HSQC NMR Spectrum (500, 126 MHz, 298 K, CD_2Cl_2); close-up of aromatic signals of gold complex **4a**.

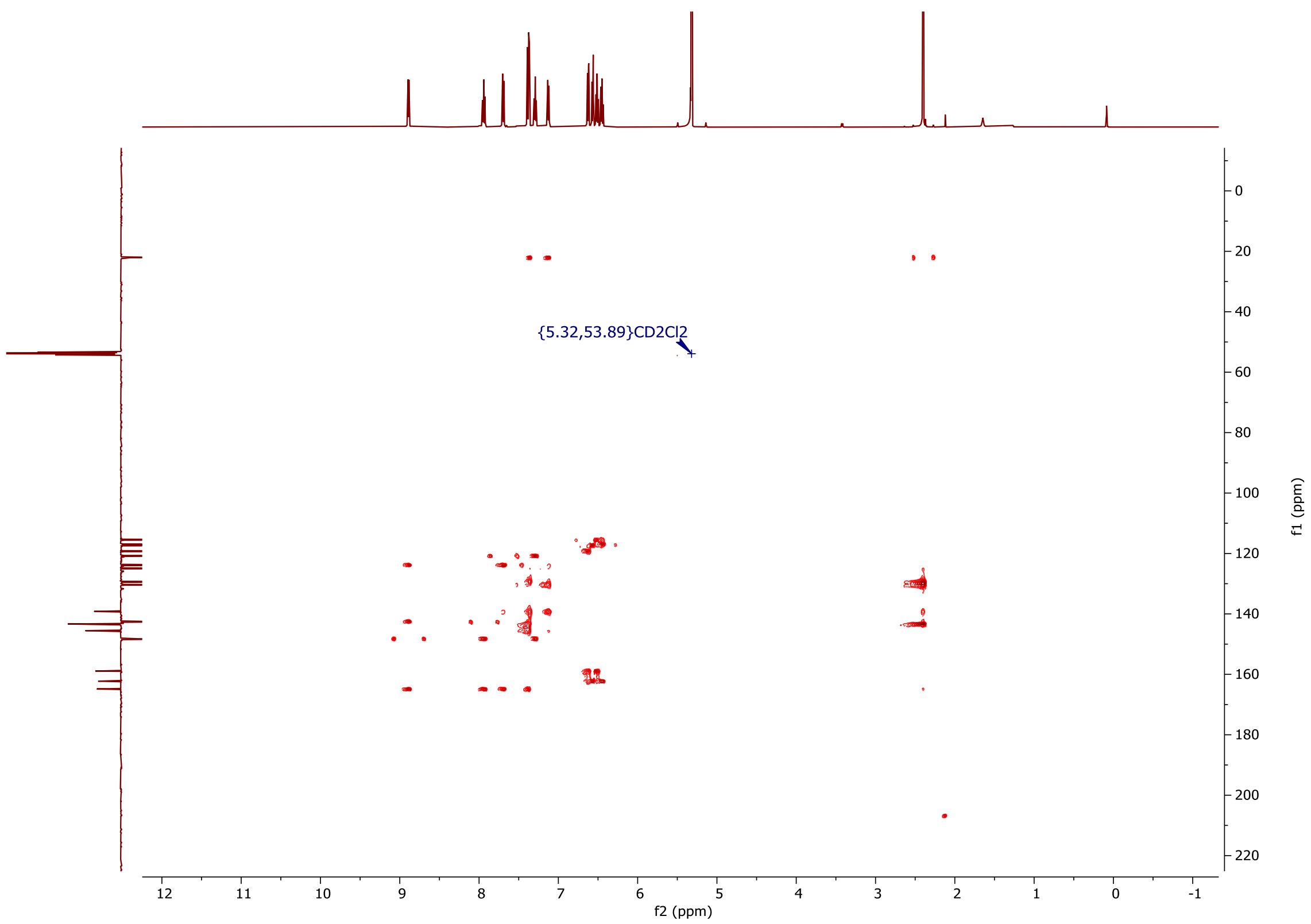

Figure S18. HMBC NMR Spectrum (500, 126 MHz, 298 K, CD_2Cl_2) of gold complex **4a**.

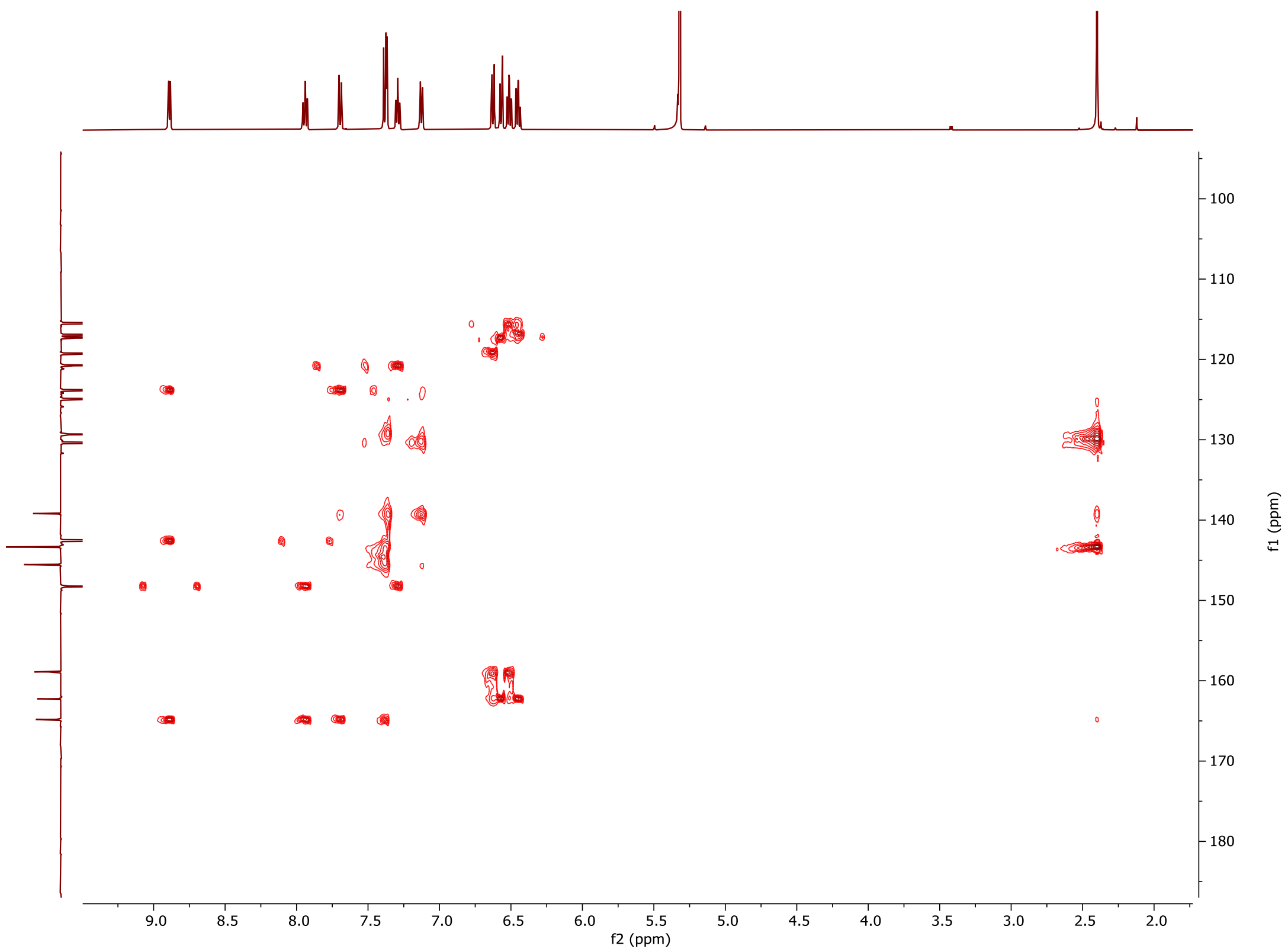

Figure S19. HMBC NMR Spectrum (500, 126 MHz, 298 K, CD_2Cl_2); close-up of aromatic signals of gold complex **4a**.

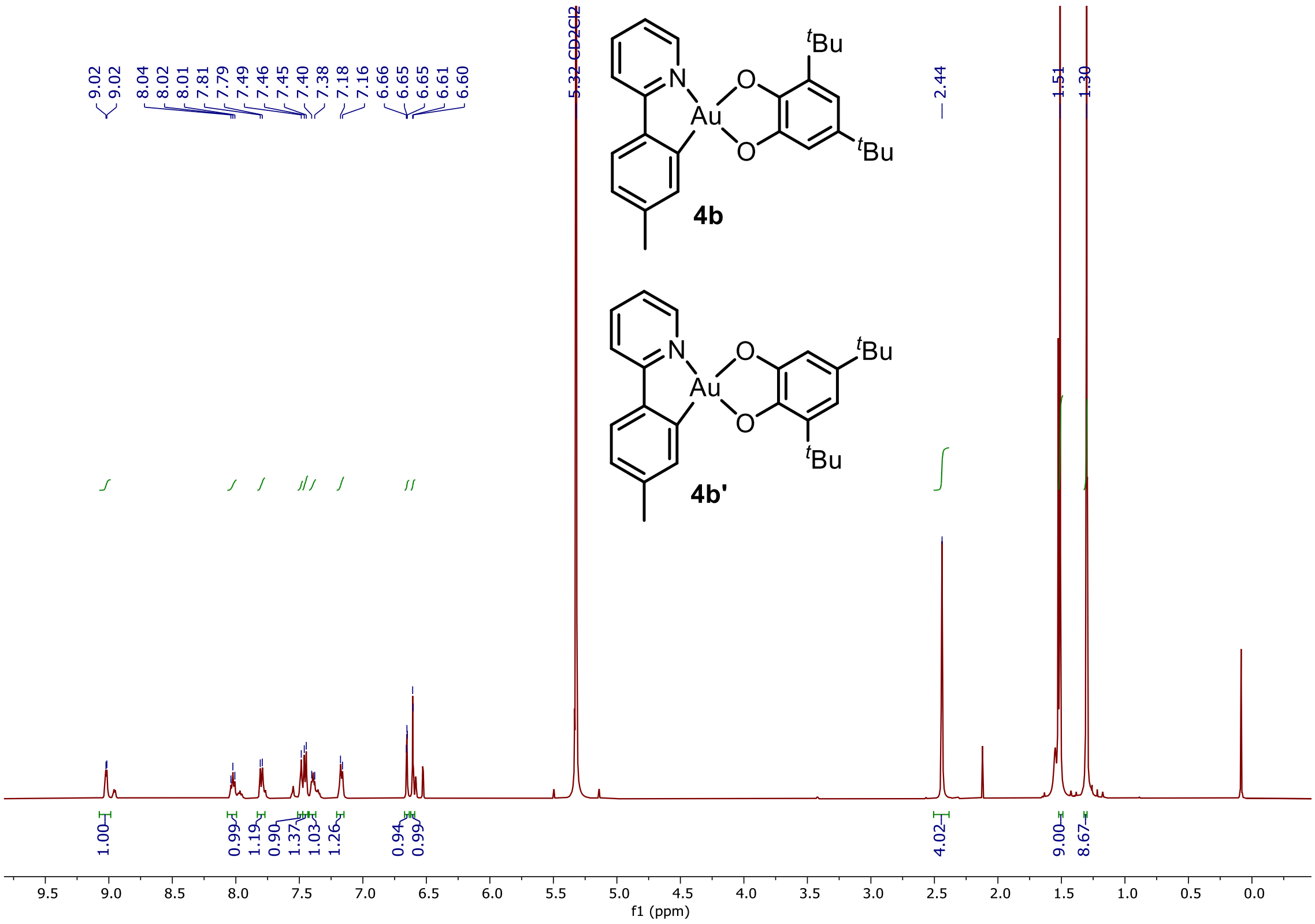

Figure S20. ^{1}H NMR Spectrum (500 MHz, 298 K, CD_2Cl_2) of gold complex **4b** (diastereomer mixture).

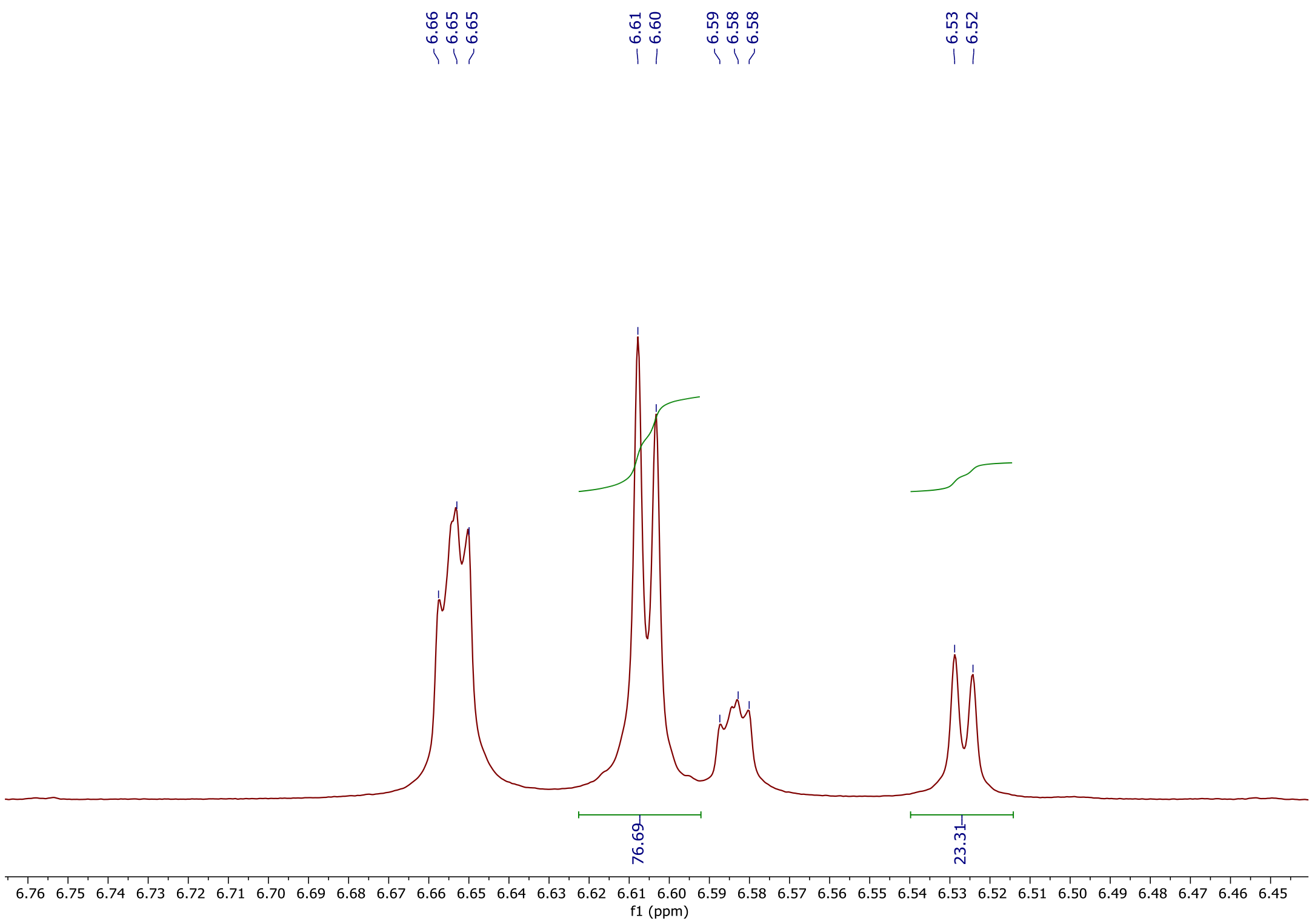

Figure S21. ^{1}H NMR Spectrum (500 MHz, 298 K, CD_2Cl_2); close-up of α-H to the pyridine unit of gold complex **4b**. The ratio is used to determine the diastereomer mixture of **4b** and **4b'**; with NOESY NMR (below) we determined which is the major isomer.

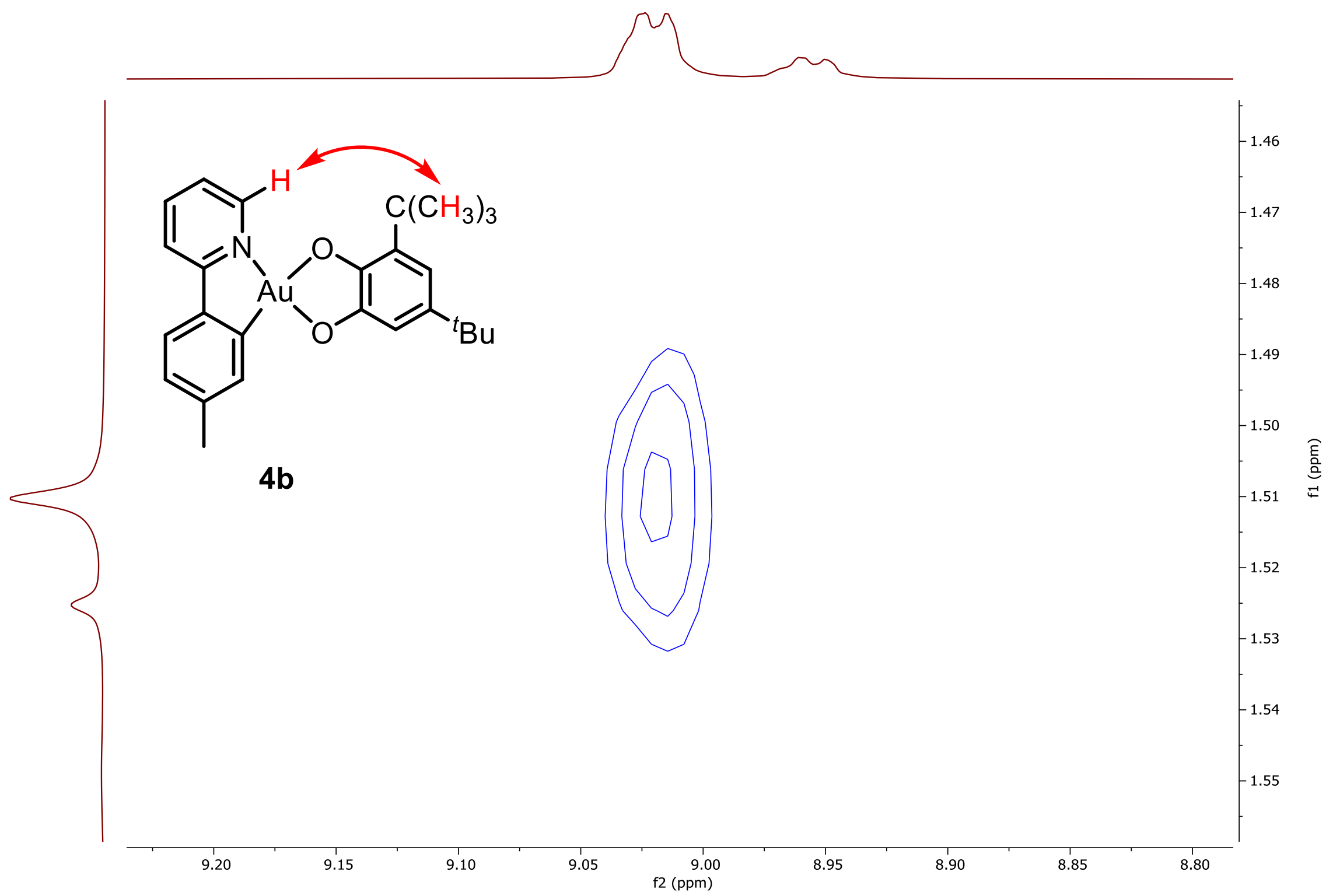

Figure S22. NOESY NMR Spectrum (500 MHz, 298 K, CD_2Cl_2); close-up showing the correlation between the α-H to the pyridine unit and the tertbutyl catecholate protons used to determine the major isomer for gold complex **4b**.

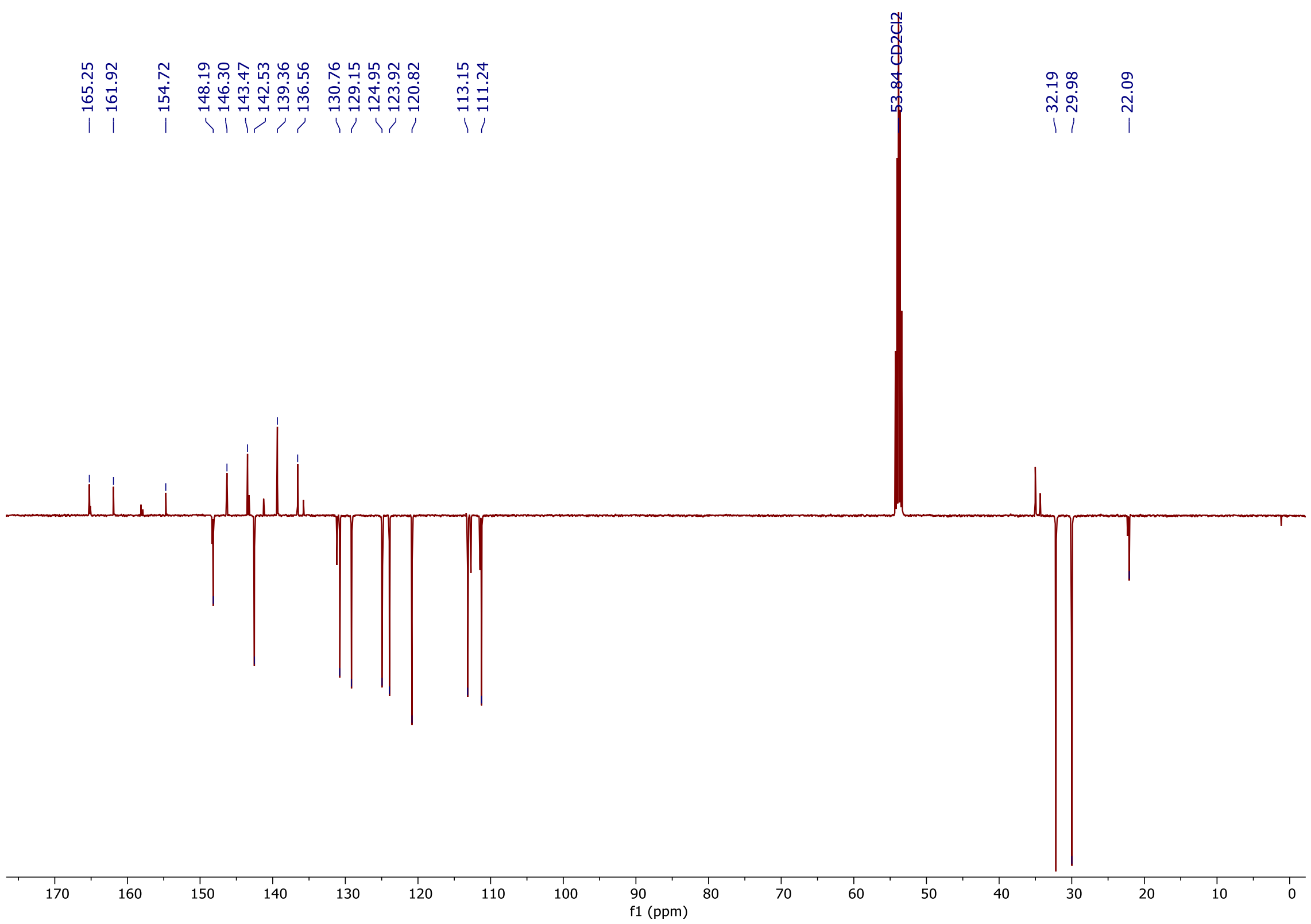

Figure S23. $^{13}C\{1H\}$ JMOD NMR Spectrum (126 MHz, 298 K, CD_2Cl_2) of gold complex **4b**.

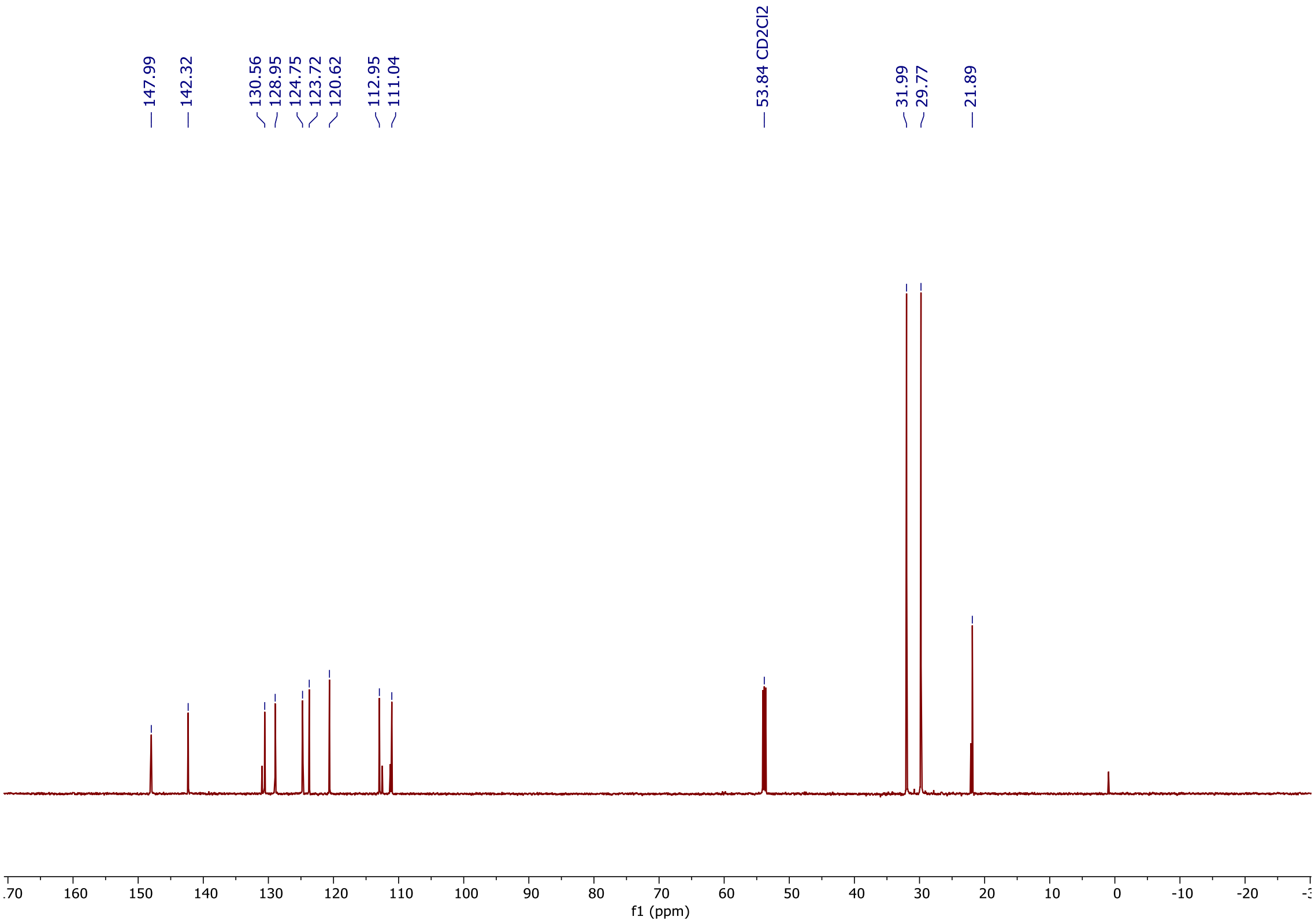

Figure S24. $^{13}C\{1H\}$ NMR Spectrum (126 MHz, 298 K, CD_2Cl_2) of gold complex **4b**.

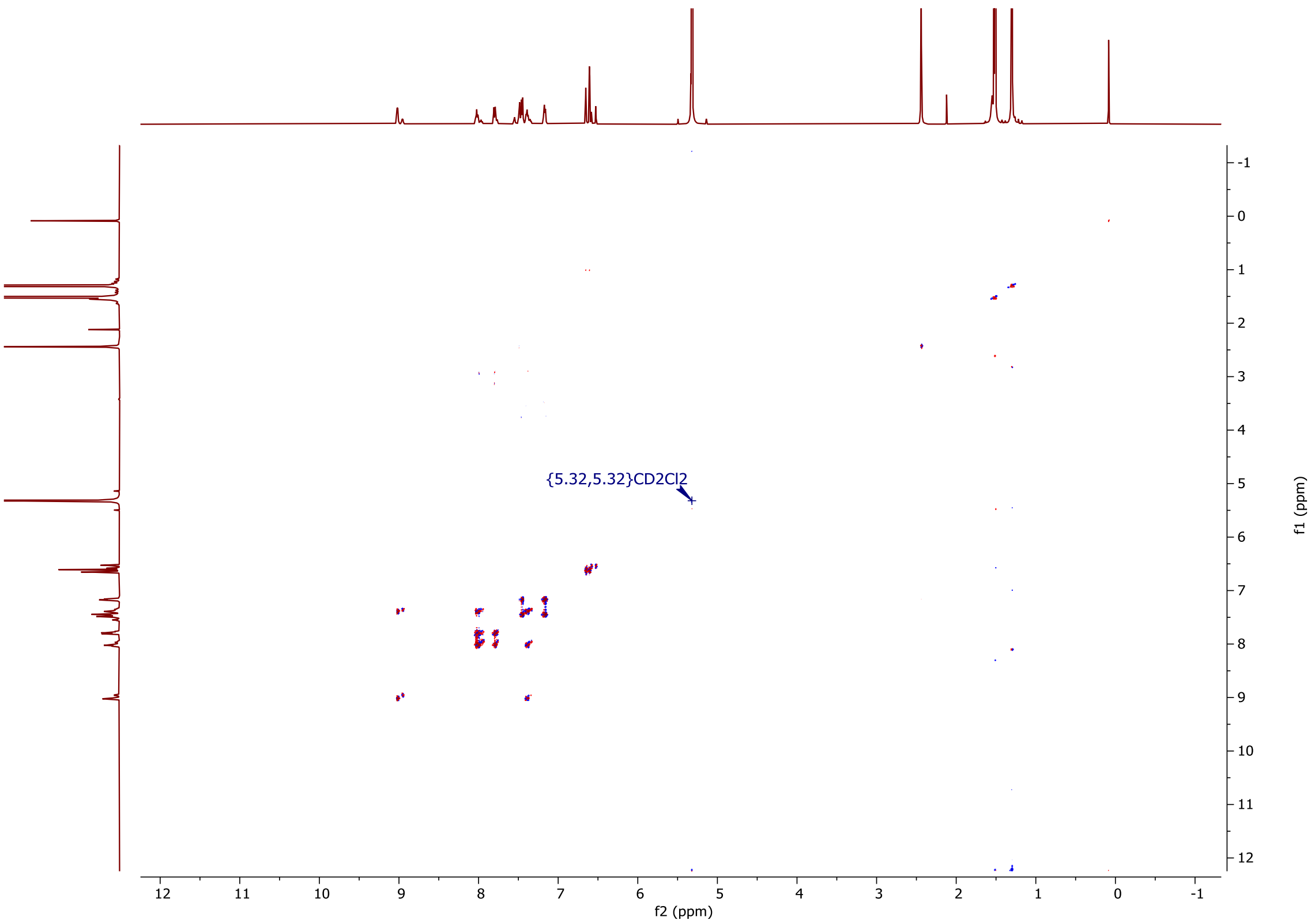

Figure S25. COSY NMR Spectrum (500 MHz, 298 K, CD_2Cl_2) of gold complex **4b**.

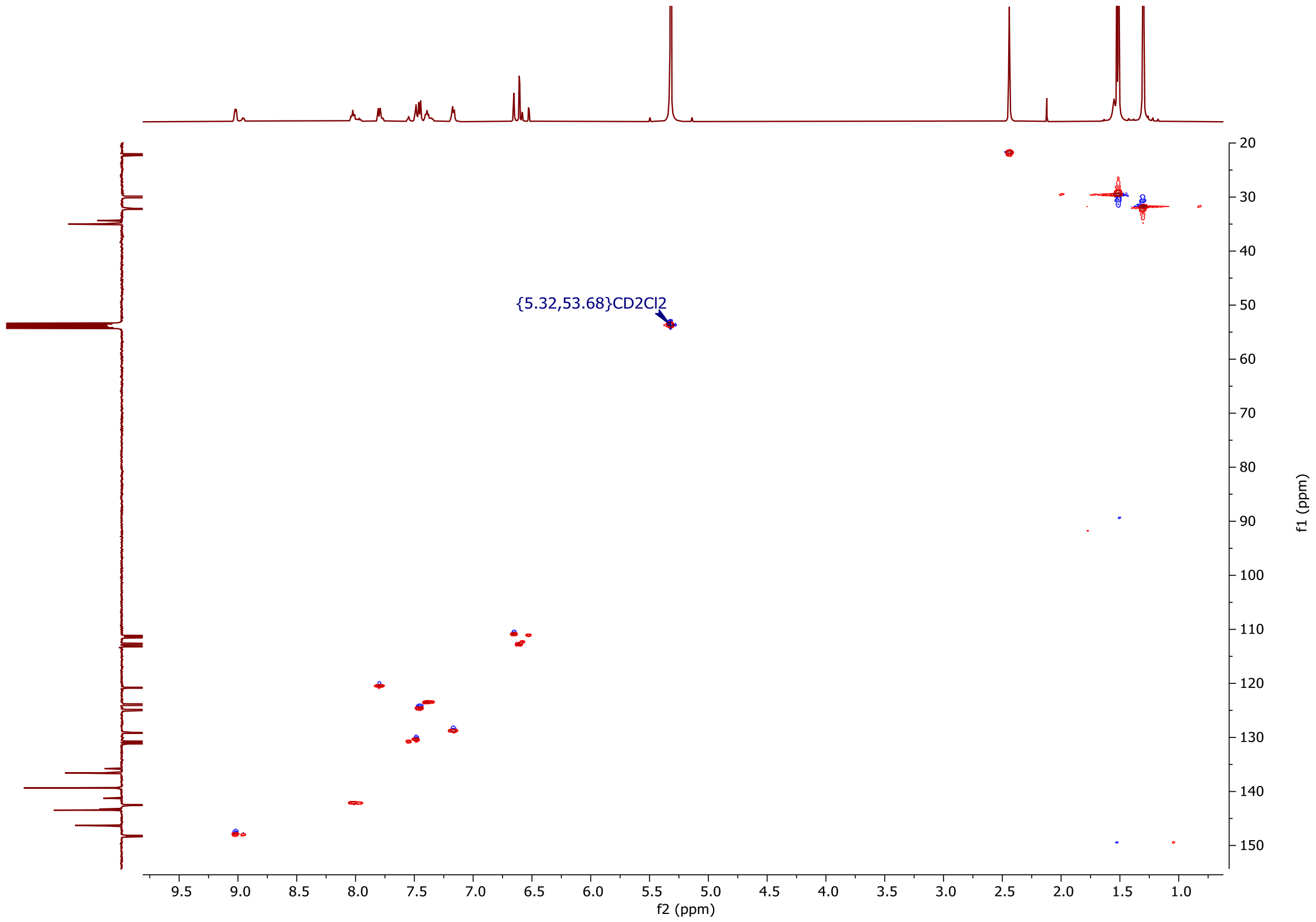

Figure S26. HSQC NMR Spectrum (500, 126 MHz, 298 K, CD_2Cl_2) of gold complex **4b**.

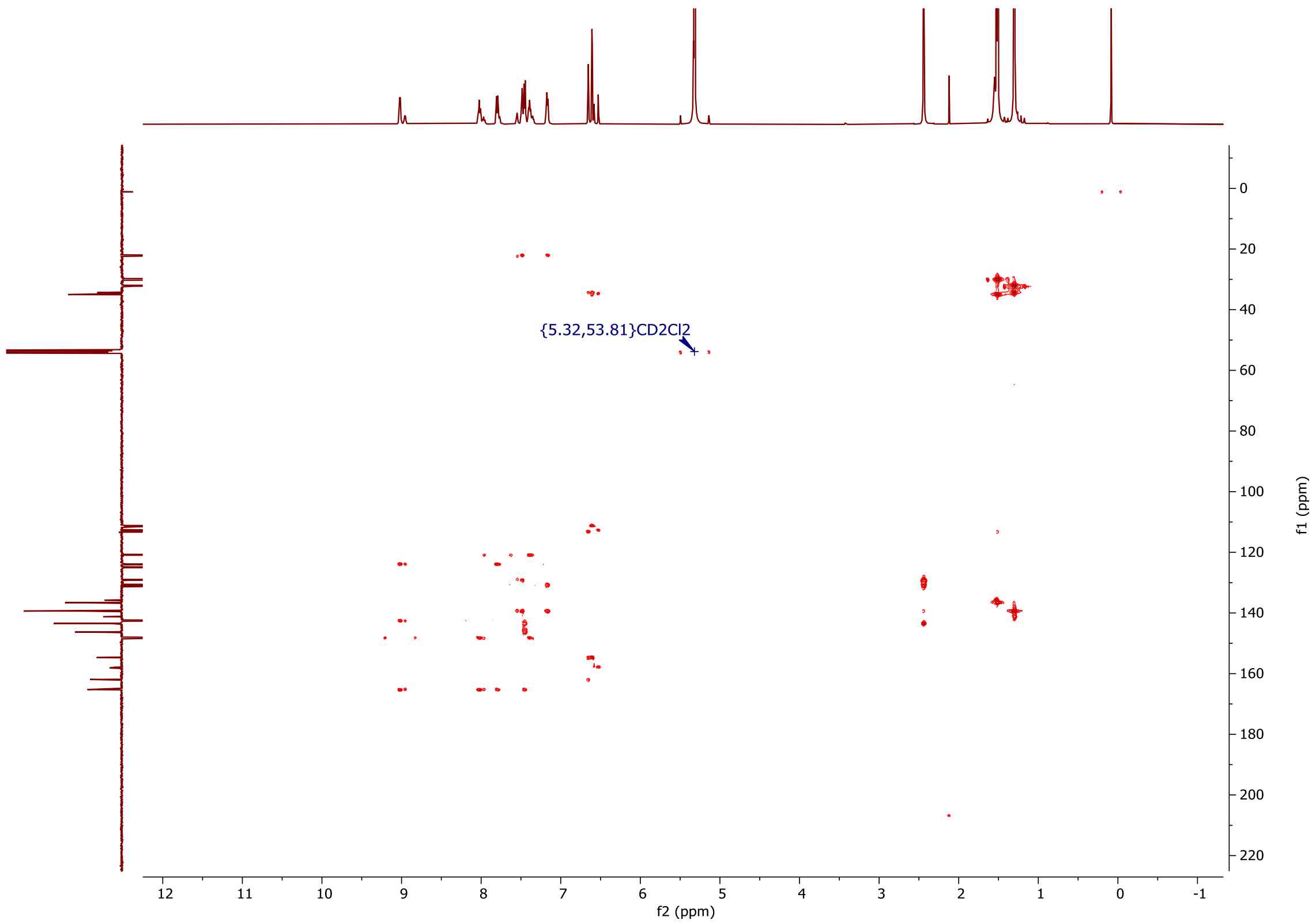

Figure S27. HMBC NMR Spectrum (500, 126 MHz, 298 K, CD_2Cl_2) of gold complex **4b**.

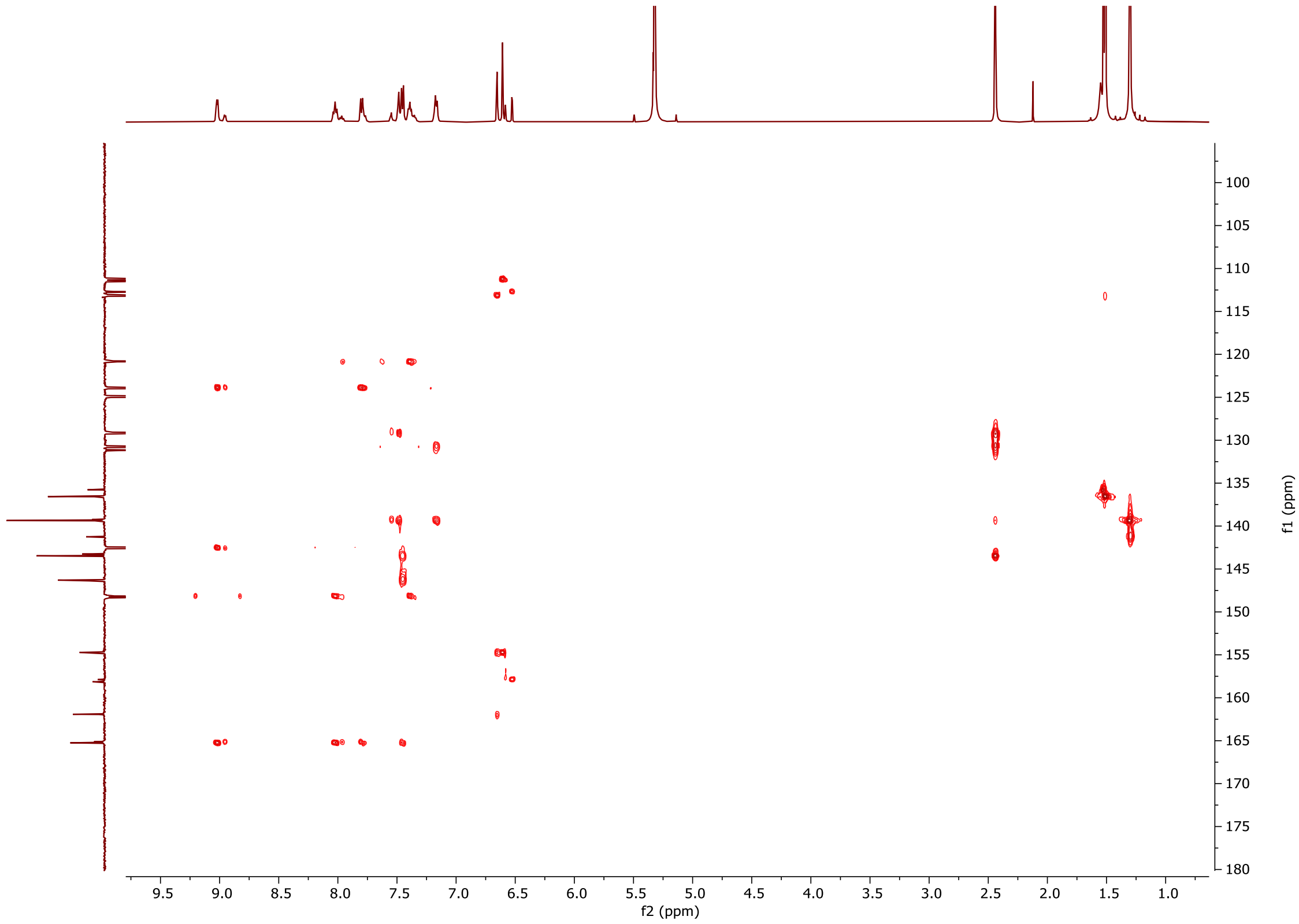

Figure S28. HMBC NMR Spectrum (500, 126 MHz, 298 K, CD_2Cl_2); close-up of aromatic region of gold complex **4b**.

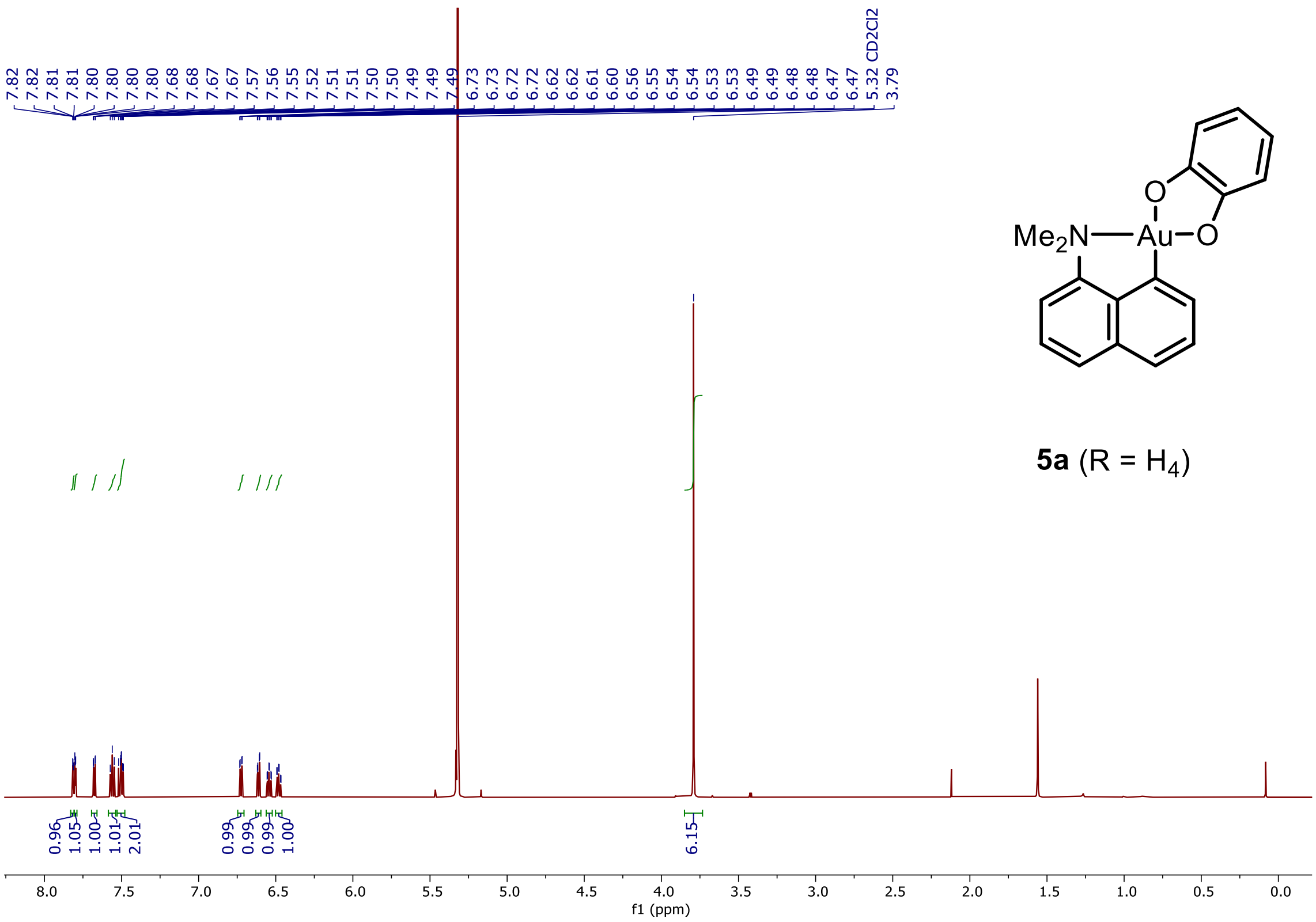

Figure S29. ^{1}H NMR Spectrum (600 MHz, 298 K, CD_2Cl_2) of gold complex **5a**.

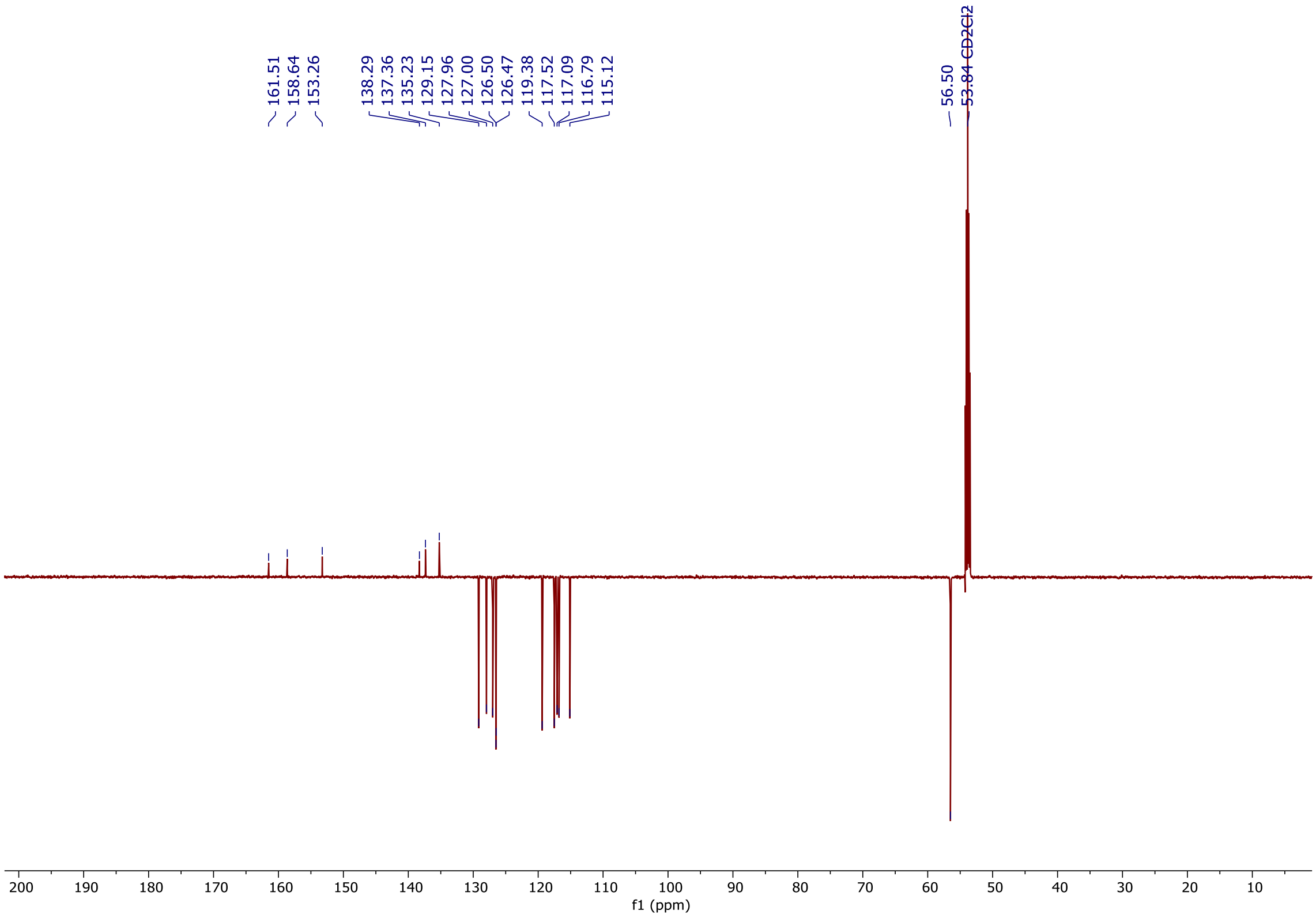

Figure S30. $^{13}C\{1H\}$ JMOD NMR Spectrum (151 MHz, 298 K, CD_2Cl_2) of gold complex **5a**.

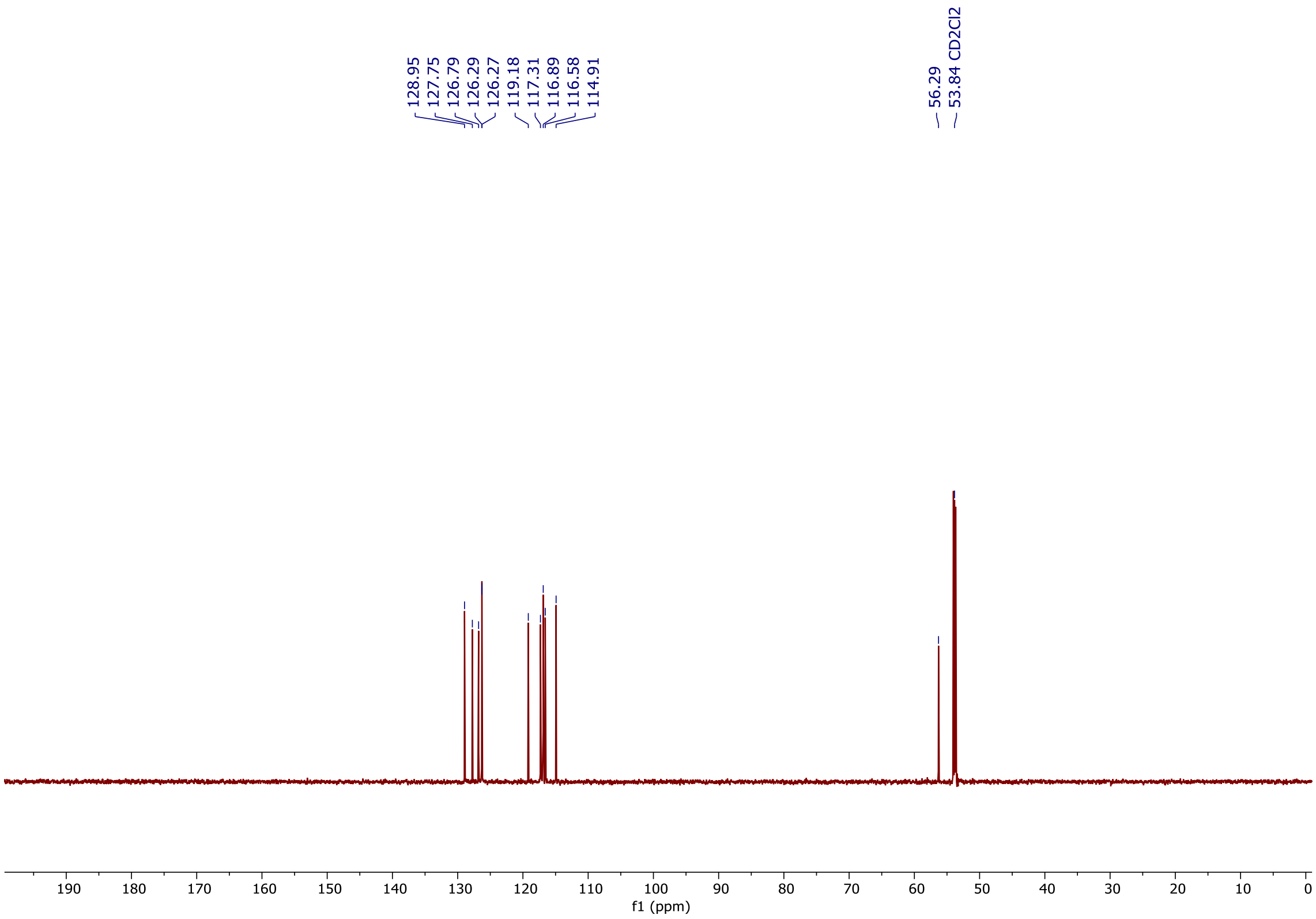

Figure S31. ^{13}C JMOD NMR Spectrum (151 MHz, 298 K, CD_2Cl_2) of gold complex **5a**.

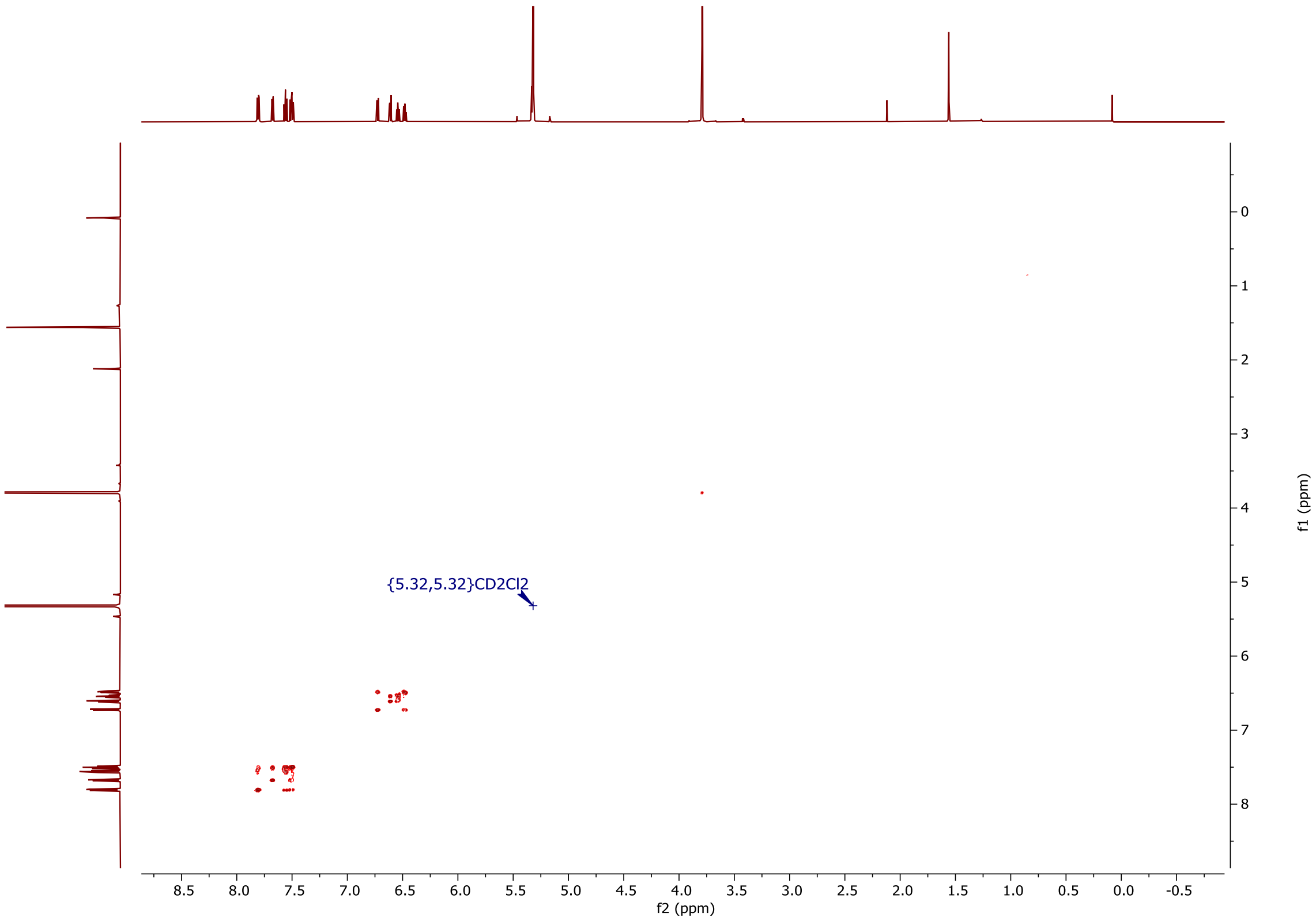

Figure S32. COSY NMR Spectrum (600 MHz, 298 K, CD_2Cl_2) of gold complex **5a**.

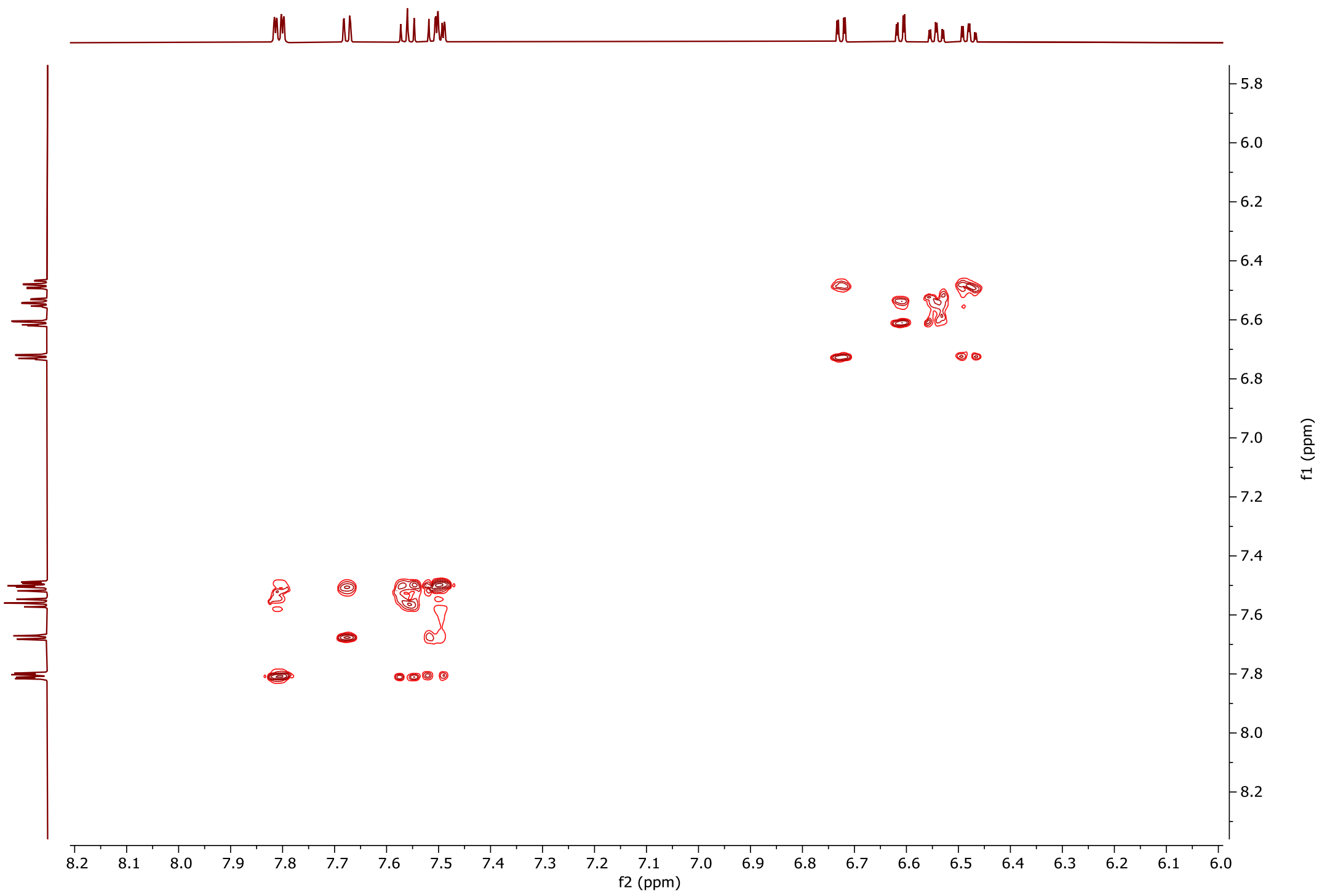

Figure S33. COSY NMR Spectrum (600 MHz, 298 K, CD_2Cl_2);close-up of aromatic region of gold complex **5a**.

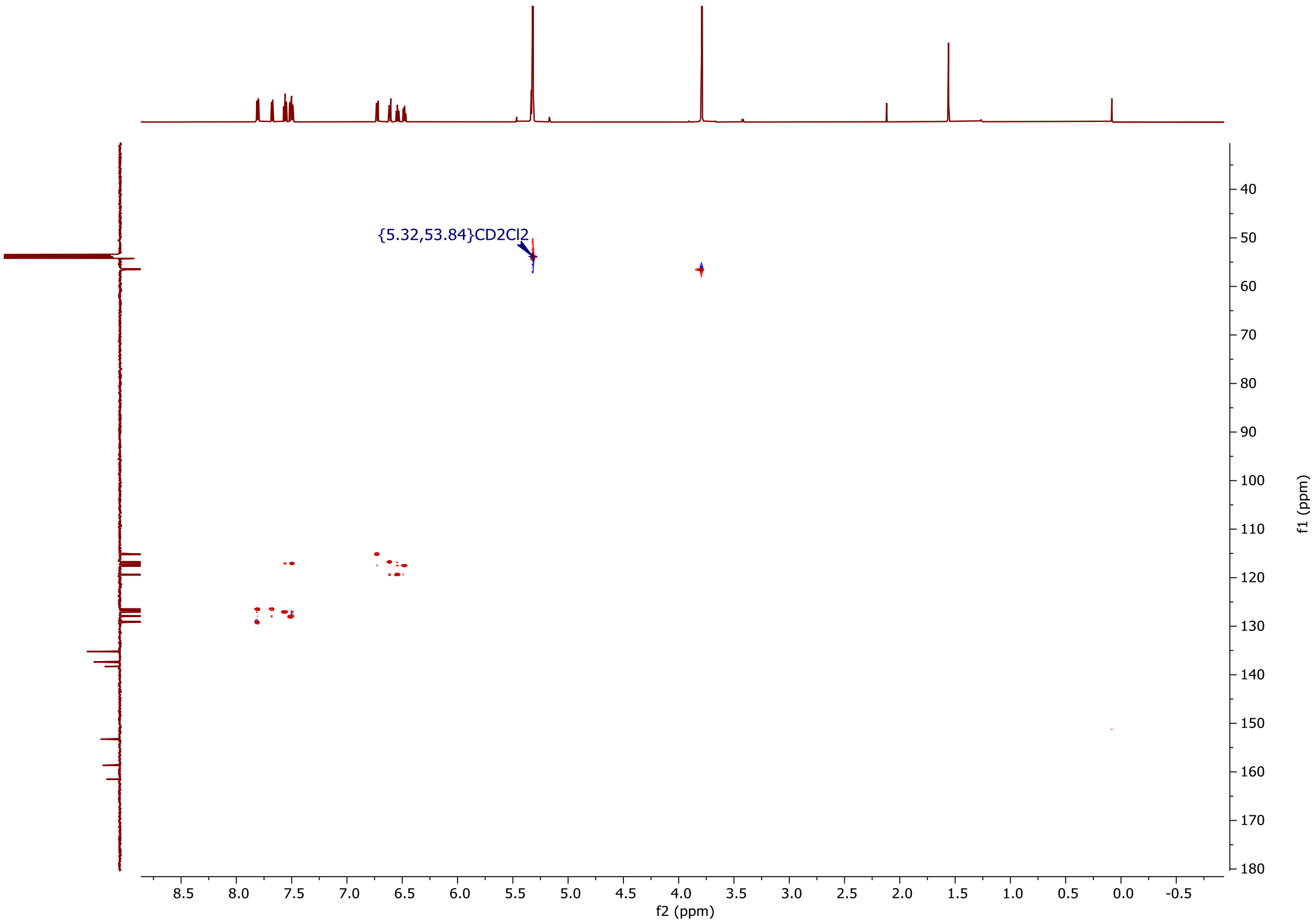

Figure S34. HSQC NMR Spectrum (600, 151 MHz, 298 K, CD_2Cl_2) of gold complex **5a**.

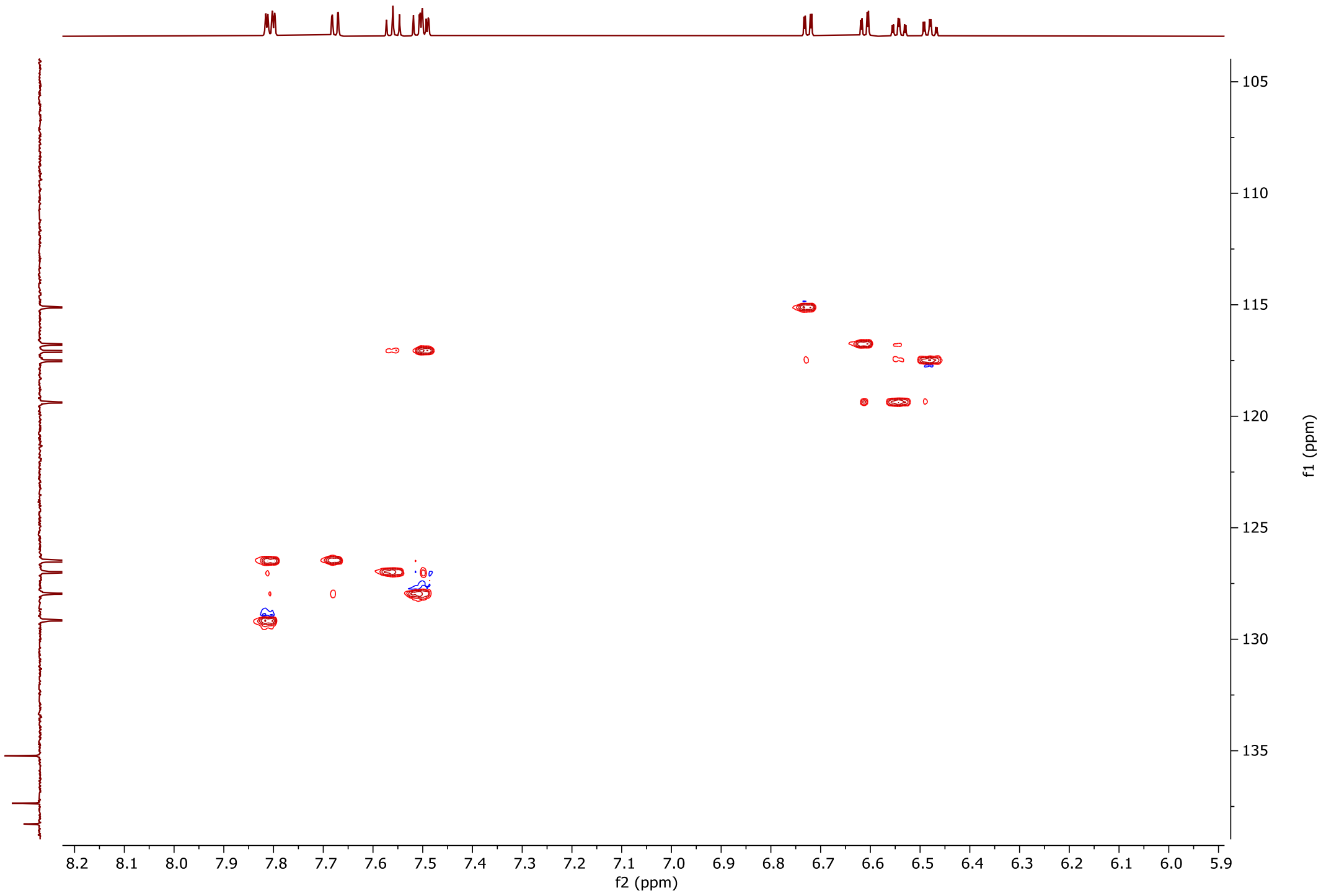

Figure S35. HSQC NMR Spectrum (600, 151 MHz, 298 K, CD_2Cl_2);close-up of aromatic region of gold complex **5a**.

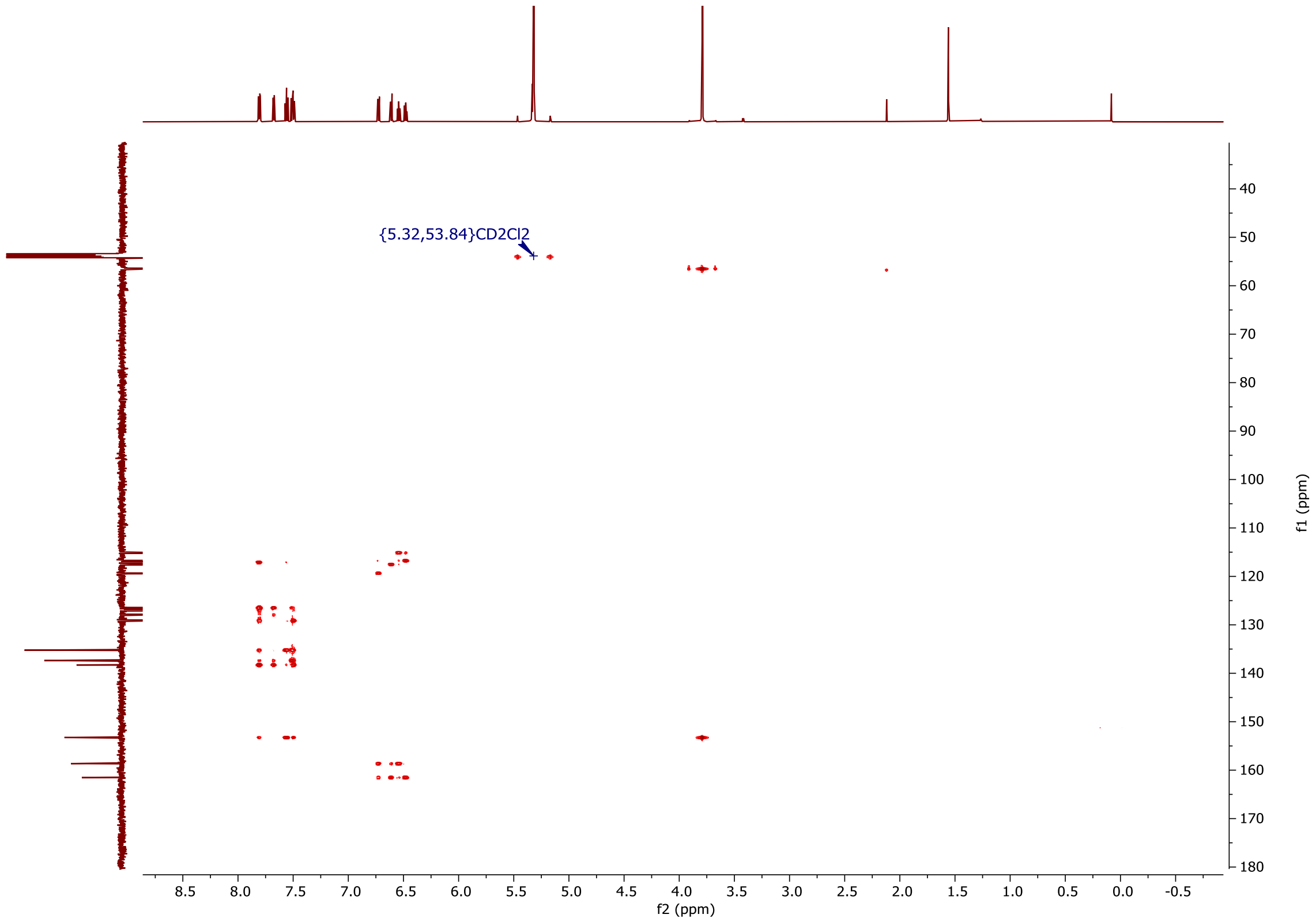

Figure S36. HMBC NMR Spectrum (600, 151 MHz, 298 K, CD_2Cl_2) of gold complex **5a**.

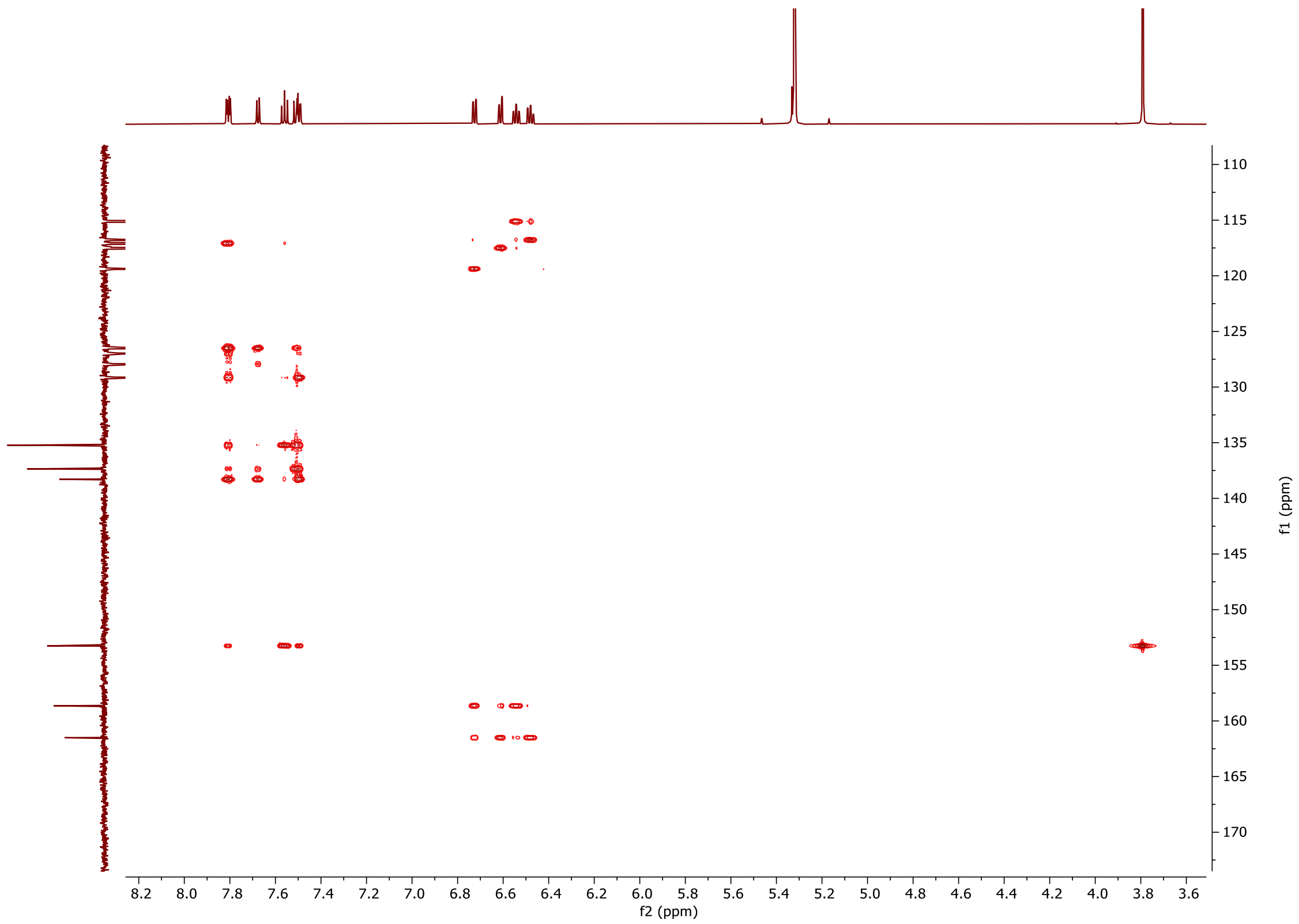

Figure S37. HMBC NMR Spectrum (600, 151 MHz, 298 K, CD_2Cl_2); close-up of aromatic region of gold complex **5a**.

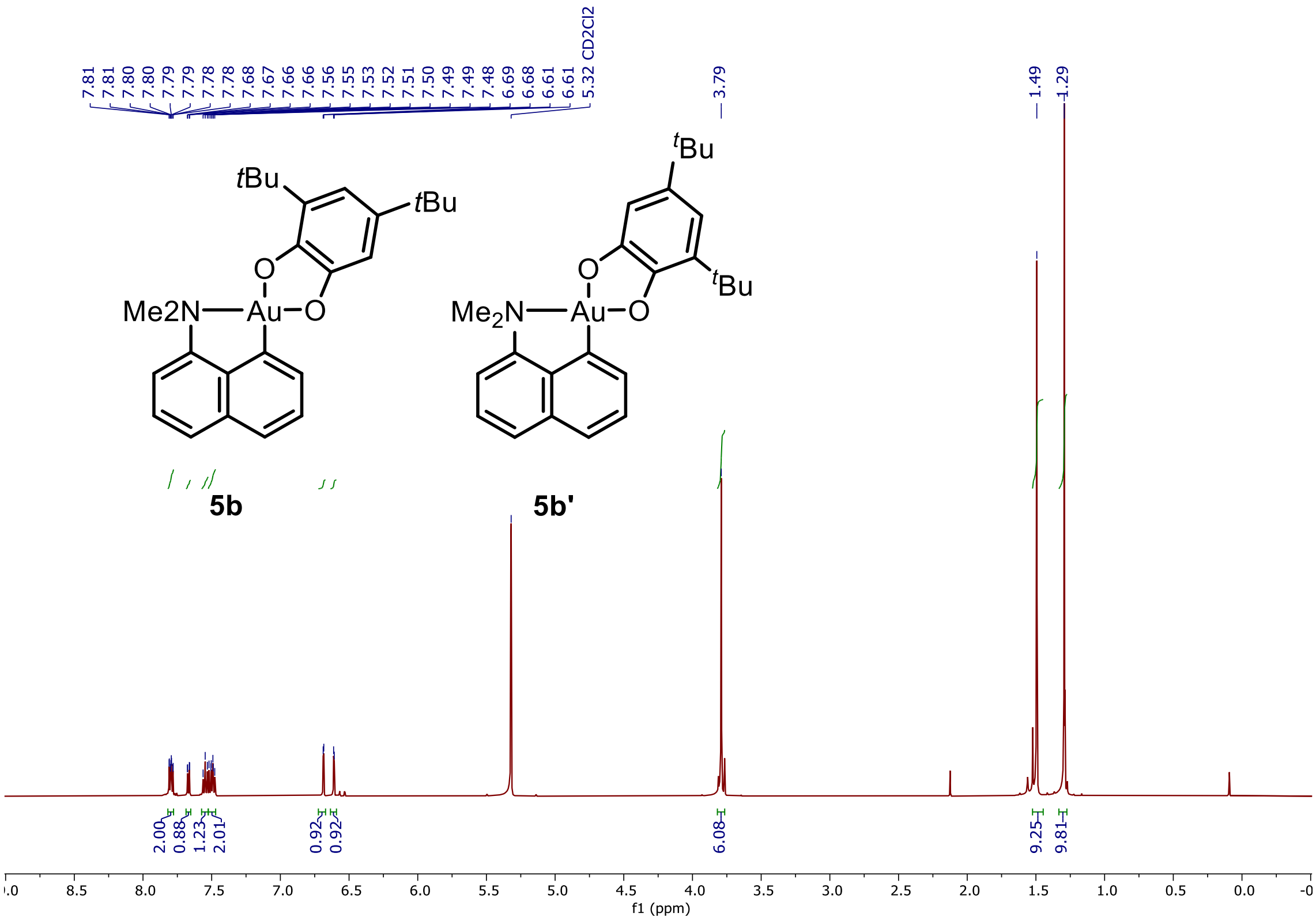

Figure S38. ^{1}H NMR Spectrum (500 MHz, 298 K, CD_2Cl_2) of gold complex **5b** (diastereomer mixture).

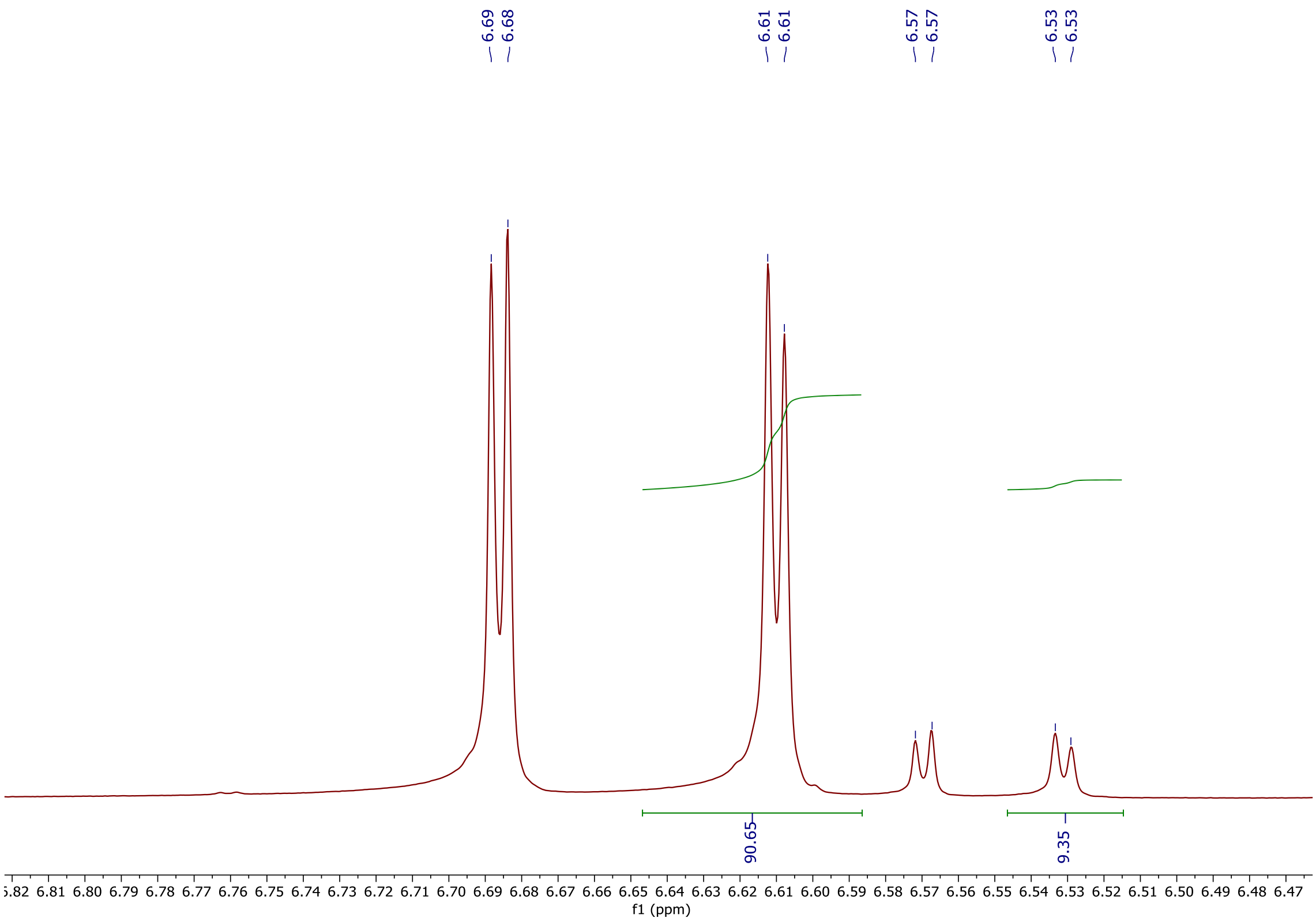

Figure S39. ^{1}H NMR Spectrum (500 MHz, 298 K, CD_2Cl_2); close-up of α-H to the pyridine unit of gold complex **5b**. The ratio is used to determine the diastereomer mixture of **5b** and **5b'**; with NOESY NMR (below) we determined which is the major isomer.

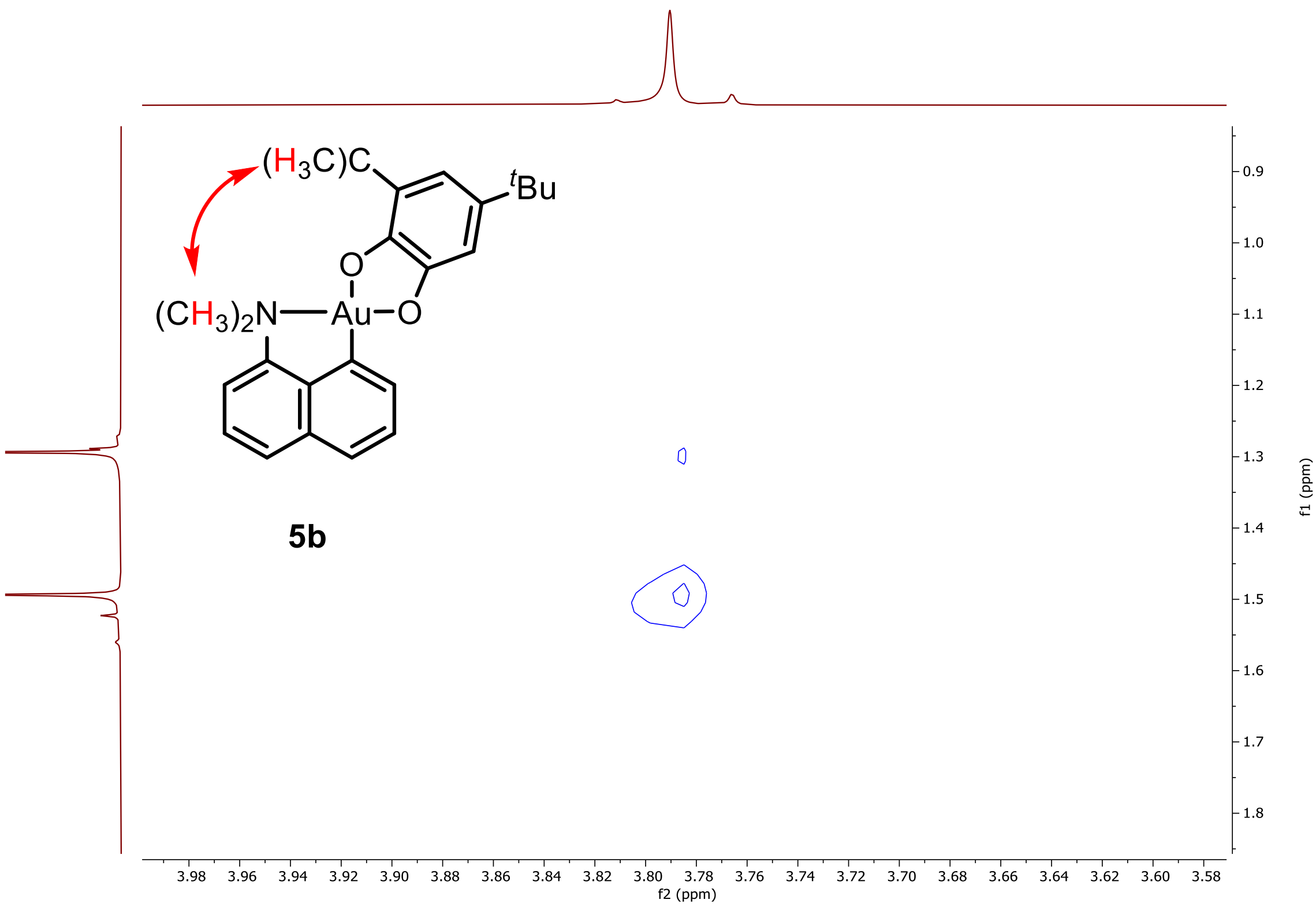

Figure S40. NOESY NMR Spectrum (500 MHz, 298 K, CD_2Cl_2); close-up showing the correlation between the NMe_2 and the tertbutyl catecholate protons used to determine the major isomer for gold complex **5b**.

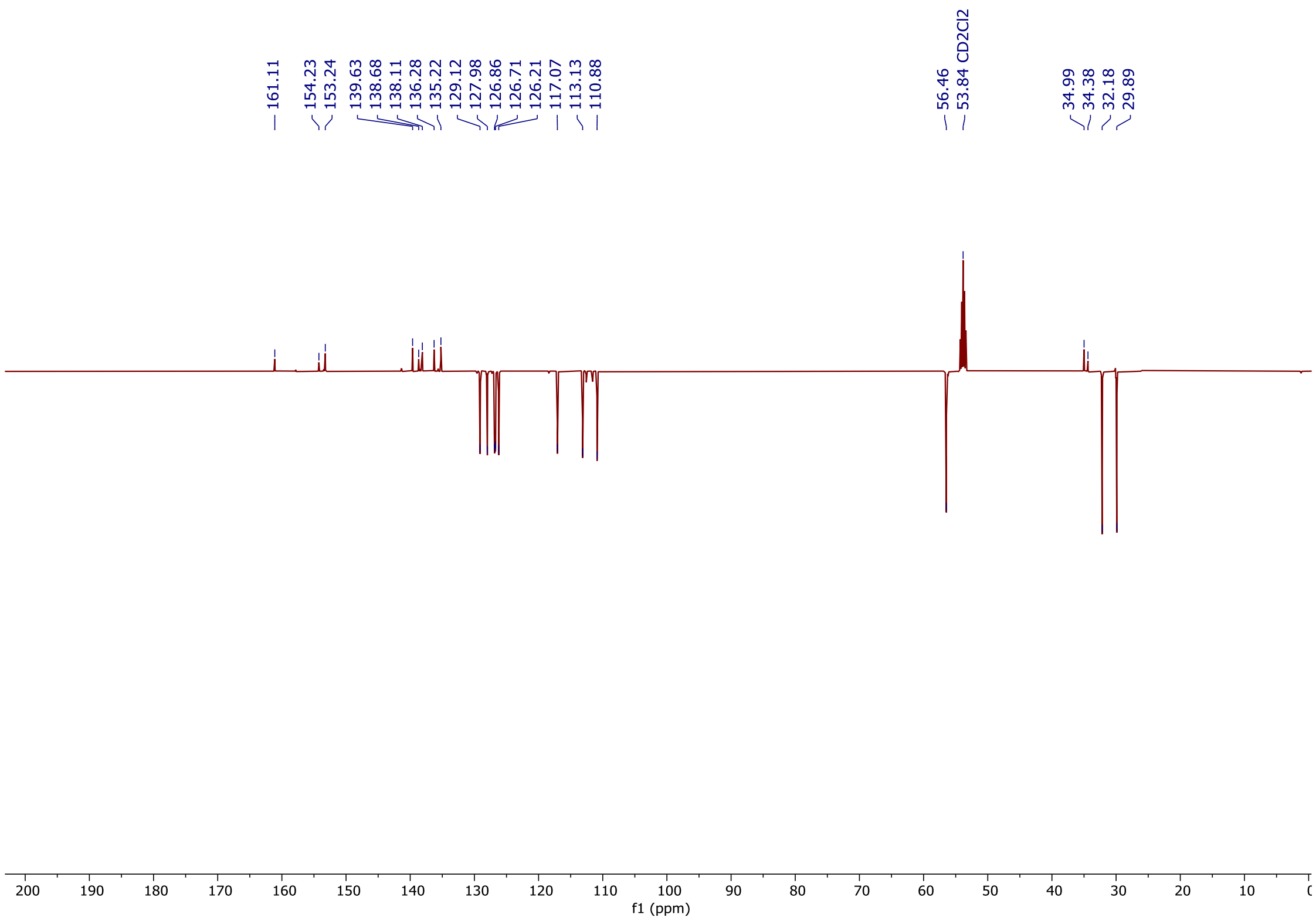

Figure S41. $^{13}C\{^1H\}$ JMOD NMR Spectrum (126 MHz, 298 K, CD_2Cl_2) of gold complex **5b**.

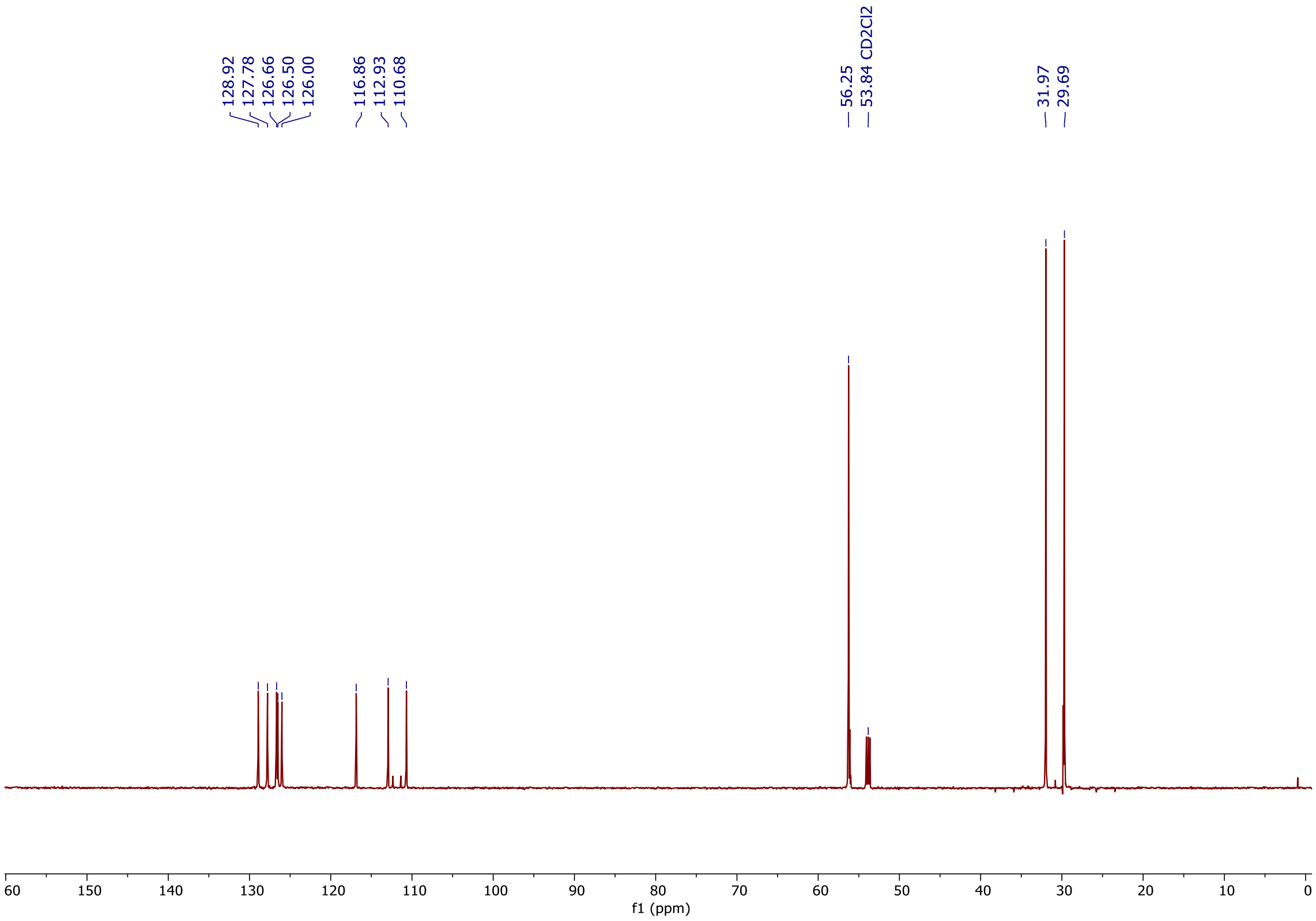

Figure S42. $^{13}C\{1H\}$ JMOD NMR Spectrum (126 MHz, 298 K, CD_2Cl_2) of gold complex **5b**.

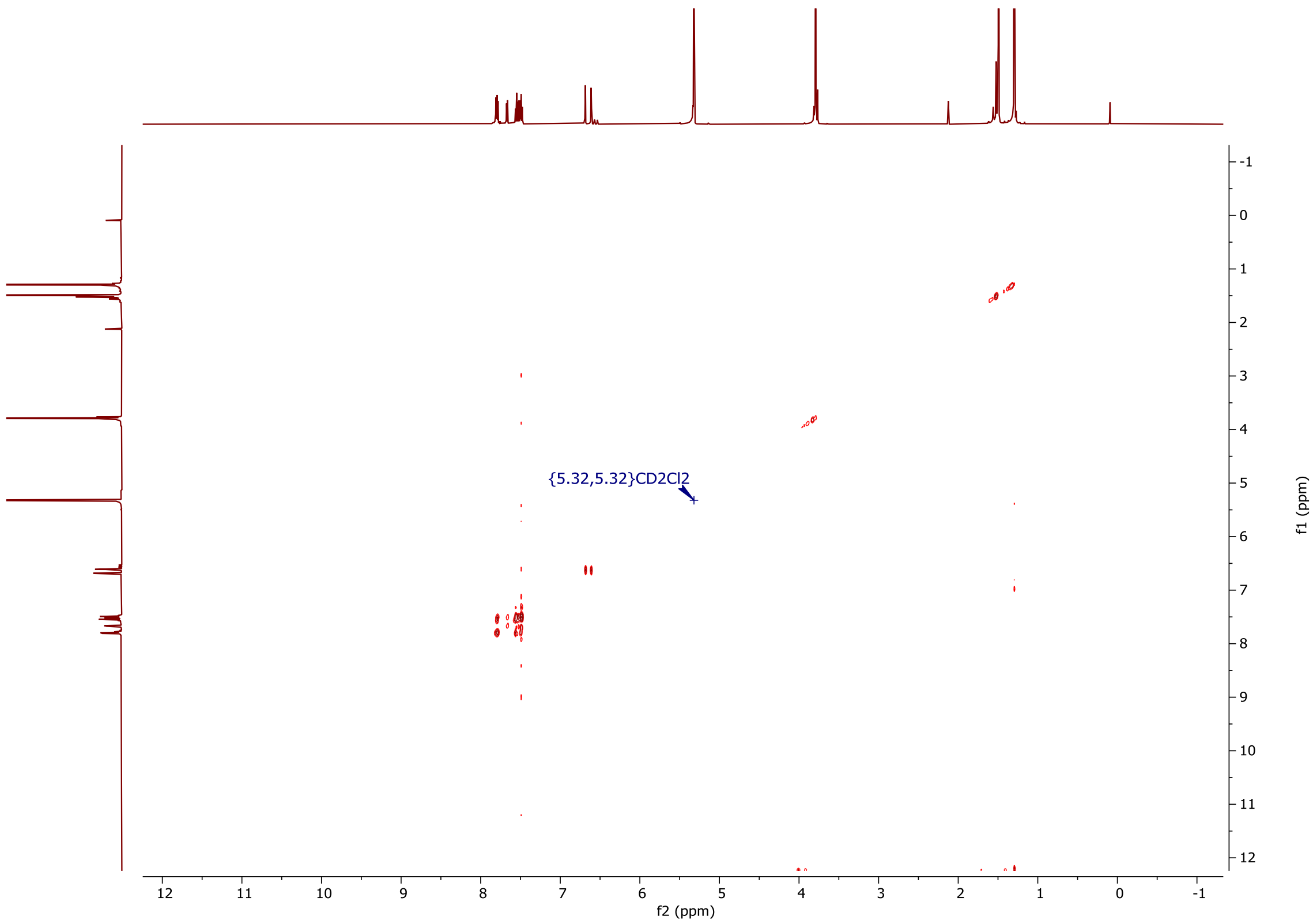

Figure S43. COSY NMR Spectrum (500 MHz, 298 K, CD_2Cl_2) of gold complex **5b**.

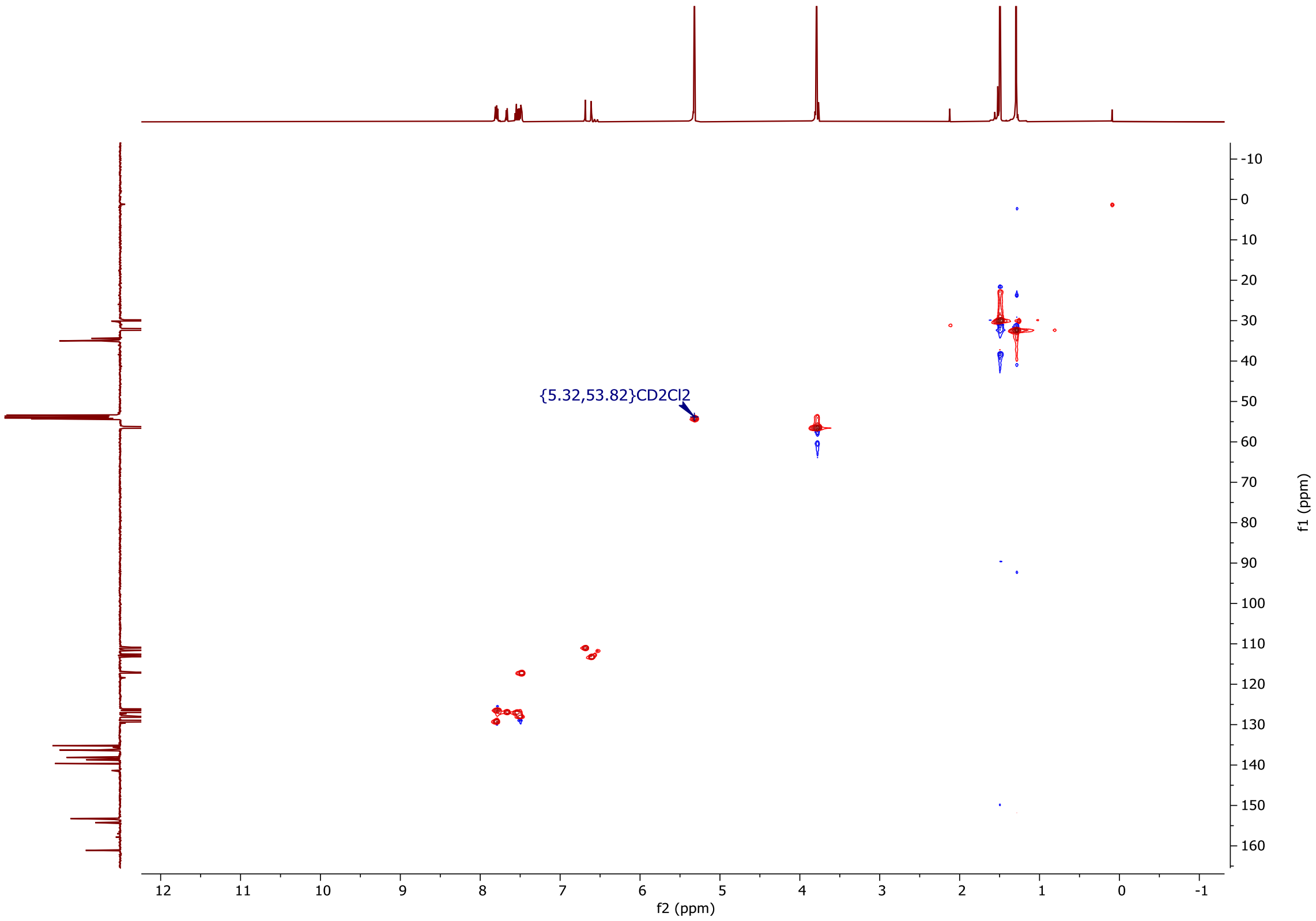

Figure S44. HSQC NMR Spectrum (500, 126 MHz, 298 K, CD_2Cl_2) of gold complex **5b**.

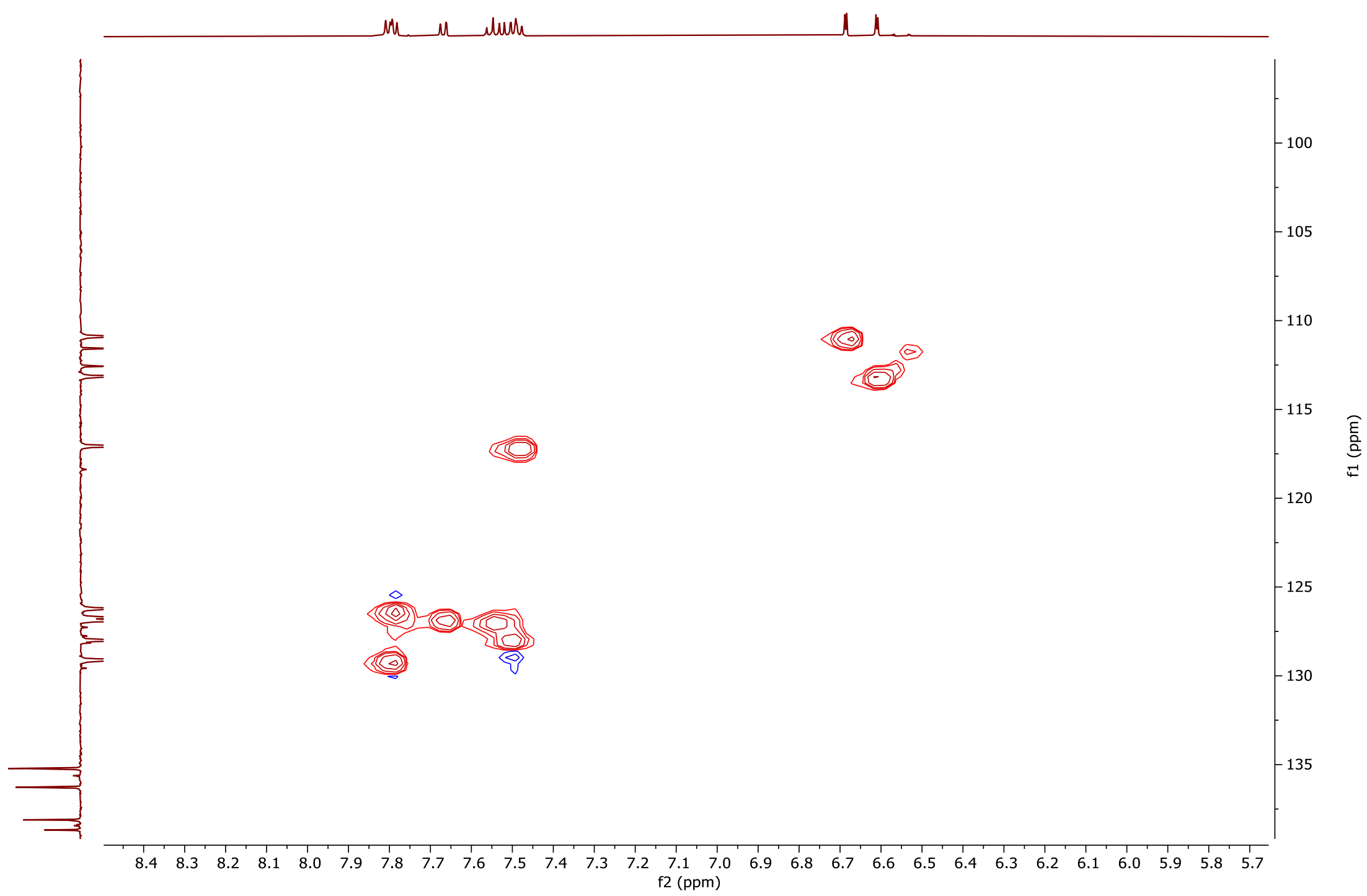

Figure S45. HSQC NMR Spectrum (500, 126 MHz, 298 K, CD_2Cl_2); close-up of aromatic region of gold complex **5b**.

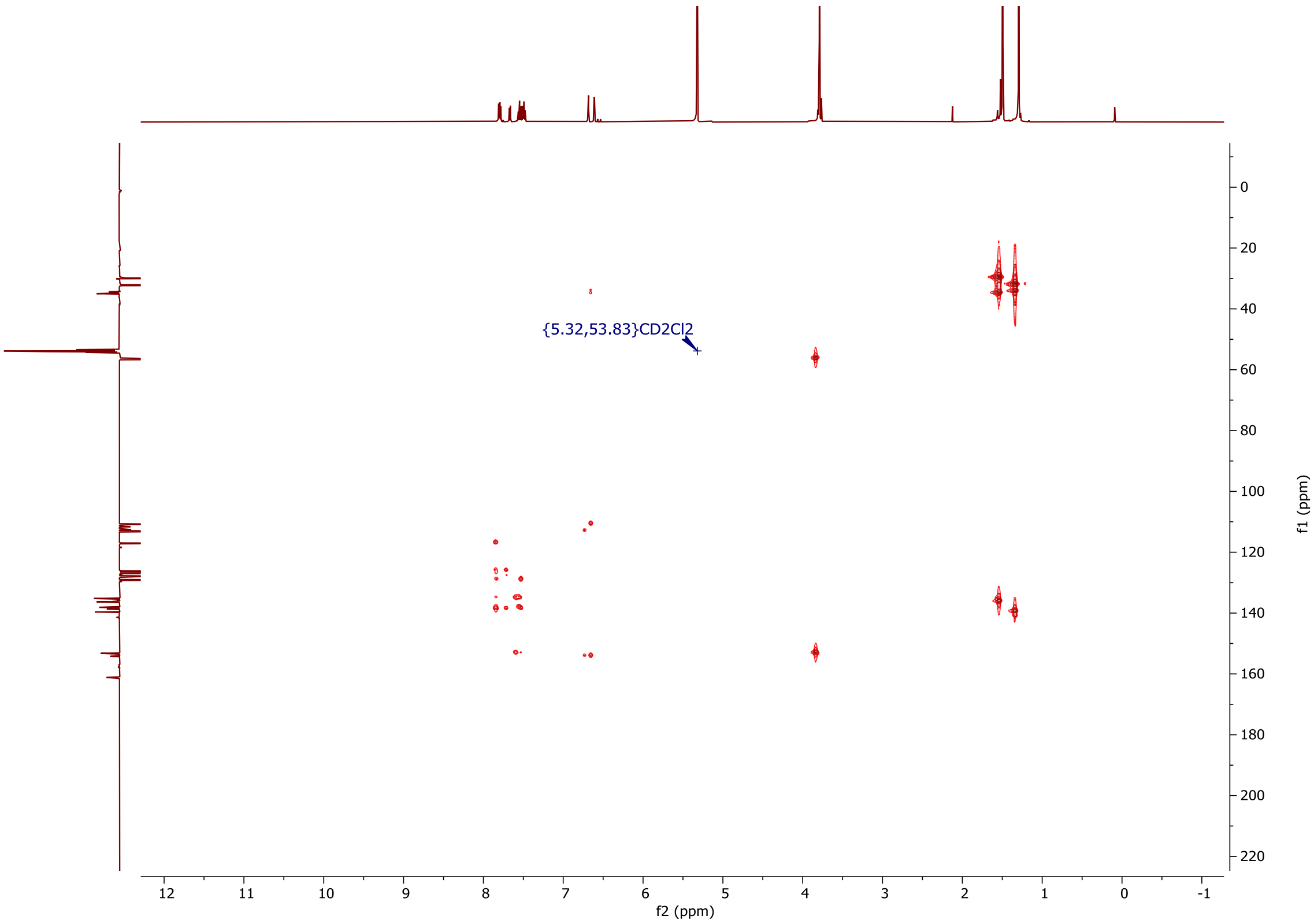

Figure S46. HMBC NMR Spectrum (500, 126 MHz, 298 K, CD_2Cl_2) of gold complex **5b**.

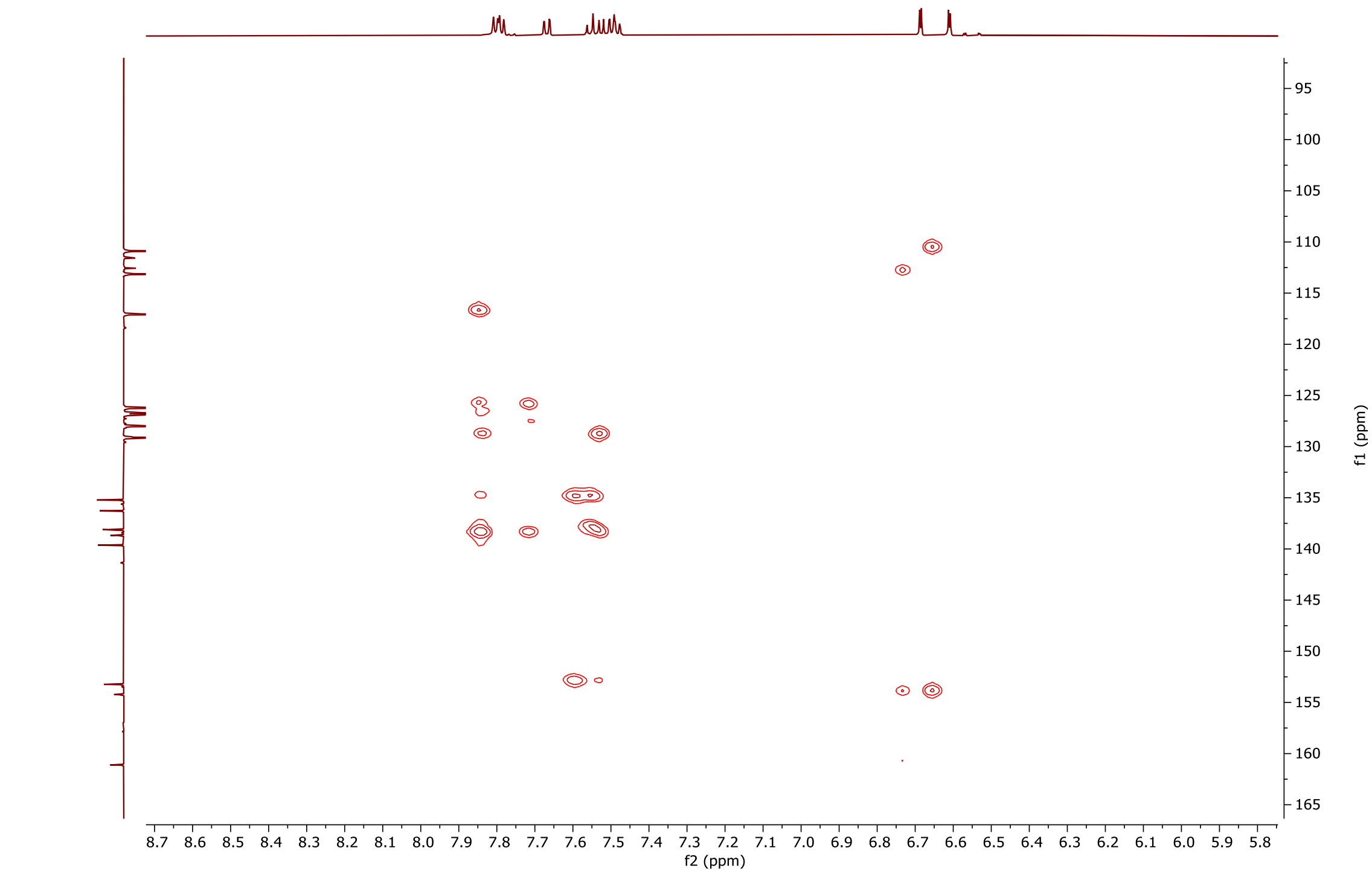

Figure S47. HSQC NMR Spectrum (500, 126 MHz, 298 K, CD_2Cl_2); close-up of aromatic region of gold complex **5b**.

7. xyz coordinates and energies in a.u.

Level of theory for optimization: SMD(DCM)-PBE0-D3(BJ)/SDD+f(Au), 6-31G**(other atoms) **Into square brackets [], single-point calculations at** SMD(DCM)-ZORA-PBE0-D4/SARC-TZVP(Au), def2-TZVP(other atoms)//SMD(DCM)-PBE0-D3(BJ)/SDD+f(Au), 6-31G**(other atoms).

P^C-H_4

E(RPBE1PBE) = -1479.20186672
Zero-point correction= 0.408857 (Hartree/Particle)
Thermal correction to Energy= 0.434040
Thermal correction to Enthalpy= 0.434984
Thermal correction to Gibbs Free Energy= 0.353778
Sum of electronic and zero-point Energies= -1478.793010
Sum of electronic and thermal Energies= -1478.767827
Sum of electronic and thermal Enthalpies= -1478.766883
Sum of electronic and thermal Free Energies= -1478.848089

Charge = 0 Multiplicity = 1
Au -0.154547 0.003698 0.049302
P 0.283026 0.291920 2.267422
O -0.574807 1.960653 -0.389594
O -0.529630 -0.267246 -1.926458
C 0.207997 -1.935977 0.432077
C 0.576272 -1.403407 2.820959
C 0.502629 -2.350872 1.764178
C 0.722344 -3.729845 2.051717
C 0.995438 -4.105849 3.390155
H 1.160329 -5.157228 3.611458
C 1.050925 -3.169009 4.395578
H 1.258897 -3.475616 5.416049
C 0.842962 -1.802060 4.115250
H 0.898119 -1.077989 4.921109
C -0.866572 2.075537 -1.698946
C -1.187290 3.303151 -2.277565
C -1.491562 3.383970 -3.639696
C -1.476352 2.236637 -4.427587
C -1.154751 1.000360 -3.859118
C -0.848222 0.909843 -2.502376
C 0.653679 -4.667420 0.991760
C 0.150146 -2.878711 -0.569906
H -0.076073 -2.570023 -1.586430
C 0.376525 -4.246734 -0.285833
H 0.821761 -5.718939 1.208166
H 0.324366 -4.968189 -1.096579
H -1.136996 0.094346 -4.459992
H -1.712688 2.294666 -5.486696
H -1.740396 4.346941 -4.077666
H -1.195818 4.189483 -1.647536
C 1.786857 1.341371 2.477120
H 2.442956 0.958725 1.684191
C 2.503830 1.175540 3.812716
C 1.438399 2.795768 2.164853
H 0.918952 2.893698 1.205977
H 0.809209 3.229463 2.948861
H 2.363012 3.379776 2.117570
H 1.893085 1.507543 4.656429
H 3.408946 1.791921 3.796247
H 2.809539 0.141037 3.986711
C -1.201421 1.012952 3.082578
H -1.352491 1.951624 2.533395
C -1.013363 1.321825 4.564308
C -2.398949 0.098330 2.834831
H -2.561057 -0.082053 1.767231
H -2.269395 -0.868156 3.332728
H -3.299612 0.570097 3.240136
H -0.826341 0.413412 5.144386
H -1.934166 1.774369 4.947097
H -0.197703 2.026668 4.740829

P^C-H_4-sq

E(UPBE1PBE) = -1479.03616035
Zero-point correction = 0.409998 (Hartree/Particle)
Thermal correction to Energy = 0.435151
Thermal correction to Enthalpy = 0.436095
Thermal correction to Gibbs Free Energy = 0.354352
Sum of electronic and zero-point Energies = -1478.626162
Sum of electronic and thermal Energies = -1478.601009
Sum of electronic and thermal Enthalpies = -1478.600065
Sum of electronic and thermal Free Energies = -1478.681808
[FINAL SINGLE POINT ENERGY = -21021.454159774388]

Charge = 1 Multiplicity = 2
Au -0.150369 -0.008885 0.083042
P 0.296555 0.282970 2.304705
O -0.597854 1.985846 -0.443964
O -0.554157 -0.233738 -1.960261
C 0.219997 -1.935509 0.476491
C 0.583612 -1.406736 2.861170
C 0.511562 -2.349143 1.804844
C 0.730316 -3.728633 2.081208
C 1.001791 -4.109140 3.418452
H 1.166466 -5.160977 3.636113
C 1.056320 -3.175533 4.427393
H 1.263213 -3.486219 5.446522
C 0.849788 -1.807030 4.154928
H 0.905343 -1.085550 4.963281
C -0.883996 2.095564 -1.700447
C -1.215930 3.337426 -2.292902
C -1.510694 3.382129 -3.631251
C -1.489316 2.203584 -4.434781
C -1.172818 0.980646 -3.900356
C -0.861711 0.890803 -2.523460
C 0.662322 -4.656424 1.012671
C 0.160863 -2.858635 -0.540731
H -0.062311 -2.550939 -1.557491
C 0.387736 -4.228649 -0.262702
H 0.829740 -5.709518 1.220234
H 0.337079 -4.942955 -1.079102
H -1.153254 0.076686 -4.500227
H -1.729458 2.284220 -5.490399
H -1.765952 4.329204 -4.096350
H -1.228118 4.225541 -1.669337
C 1.798841 1.341135 2.466506
H 2.430465 0.979136 1.644838
C 2.554608 1.140390 3.776636
C 1.436199 2.802910 2.212246
H 0.905334 2.941937 1.265747
H 0.819111 3.205074 3.021786
H 2.359052 3.389400 2.171554
H 1.970567 1.456307 4.644794
H 3.459118 1.756238 3.743145
H 2.863236 0.102005 3.917553
C -1.199953 1.020807 3.073450
H -1.306544 1.981549 2.553082

C -1.038276 1.275027 4.570265
C -2.409862 0.144073 2.758916
H -2.548630 0.015442 1.680079
H -2.314140 -0.845398 3.217265
H -3.308033 0.620183 3.163778
H -0.917440 0.339538 5.123471
H -1.947111 1.762976 4.936635
H -0.193311 1.930868 4.792362

4b

E(RPBE1PBE) = -1348.53530701
Zero-point correction = 0.503121 (Hartree/Particle)
Thermal correction to Energy = 0.532640
Thermal correction to Enthalpy = 0.533584
Thermal correction to Gibbs Free Energy = 0.441925
Sum of electronic and zero-point Energies = -1348.032186
Sum of electronic and thermal Energies = -1348.002667
Sum of electronic and thermal Enthalpies = -1348.001723
Sum of electronic and thermal Free Energies = -1348.093382

Charge = 0 Multiplicity = 1
Au 1.654029 -1.573463 6.465590
O 2.152374 0.037959 5.457746
O 1.367262 -2.380889 4.616351
C 2.057694 -0.184044 4.116918
C 2.363130 0.831042 3.222128
C 2.267696 0.606345 1.842713
C 1.859570 -0.657930 1.413707
C 1.542860 -1.707169 2.291914
C 1.645607 -1.456278 3.671364
H 1.781838 -0.844834 0.350799
H 2.675107 1.792777 3.621276
C 1.659660 -1.723641 9.327422
C 1.934218 -0.813924 8.285348
C 1.822957 -1.317115 10.652906
C 2.360658 0.471392 8.569872
C 2.251786 -0.024141 10.928350
H 1.619468 -2.003749 11.469918
C 2.525349 0.885800 9.901154
H 2.570060 1.163536 7.757739
H 2.378662 0.288063 11.961310
C 1.221886 -3.046227 8.902151
C 0.883408 -4.122960 9.718750
N 1.153394 -3.193638 7.552256
H 0.933850 -4.012441 10.795845
C 0.773504 -4.345456 6.983102
C 0.487969 -5.321441 9.143178
H 0.754677 -4.355049 5.898400
H 0.223946 -6.163551 9.775115
C 0.431403 -5.440255 7.755446
H 0.127586 -6.361914 7.273239
C 2.980915 2.281982 10.203120
H 2.241439 3.015302 9.861553
H 3.135560 2.429430 11.274838
H 3.918900 2.509461 9.685264
C 1.099018 -3.079906 1.780597
C -0.309021 -3.394345 2.309085
C 2.086287 -4.152613 2.265924
H 3.088321 -3.965072 1.863868
H 2.149913 -4.159792 3.356503
H 1.767312 -5.145797 1.926909
H -0.329510 -3.374223 3.401129
H -0.630783 -4.386786 1.970521
H -1.033256 -2.660488 1.937737
C 2.609127 1.739255 0.871787
C 2.447643 1.320296 -0.590395
C 4.065335 2.177238 1.086634
H 4.234067 2.528900 2.109373
H 4.754503 1.346386 0.899639
H 4.325368 2.995204 0.404166
H 3.108680 0.486487 -0.850328
H 2.702327 2.161756 -1.243839
H 1.417978 1.023643 -0.817458
C 1.050617 -3.145624 0.252619
H 2.031998 -2.961555 -0.197943
H 0.727823 -4.146215 -0.055511
H 0.340279 -2.425144 -0.167289
C 1.681430 2.935150 1.132483
H 0.632830 2.655614 0.982354
H 1.785042 3.310986 2.155295
H 1.915277 3.759284 0.447959

4b-sq

Zero-point correction = 0.504245 (Hartree/Particle)
Thermal correction to Energy = 0.533739
Thermal correction to Enthalpy = 0.534683
Thermal correction to Gibbs Free Energy = 0.443732
Sum of electronic and zero-point Energies = -1347.866046
Sum of electronic and thermal Energies = -1347.836552
Sum of electronic and thermal Enthalpies = -1347.835608
Sum of electronic and thermal Free Energies = -1347.926559
[FINAL SINGLE POINT ENERGY = -20889.655197401724]

Charge = 1 Multiplicity = 2
Au 1.616709 -1.601426 6.518812
O 2.048995 0.046484 5.473737
O 1.446671 -2.380807 4.570259
C 2.027947 -0.146060 4.174925
C 2.320357 0.899931 3.292831
C 2.287712 0.660549 1.933905
C 1.950613 -0.652592 1.461425
C 1.654463 -1.720014 2.272490
C 1.694928 -1.471623 3.683821
H 1.932622 -0.801013 0.388874
H 2.564972 1.869718 3.708985
C 1.542868 -1.706699 9.374554
C 1.783678 -0.798796 8.327429
C 1.627261 -1.246114 10.688302
C 2.102103 0.521893 8.564070
C 1.948084 0.084600 10.932804
H 1.441963 -1.919146 11.520287
C 2.189794 0.983488 9.889850
H 2.284970 1.205490 7.739348
H 2.012670 0.435891 11.958528
C 1.221528 -3.063386 8.961942
C 0.945455 -4.154338 9.779742
N 1.201810 -3.229456 7.611681
H 0.962052 -4.031780 10.856300
C 0.920833 -4.410022 7.042210
C 0.653302 -5.383524 9.205728
H 0.927632 -4.440726 5.958307
H 0.435783 -6.237316 9.839365
C 0.638853 -5.518294 7.819092
H 0.413761 -6.463020 7.338657
C 2.535486 2.415832 10.160375
H 1.795081 3.086843 9.711182
H 2.576859 2.619546 11.232779
H 3.507004 2.671419 9.723500
C 1.298451 -3.102118 1.734133
C 0.114523 -3.487347 2.201508
C 2.320289 -4.132465 2.241157
H 3.330146 -3.876994 1.902788
H 2.327105 -4.193165 3.331593
H 2.071533 -5.122529 1.843851
H 0.183843 -3.528296 3.290730
H 0.376878 -4.473763 1.803996
H 0.854383 -2.767987 1.834513

C 2.593996 1.738708 0.906070
C 1.357742 1.941370 0.014131
C 3.781109 1.282281 0.041826
H 4.675355 1.126900 0.654161
H 3.568703 0.352722 -0.494682
H 4.006953 2.052704 -0.703047
H 1.083088 1.030024 -0.525571
H 1.567682 2.718113 -0.728900
H 0.494451 2.260771 0.607010
C 1.315791 -3.130569 0.205235
H 2.305840 -2.892531 -0.198350
H 1.052833 -4.136911 -0.135101
H 0.588465 -2.434260 -0.225925
C 2.950414 3.074048 1.557551
H 2.126177 3.464190 2.163730
H 3.839243 2.992225 2.191911
H 3.165577 3.810418 0.777289

5a

E(RPBE1PBE) = -1035.54807617
Zero-point correction = 0.301659 (Hartree/Particle)
Thermal correction to Energy = 0.319981
Thermal correction to Enthalpy = 0.320925
Thermal correction to Gibbs Free Energy = 0.255095
Sum of electronic and zero-point Energies = -1035.246417
Sum of electronic and thermal Energies = -1035.228095
Sum of electronic and thermal Enthalpies = -1035.227151
Sum of electronic and thermal Free Energies = -1035.292982

Charge = 0 Multiplicity = 1
Au -0.171216 -0.111962 0.033911
N 0.241437 0.064248 2.066797
O -0.496396 1.887239 -0.273112
O -0.561960 -0.276915 -1.894415
C 0.133068 -2.048168 0.345023
C 0.556222 -1.267043 2.611149
C 0.477967 -2.328536 1.692520
C 0.744179 -3.657730 2.120294
C 1.091540 -3.861144 3.478783
H 1.299811 -4.870040 3.824520
C 1.166600 -2.798494 4.349839
H 1.435980 -2.966883 5.387965
C 0.896322 -1.477633 3.923185
H 0.959818 -0.656943 4.630733
C -0.799695 2.073918 -1.569341
C -1.077602 3.333852 -2.100697
C -1.390867 3.473984 -3.455306
C -1.427940 2.358125 -4.286711
C -1.150641 1.089615 -3.768124
C -0.837831 0.947218 -2.420611
C 0.645471 -4.693054 1.156679
C 0.046837 -3.073490 -0.563441
H -0.216953 -2.871840 -1.597779
C 0.305169 -4.403354 -0.144240
H 0.842158 -5.717325 1.461208
H 0.231610 -5.206075 -0.872827
H -1.173764 0.205193 -4.399747
H -1.671674 2.464987 -5.340013
H -1.605484 4.461210 -3.855583
H -1.045440 4.196940 -1.440274
C 1.388447 0.995535 2.229800
H 1.571977 1.168495 3.293783
H 2.271419 0.556373 1.764485
H 1.138127 1.937932 1.740000
C -0.962536 0.633517 2.729827
H -1.204866 1.582363 2.248485
H -1.792946 -0.064493 2.619066
H -0.751375 0.798093 3.790171

5a-sq

E(UPBE1PBE) = -1035.37557831
Zero-point correction = 0.302310 (Hartree/Particle)
Thermal correction to Energy = 0.320805
Thermal correction to Enthalpy = 0.321749
Thermal correction to Gibbs Free Energy = 0.254117
Sum of electronic and zero-point Energies = -1035.073268
Sum of electronic and thermal Energies = -1035.054774
Sum of electronic and thermal Enthalpies = -1035.053829
Sum of electronic and thermal Free Energies = -1035.121462
[FINAL SINGLE POINT ENERGY = -20576.368776438227]

Charge = 1 Multiplicity = 2
Au -0.147269 -0.092273 0.076827
N 0.270404 0.066883 2.095988
O -0.550177 1.952811 -0.325037
O -0.539932 -0.231319 -1.892094
C 0.181103 -2.021281 0.363769
C 0.535460 -1.275095 2.646221
C 0.485265 -2.317900 1.712140
C 0.732250 -3.656342 2.116053
C 1.025914 -3.885200 3.482837
H 1.217366 -4.900615 3.817937
C 1.066583 -2.838771 4.376070
H 1.291691 -3.027851 5.420842
C 0.820323 -1.507527 3.966496
H 0.859533 -0.699842 4.690202
C -0.841136 2.103516 -1.573536
C -1.160437 3.364782 -2.136727
C -1.459833 3.441456 -3.470936
C -1.455667 2.279769 -4.299272
C -1.151482 1.038319 -3.794532
C -0.838395 0.923679 -2.427058
C 0.663174 -4.666210 1.123349
C 0.118278 -3.008982 -0.582201
H -0.116951 -2.786341 -1.618380
C 0.366101 -4.347854 -0.181247
H 0.847136 -5.698031 1.408613
H 0.315526 -5.132059 -0.930796
H -1.145594 0.148163 -4.414710
H -1.699624 2.385180 -5.351723
H -1.706104 4.400393 -3.915702
H -1.158959 4.237802 -1.492354
C 1.468640 0.939609 2.244075
H 1.681558 1.072697 3.307781
H 2.317080 0.465352 1.750485
H 1.257842 1.908988 1.789355
C -0.903552 0.695566 2.763177
H -1.104809 1.656057 2.285928
H -1.764252 0.034008 2.664824
H -0.672076 0.856173 3.819339

5b

E(RPBE1PBE) = -1349.72253786
Zero-point correction = 0.527317 (Hartree/Particle)
Thermal correction to Energy = 0.556831
Thermal correction to Enthalpy = 0.557775
Thermal correction to Gibbs Free Energy = 0.468241
Sum of electronic and zero-point Energies = -1349.195221
Sum of electronic and thermal Energies = -1349.165707
Sum of electronic and thermal Enthalpies = -1349.164763
Sum of electronic and thermal Free Energies = -1349.254297

Charge = 0 Multiplicity = 1
Au -0.202409 -0.104354 -0.022926
N 0.170623 0.093371 2.017203
O -0.508659 1.885434 -0.360586

O -0.567539 -0.281895 -1.949711
C 0.096013 -2.037543 0.316044
C 0.478544 -1.231286 2.580635
C 0.418692 -2.303041 1.672266
C 0.681899 -3.626585 2.119295
C 1.005498 -3.814295 3.485912
H 1.210910 -4.818702 3.846179
C 1.061404 -2.742156 4.346745
H 1.312353 -2.898609 5.391355
C 0.795301 -1.426848 3.900797
H 0.844209 -0.598281 4.600433
C -0.805348 2.076505 -1.664487
C -1.073237 3.341655 -2.217203
C -1.382558 3.404825 -3.585835
C -1.432044 2.282804 -4.414424
C -1.152473 1.034562 -3.843230
C -0.842853 0.937287 -2.494636
C 0.604776 -4.672603 1.165288
C 0.030179 -3.073792 -0.582120
H -0.215742 -2.884487 -1.623236
C 0.286791 -4.397996 -0.144558
H 0.800494 -5.692700 1.484185
H 0.230285 -5.208711 -0.865820
H -1.171957 0.122333 -4.434058
H -1.592986 4.375767 -4.014382
C 1.309899 1.030975 2.195566
H 1.472421 1.213024 3.261478
H 2.203773 0.593193 1.750278
H 1.063957 1.968240 1.694000
C -1.048047 0.663904 2.650931
H -1.282333 1.608742 2.157823
H -1.874591 -0.036591 2.527504
H -0.859638 0.836225 3.714369
C -1.023866 4.599227 -1.345633
C 0.383896 4.754458 -0.749829
C -2.054972 4.482474 -0.212662
H -3.069557 4.407521 -0.620006
H -1.863107 3.596845 0.397426
H -2.014926 5.368541 0.432602
H 0.655342 3.876057 -0.159751
H 0.429440 5.639142 -0.103052
H 1.127978 4.879257 -1.544610
C -1.780877 2.366474 -5.902357
C -3.041332 1.533582 -6.178882
C -0.617773 1.808768 -6.735810
H 0.297301 2.386283 -6.564470
H -0.406629 0.764182 -6.486125
H -0.854460 1.855496 -7.805486
H -2.896347 0.482302 -5.910634
H -3.304050 1.574757 -7.242728
H -3.893235 1.912175 -5.603383
C -1.339455 5.870605 -2.136564
H -0.623117 6.037967 -2.948266
H -2.346257 5.847349 -2.567598
H -1.286979 6.735233 -1.465759
C -2.047999 3.802299 -6.356960
H -1.172754 4.444605 -6.211317
H -2.291628 3.810348 -7.424895
H -2.892307 4.248845 -5.820756

5b-sq

E(UPBE1PBE) = -1349.55626291
Zero-point correction = 0.528172 (Hartree/Particle)
Thermal correction to Energy = 0.557836
Thermal correction to Enthalpy = 0.558781
Thermal correction to Gibbs Free Energy = 0.468006
Sum of electronic and zero-point Energies = -1349.028091
Sum of electronic and thermal Energies = -1348.998427
Sum of electronic and thermal Enthalpies = -1348.997482
Sum of electronic and thermal Free Energies = -1349.088257
[FINAL SINGLE POINT ENERGY = -20890.837560477463]

Charge = 1 Multiplicity = 2
Au -0.141403 -0.100509 0.008003
N 0.192106 0.059601 2.044131
O -0.507103 1.937486 -0.401769
O -0.489420 -0.230701 -1.959125
C 0.166797 -2.029843 0.308011
C 0.426915 -1.283836 2.604316
C 0.413702 -2.327196 1.668350
C 0.638503 -3.666361 2.083312
C 0.870185 -3.896294 3.461786
H 1.043469 -4.912355 3.804859
C 0.873028 -2.850331 4.356421
H 1.049809 -3.040624 5.410260
C 0.649733 -1.518346 3.936205
H 0.656940 -0.710887 4.661275
C -0.778668 2.111802 -1.653542
C -1.066094 3.397377 -2.219888
C -1.369391 3.420270 -3.561268
C -1.406813 2.262358 -4.399531
C -1.104954 1.023808 -3.855356
C -0.786199 0.929912 -2.502326
C 0.609470 -4.677028 1.089236
C 0.140640 -3.019579 -0.637953
H -0.052130 -2.796008 -1.682772
C 0.368673 -4.358771 -0.226977
H 0.778489 -5.709069 1.382970
H 0.348004 -5.143473 -0.977509
H -1.113261 0.116065 -4.449190
H -1.596894 4.373013 -4.018484
C 1.384221 0.929557 2.246066
H 1.553835 1.061111 3.317727
H 2.252271 0.456025 1.787000
H 1.193633 1.900064 1.784600
C -1.006878 0.693339 2.659102
H -1.865968 0.036364 2.522683
H -0.821352 0.853512 3.724282
H -1.183396 1.654715 2.173939
C -1.029584 4.654764 -1.357468
C 0.374424 4.824209 -0.754031
C -2.073810 4.542714 -0.234808
H -3.081096 4.431778 -0.650144
H -1.873964 3.690923 0.418830
H -2.057483 5.453407 0.373748
H 0.642655 3.983007 -0.111161
H 0.406921 5.739215 -0.152759
H 1.128862 4.911689 -1.543179
C -1.779293 2.361899 -5.875028
C -3.037936 1.513878 -6.120313
C -0.621071 1.812006 -6.722452
H 0.294994 2.388902 -6.558196
H -0.409674 0.763415 -6.493502
H -0.879669 1.878098 -7.784644
H -2.871881 0.461156 -5.873721
H -3.321065 1.572670 -7.176722
H -3.879589 1.876816 -5.521168
C -1.345507 5.907039 -2.176847
H -0.621037 6.062513 -2.983425
H -2.348788 5.869378 -2.614192
H -1.303527 6.781622 -1.520353
C -2.066268 3.797114 -6.318276
H -1.194314 4.447403 -6.191663
H -2.326244 3.797216 -7.381193
H -2.908616 4.235166 -5.772601

8. References

[1] Stoll, S.; Schweiger, A. *J. Magn. Reson.* 2006, **178**, 42.

[2] The MathWorks, Inc. *MATLAB*; The MathWorks, Inc.: Natick, MA, 2024.

[3] Shaw, A. P.; Tilset, M.; Heyn, R. H.; Jakobsen, S. *J. Coord. Chem.* 2011, **64**, 38–47.

[4] Blons, C.; Mallet-Ladeira, S.; Amgoune, A.; Bourissou, D. *Angew. Chem. Int. Ed.* 2018, **57**, 11732–11736.

[5] León, F.; García-Rodeja, Y.; Mallet-Ladeira, S.; Miqueu, K.; Szaloki, G.; Bourissou, D. *Chem. Sci.* 2024, **15**, 16651–16659.

[6] Frisch, M. J.; Trucks, G. W.; Schlegel, H. B.; Scuseria, G. E.; Robb, M. A.; Cheeseman, J. R.; Scalmani, G.; Barone, V.; Petersson, G. A.; Nakatsuji, H.; Li, X.; Caricato, M.; Marenich, A. V.; Bloino, J.; Janesko, B. G.; Gomperts, R.; Mennucci, B.; Hratchian, H. P.; Ortiz, J. V.; Izmaylov, A. F.; Sonnenberg, J. L.; Williams-Young, D.; Ding, F.; Lipparini, F.; Egidi, F.; Goings, J.; Peng, B.; Petrone, A.; Henderson, T.; Ranasinghe, D.; Zakrzewski, V. G.; Gao, J.; Rega, N.; Zheng, G.; Liang, W.; Hada, M.; Ehara, M.; Toyota, K.; Fukuda, R.; Hasegawa, J.; Ishida, M.; Nakajima, T.; Honda, Y.; Kitao, O.; Nakai, H.; Vreven, T.; Throssell, K.; Montgomery, J. A., Jr.; Peralta, J. E.; Ogliaro, F.; Bearpark, M. J.; Heyd, J. J.; Brothers, E. N.; Kudin, K. N.; Staroverov, V. N.; Keith, T. A.; Kobayashi, R.; Normand, J.; Raghavachari, K.; Rendell, A. P.; Burant, J. C.; Iyengar, S. S.; Tomasi, J.; Cossi, M.; Millam, J. M.; Klene, M.; Adamo, C.; Cammi, R.; Ochterski, J. W.; Martin, R. L.; Morokuma, K.; Farkas, O.; Foresman, J. B.; Fox, D. J. *Gaussian 16, Revision B.01*; Gaussian, Inc.: Wallingford, CT, 2016.

[7] (a) Neese, F. *WIREs Comput. Mol. Sci.* 2012, **2**, 73–78. (b) Neese, F. *WIREs Comput. Mol. Sci.* 2022, **12**, e1606. (c) Neese, F. *WIREs Comput. Mol. Sci.* 2025, **15**, e70019.

[8] Marenich, A. V.; Cramer, C. J.; Truhlar, D. G. *J. Phys. Chem. B* 2009, **113**, 6378–6396.

[9] Adamo, C.; Barone, V. *J. Chem. Phys.* 1999, **110**, 6158–6170.

[10] Grimme, S.; Antony, J.; Ehrlich, S.; Krieg, H. A. *J. Chem. Phys.* 2010, **132**, 154104.

[11] Grimme, S.; Ehrlich, S.; Goerigk, L. *J. Comput. Chem.* 2011, **32**, 1456–1465.

[12] (a) Ditchfield, R.; Hehre, W. J.; Pople, J. A. *J. Chem. Phys.* 1971, **54**, 724–728. (b) Hariharan, P. C.; Pople, J. A. *Theor. Chim. Acta* 1973, **28**, 213–222. (c) Hehre, W. J.; Ditchfield, R.; Pople, J. A. *J. Chem. Phys.* 1972, **56**, 2257–2261. (d) Gordon, M. S.; Binkley, J. S.; Pople, J. A.; Pietro, W. J.; Hehre, W. J. *J. Am. Chem. Soc.* 1982, **104**, 2797–2803. (e) Francl, M. M.; Pietro, W. J.; Hehre, W. J.; Binkley, J. S.; Gordon, M. S.; DeFrees, D. J.; Pople, J. A. *J. Chem. Phys.* 1982, **77**, 3654–3665. (f) Spitznagel, G. W.; Clark, T.; von Ragué Schleyer, P.; Hehre, W. J. *J. Comput. Chem.* 1987, **8**, 1109–1116.

[13] Andrae, D.; Häussermann, U.; Dolg, M.; Stoll, H.; Preuss, H. *Theor. Chim. Acta* 1990, **77**, 123–141.

[14] Ehlers, A. W.; Böhme, M.; Dapprich, S.; Gobbi, A.; Höllwarth, A.; Jonas, V.; Köhler, K. F.; Stegmann, R.; Veldkamp, A.; Frenking, G. *Chem. Phys. Lett.* 1993, **208**, 111–114.

[15] Wolinski, K.; Hinton, J. F.; Pulay, P. *J. Am. Chem. Soc.* 1990, **112**, 8251–8260.

[16] (a) Caldeweyher, E.; Bannwarth, C.; Grimme, S. *J. Chem. Phys.* 2017, **147**, 034112. (b) Caldeweyher, E.; Ehlert, S.; Hansen, A.; Neugebauer, H.; Spicher, S.; Bannwarth, C.; Grimme, S. *J. Chem. Phys.* 2019, **150**, 154122.

[17] van Wüllen, C. *J. Chem. Phys.* 1998, **109**, 392–399.

[18] Weigend, F.; Ahlrichs, R. *Phys. Chem. Chem. Phys.* 2005, **7**, 3297–3305.

[19] Pantazis, D. A.; Chen, X.-Y.; Landis, C. R.; Neese, F. *J. Chem. Theory Comput.* 2008, **4**, 908–919.

[20] (a) Whitten, J. L. *J. Chem. Phys.* 1973, **58**, 4496–4501. (b) Dunlap, B. I.; Connolly, J. W. D.; Sabin, J. R. *J. Chem. Phys.* 1979, **71**, 3396–3402. (c) Neese, F. *J. Comput. Chem.* 2003, **24**, 1740–1747.

[21] Neese, F.; Wennmohs, F.; Hansen, A.; Becker, U. *Chem. Phys.* 2009, **356**, 98–109.

[22] Weigend, F. *Phys. Chem. Chem. Phys.* 2006, **8**, 1057–1065.

[23] Zhurko, G. A. *Chemcraft*, version 1.8, build 622b; Ivanovo, Russia, 2005. https://chemcraftprog.com

[24] Bard, A. J.; Faulkner, L. R.; White, H. S. *Electrochemical Methods: Fundamentals and Applications*; John Wiley & Sons: New York, 2001.

[25] Renny, J. S.; Tomasevich, L. L.; Tallmadge, E. H.; Collum, D. B. *Angew. Chem. Int. Ed.* 2013, **52**, 11998–12013.

[26] Hibbert, D. B.; Thordarson, P. *Chem. Commun.* 2016, **52**, 12792–12805.

[27] Hari, D. P.; Schroll, P.; König, B. *J. Am. Chem. Soc.* 2012, **134**, 2958–2961.
